# Combinatorial prediction of therapeutic perturbations using causally-inspired neural networks

**DOI:** 10.1101/2024.01.03.573985

**Published:** 2024-10-07

**Authors:** Guadalupe Gonzalez, Xiang Lin, Isuru Herath, Kirill Veselkov, Michael Bronstein, Marinka Zitnik

**Affiliations:** 1 Imperial College London, London, UK; 2 Prescient Design, Genentech, South San Francisco, CA, USA; 3 F. Hoffmann-La Roche Ltd, Basel, Switzerland; 4 Merck & Co., South San Francisco, CA, USA; 5 Cornell University, Ithaca, NY, USA; 6 University of Oxford, Oxford, UK; 7 Harvard Medical School, Boston, MA, USA; 8 Kempner Institute for the Study of Natural and Artificial Intelligence, Harvard University, Cambridge, MA, USA; 9 Broad Institute of MIT and Harvard, Cambridge, MA, USA; 10 Harvard Data Science Initiative, Cambridge, MA, USA

## Abstract

As an alternative to target-driven drug discovery, phenotype-driven approaches identify compounds that counteract the overall disease effects by analyzing phenotypic signatures. Our study introduces a novel approach to this field, aiming to expand the search space for new therapeutic agents. We introduce PDGrapher, a causally-inspired graph neural network (GNN) designed to predict combinatorial perturbagens – sets of therapeutic targets – capable of reversing disease effects. Unlike methods that learn responses to perturbations, PDGrapher solves the inverse problem, which is to infer the perturbagens necessary to achieve a specific response – i.e., directly predicting perturbagens by learning which perturbations elicit a desired response. By encoding gene regulatory networks or protein-protein interactions, PDGrapher can predict unseen chemical or genetic perturbagens, aiding in the discovery of novel drugs or therapeutic targets. Experiments across nine cell lines with chemical perturbations show that PDGrapher successfully predicted effective perturbagens in up to 13.33% additional test samples and ranked therapeutic targets up to 35% higher than the competing methods, and the method shows competitive performance across ten genetic perturbation datasets. A key innovation of PDGrapher is its direct prediction capability, which contrasts with the indirect, computationally intensive models traditionally used in phenotype-driven drug discovery that only predict changes in phenotypes due to perturbations. The direct approach enables PDGrapher to train up to 25 times faster than methods like scGEN and CellOT, representing a considerable leap in efficiency. Our results suggest that PDGrapher can advance phenotype-driven drug discovery, offering a fast and comprehensive approach to identifying therapeutically useful perturbations.

Target-driven drug discovery, predominant since the 1990s, focuses on the design of highly specific compounds against disease-associated targets such as proteins or enzymes [1, 2]. A prime example of target-driven drug discovery is the development of small molecule kinase inhibitors like Imatinib. Imatinib halts the progression of chronic myeloid leukemia (CML) by inhibiting the BCR-ABL tyrosine kinase, a mutated protein that drives the uncontrolled proliferation of leukemic cells in CML patients [3]. Another notable example is monoclonal antibodies like Trastuzumab, which specifically targets the HER2 receptor, a protein overexpressed in certain types of breast cancer. Trastuzumab inhibits cell proliferation while engaging the body’s immune system to initiate an attack against the cancer [4]. These examples illustrate the success of target-driven drug discovery, yet the past decade has seen a revival of phenotype-driven approaches. This shift has been fueled by the observation that many first-in-class drugs approved by the US Food and Drug Administration (FDA) between 1999 and 2008 were discovered empirically without a drug target hypothesis [5]. Instead of the “one drug, one gene, one disease” model of target-driven approaches, phenotype-driven drug discovery aims to identify compounds or, more broadly, perturbagens – combinations of therapeutic targets – that reverse phenotypic disease effects as measured by high-throughput phenotypic assays [1,6]. A recent success is ivacaftor, the first available therapy treating the underlying cause of cystic fibrosis [7–9].

The resurgence of phenotype-driven drug discovery has been further bolstered by the advent of chemical and genetic libraries like the Connectivity Map (CMap [10]) and the Library of Integrated Network-based Cellular Signatures (LINCS [11]). CMap and LINCS contain gene expression profiles of dozens of cell lines treated with thousands of genetic and chemical perturbagens. CMap introduced the concept of “connectivity score” to quantify similarities between compound response and disease gene expression signatures. The hypothesis is that identifying compounds with gene expression signatures akin to known disease-treating drugs or counteracting disease signatures could unveil new therapeutic leads [12–15]. These strategies have been successful in identifying drugs with high efficacy in vitro [15] against new or under-studied diseases [16–18].

Recent advances in deep learning produced methods that predict gene expression responses to perturbagens or combinations thereof [19–22]. Such methods have advanced lead discovery by enabling predictions of responses to perturbagens that were not yet experimentally tested. 1) However, current approaches rely on predefined chemical and genetic libraries, meaning they can select perturbagens only from predefined libraries instead of flexibly identifying perturbagens as combinations of therapeutic targets. 2) Existing approaches are predominantly perturbation response methods that predict changes in phenotypes upon perturbations. Thus, they only indirectly identify perturbagens by exhaustively predicting responses to all perturbations in the library and then searching for perturbagens with the desired response. 3) Unlike existing methods that learn responses to perturbations, phenotype-driven drug discovery needs to solve the inverse problem, which is to infer perturbagens necessary to achieve a specific response – i.e., directly predicting perturbagens by learning which perturbations elicit a desired response.

In causal discovery, the problem of identifying which elements of a system should be perturbed to achieve a desired state is referred to as *optimal intervention design* [23–25]. Leveraging insights from causal discovery and geometric deep learning, here we introduce PDGrapher, a novel approach for combinatorial prediction of therapeutic targets that can shift gene expression from an initial, diseased state to a desired treated state. PDGrapher is formulated using a causal model, where genes represent the nodes in a causal graph, and structural causal equations define their causal relationships. Given a genetic or chemical intervention dataset, PDGrapher pinpoints a set of genes that a perturbagen should target to facilitate the transition of node states from diseased to treated. PDGrapher utilizes protein-protein interaction networks (PPI) and gene regulatory networks (GRN) as approximations of the causal graph, operating under the assumption of no unobserved confounders. PDGrapher tackles the optimal intervention design objective using representation learning, utilizing a graph neural network (GNN) to represent the structural equations.

Given pairs of diseased and treated samples, PDGrapher is trained to output a ranking of genes, with the top-predicted genes identified as candidate combinatorial therapeutic targets to shift gene expression phenotype from a diseased to a normal state in each sample. Once trained, PDGrapher takes a new diseased sample and returns a perturbagen – a combination or set of therapeutic targets – that are likely to counter disease effects in the given sample. We evaluate PDGrapher across 19 datasets, comprising genetic and chemical interventions across 11 cancer types and proxy causal graphs, and consider diverse evaluation setups, including settings where held out folds contain novel samples and challenging settings where held out folds contain novel samples from a cancer type that PDGrapher has never encountered before. In held out folds that contain novel samples, PDGrapher ranks ground-truth therapeutic targets up to 8.27% higher in chemical intervention datasets and 0.68% higher in genetic intervention datasets than existing methods. Even in held-out folds containing novel samples from a previously unseen disease, PDGrapher maintains robust performance. A key innovative feature of PDGrapher is its direct prediction of perturbagens that can shift gene expression from diseased to treated states in contrast with existing methods that indirectly predict perturbagens through extensive computational modeling of cell responses. This PDGrapher’s feature enables model training up to 25 times faster than indirect prediction methods like scGen [21] and CellOT [26]. Since these approaches build a separate model for each perturbation, they become almost unusable for data with many perturbagens. For example, with the default setting, cellOT takes 10 hours to train a model for a single perturbagen in one cell line from the LINCS dataset. We find that in chemical intervention datasets, candidate therapeutic targets predicted by PDGrapher are on average up to 11.58% closer to ground-truth therapeutic targets in the gene-gene interaction network than what would be expected by chance. Additionally, PDGrapher can aid in elucidating the mechanism of action of chemical perturbagens (Figure S6), which we show in the case of vorinonstat, a histone deacetylase inhibitor used to treat cutaneous T-cell lymphoma, and sorafeniv, a multi-kinase inhibitor used in the treatment of several types of cancers (Supplementary Note 1). PDGrapher can also identify novel therapeutic targets and drugs specific to certain cancer types. We demonstrate this with the top-predicted target, TOP2A, for non-small cell lung cancer (A549), along with the associated drugs aldoxorubicin, vosaroxin, and doxorubicin Hydrochloride, which are currently in early clinical development stages. Results underscore PDGrapher’s effectiveness in identifying perturbagens as combinatorial therapeutic targets that transition cells from a diseased to a treated state, highlighting PDGrapher’s potential to enhance phenotype-driven drug discovery.

## Results

### Overview of datasets and PDGrapher.

We consider a total of 19 datasets across two treatment types (genetic and chemical interventions), 11 cancer types (lung cancer, breast cancer, prostate cancer, colon cancer, skin cancer, cervical cancer, head and neck cancer, pancreatic cancer, stomach cancer, brain cancer, and ovarian cancer), and two proxy causal graphs (protein-protein interaction network (PPI), and gene regulatory networks (GRN)), which we denote as follows: Chemical-PPI-Lung-A549, Chemical-PPI-Breast-MCF7, Chemical-PPI-Breast-MDAMB231, Chemical-PPI-Breast-BT20, Chemical-PPI-Prostate-PC3, Chemical-PPI-Prostate-VCAP, Chemical-PPI-Colon-HT29, Chemical-PPI-Skin-A375, and Chemical-PPI-Cervix-HELA, Genetic-PPI-Lung-A549, Genetic-PPI-Breast-MCF7, Genetic-PPI-Prostate-PC3, Genetic-PPI-Skin-A375, Genetic-PPI-Colon-HT29, Genetic-PPI-Ovary-ES2, Genetic-PPI-Head-BICR6, Genetic-PPI-Pancreas-YAPC, Genetic-PPI-stomach-AGS, and Genetic-PPI-Brain-U251MG, Chemical-GRN-Lung-A549, Chemical-GRN-Breast-MCF7, Chemical-GRN-Breast-MDAMB231, Chemical-GRN-Breast-BT20, Chemical-GRN-Prostate-PC3, Chemical-GRN-Prostate-VCAP, Chemical-GRN-Colon-HT29, Chemical-GRN-Skin-A375, and Chemical-GRN-Cervix-HELA, Genetic-GRN-Lung-A549, Genetic-GRN-Breast-MCF7, Genetic-GRN-Prostate-PC3, Genetic-GRN-Skin-A375, Genetic-GRN-Colon-HT29, Genetic-GRN-Ovary-ES2, Genetic-GRN-Head-BICR6, Genetic-GRN-Pancreas-YAPC, Genetic-GRN-Stomach-AGS, and Genetic-GRN-Brain-U251MG. Genetic interventions are single-gene knockout experiments by CRISPR/Cas9-mediated gene knockouts, while chemical interventions are multiple-gene treatments induced using chemical compounds. We utilize a PPI network that has 10,716 nodes and 151,839 undirected edges. We additionally construct gene regulatory networks for each disease-treatment type pair using a gene regulatory network inference method (Online Methods) with GRNs on average having 10 thousand nodes and 500 thousand directed edges. Each dataset is made up of disease intervention data, if available, and treatment intervention data. For cell lines corresponding to lung, breast, and prostate cancer, the disease intervention data contains paired healthy and diseased gene expression samples and disease-associated genes. For the remaining cancer types, healthy control samples are not available,. Treatment intervention data contains paired diseased and treated gene expression samples and genetic or chemical perturbagens. Tables 1 and 2 summarize the number of samples for each cell line and intervention dataset type. Tables S1 and S2 show the numbers of unseen perturbagens within and across cell lines in chemical perturbation data, respectively; Table S3 and S4 show the number of unseen perturbagens within and across cell lines in genetic perturbation data, respectively.

**Figure 1:**
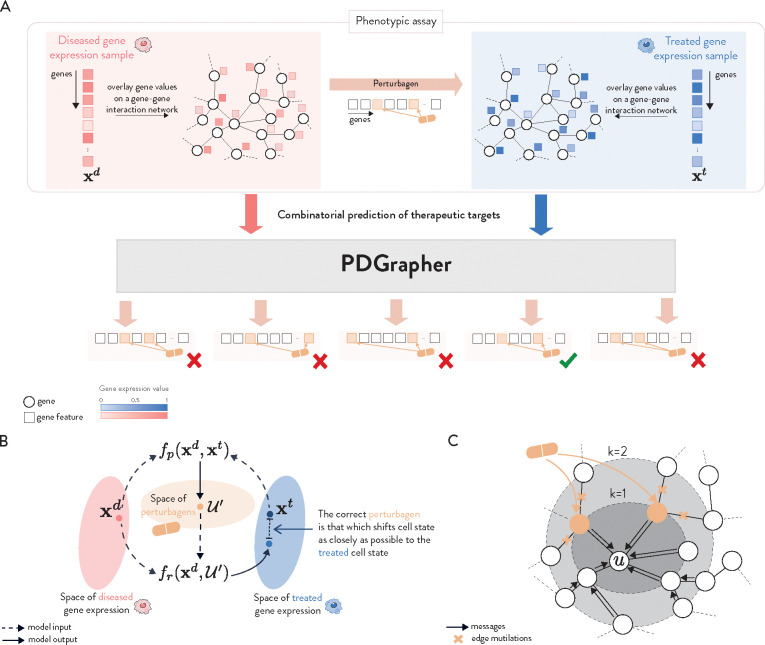
Overview of PDGrapher. **(A)** Given a paired diseased and treated gene expression samples and a proxy causal graph, PDGrapher predicts a candidate set of therapeutic targets to shift cell gene expression from diseased to treated state. **(B)** PDGrapher is comprised by two modules. A perturbagen discovery module $f_{p}$ that, given a pair of diseased and treated gene expression samples, computes a candidate set of therapeutic targets ${𝒰}^{\prime }$, and a response prediction module $f_{r}$ that predicts the response of the diseased sample to the predicted candidate perturbagen. $f_{p}$ is optimized using 2 losses: a cross-entropy cycle loss to predicted a perturbagen ${𝒰}^{\prime }$ which would shift diseased cell state to a state as close as possible to the treated state: $CE\left(x^{t},f_{r}\left(x^{d},f_{p}\left(x^{d},x^{t}\right)\right)\right.$) $\left(with\;f_{r}\;frozen\right)$, and a cross-entropy supervision loss that directly supervises the prediction of ${𝒰}^{\prime }:CE\left({𝒰}^{\prime },f_{p}\left(x^{d},x^{t}\right)\right)$. See Methods for more details. **(C)** PDGrapher has two modules, $f_{r}$ and $f_{p}$, both based on GNNs. Depicted is $f_{r}$, which takes as input a diseased gene expression and a perturbagen (therapeutic gene set) and represents perturbagens effects in the graph as edge mutilations. Both $f_{r}$ and $f_{p}$ follow the standard message-passing framework where node representations are updated by aggregating the information from neighbors in the graph.

Given a diseased cell state (gene expression), the goal of PDGrapher is to predict the genes that, if targeted by a perturbagen on the diseased cell, would shift the cell to a treated state (Figure 1A). Unlike methods for learning the response of cells to a given perturbation [21, 26–28], PDGrapher focuses on the inverse problem by learning which perturbation elicits a desired response. PDGrapher predicts perturbagens to shift cell states under the assumption that an optimal perturbagen will be that which shifts the cell gene expression as close as possible to the desired gene expression. Our approach comprises two modules (Figure 1B). First, a perturbagen discovery module $f_{p}$ takes the initial and desired cell states and outputs a candidate perturbagen as a set of therapeutic targets ${𝒰}^{\prime }$. Then, a response prediction module $f_{r}$ takes the initial state and the predicted perturbagen ${𝒰}^{\prime }$ and predicts the cell response upon perturbing genes in ${𝒰}^{\prime }$. Our response prediction and perturbagen discovery modules are graph neural network (GNN) models that operate on a proxy causal graph, where edge mutilations represent the effects of interventions on the graph (Figure 1C).

PDGrapher is trained using an objective function with two components, one for each module, $f_{r}$ and $f_{p}$. The response prediction module $f_{r}$ is trained using all available data on cell state transitions (that is, disease and treatment intervention data). Response prediction module $f_{r}$ is trained so cell states are close to the known perturbed cell states upon interventions. The perturbagen discovery module $f_{p}$ is trained using treatment intervention data; given a diseased cell state, $f_{p}$ predicts the set of therapeutic targets ${𝒰}^{\prime }$ that caused the corresponding treated cell state. The objective function for the perturbagen discovery module consists of two elements: (1) a cycle loss that optimizes the parameters of $f_{p}$ such that the response upon intervening on the predicted genes in ${𝒰}^{\prime }$, as measured by $f_{r}$, is as close as possible to the real treated state and (2) a supervision loss on the therapeutic targets set ${𝒰}^{\prime }$ that directly pushes PDGrapher to predict the correct perturbagen. Both models are trained simultaneously using early stopping independently so that each model finishes training upon convergence.

When trained, PDGrapher predicts perturbagens (as a set of candidate target genes) to shift cells from diseased to treated. An example of PDGrapher’s predictions is depicted in Figure S5, where we used SAFE [29] to visualize the spatial enrichment of ground-truth therapeutic targets of approved drugs against breast cancer and the top 10, 50 and 100 predicted perturbagen targets to shift breast cancer cell line MCF7 gene expression from a diseased to a healthy state. We observe a consistent pattern between targets of approved drugs and predicted gene targets and their spatial enrichment distributions.

**Figure 2:**
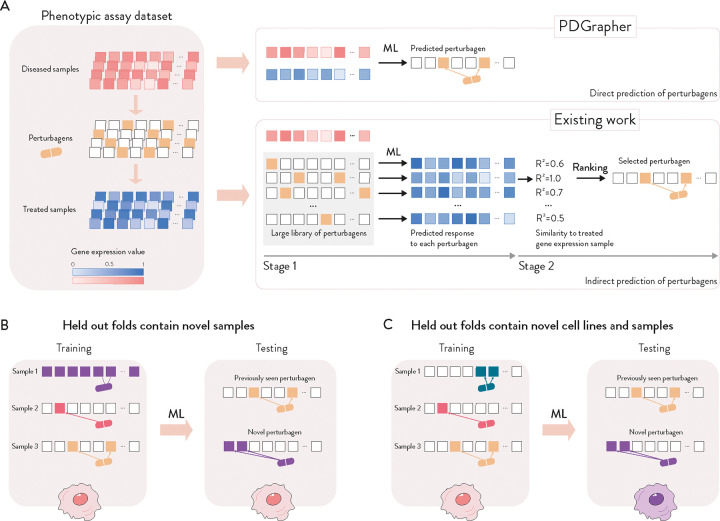
Overview of evaluation settings and data splits. **(A)** Given a dataset with paired diseased and treated samples and a set of perturbagens, PDGrapher makes a direct prediction of candidate perturbagens that shift gene expression from a diseased to a treated state, for each disease-treated sample pair. The direct prediction means that PDGrapher directly infers the perturbation necessary to achieve a specific response. In contrast to direct prediction of perturbagens, existing methods predict perturbagens only indirectly through a two-stage approach: for a given diseased sample, they learn the response to each one of the perturbagen candidates from an existing library upon intervention and return the perturbagen whose response is as close as possible to the desired treated state. Existing methods learn the response of cells to a given perturbation [21, 26–28], whereas PDGrapher focuses on the inverse problem by learning which perturbagen elicit a given response, even in the most challenging cases when the combinatorial composition of perturbagen was never seen before. **(B-C)** We evaluate PDGrapher’s performance across two settings: randomly splitting samples between training and test set (B), and splitting samples based on the cell line where we train in a cell line and evaluate PDGrapher’s performance on another cell line the model never encountered before (C).

Given a pair of diseased and treated samples, PDGrapher directly predicts perturbagens by learning which perturbations elicit target responses. In contrast, existing approaches are perturbation response methods that predict changes in phenotype that occur upon perturbation, thus, they can only indirectly predict perturbagens (Figure 2A). Given a disease-treated sample pair, a response prediction module (such as scGen [21], ChemCPA [30], Biolord [31], GEARS [32], and CellOT [26]) is used to predict the response of the diseased sample to a library of perturbagens. The predicted perturbagen is the one that produces a response that is the most similar to the treated sample. We evaluate PDGrapher’s performance in two separate settings (Figure 2BC): (1) a random splitting setting, where the samples are split randomly between training and test sets, and (2) a leave-cell-out setting, where PDGrapher is trained in one cell line, and its performance is evaluated in a cell line the model never encountered during training to test how well the model generalizes to a new disease.

### PDGrapher efficiently predicts genetic and chemical perturbagens to shift cells from diseased to treated states.

**Figure 3:**
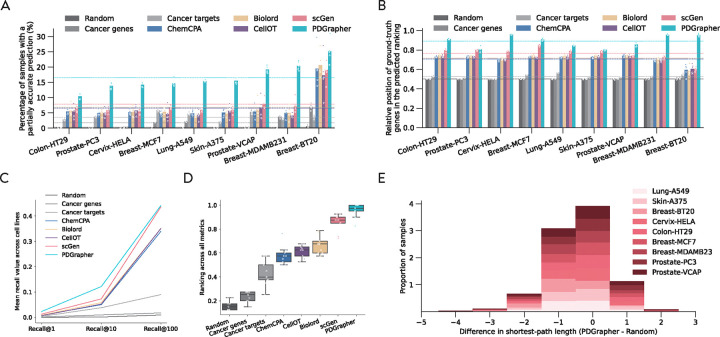
PDGrapher efficiently predicts chemical perturbagens to shift cells from diseased to treated states in held out folds containing novel samples. **(A)** PDGrapher consistently predicts ground-truth gene targets at the top of its ranked list of genes for Chemical-PPI datasets with up to 13.33% (Chemical-PPI-Breast-MDAMB231: 20.43% vs 7.10%) more samples in the test set compared to the second-best baseline **(B)** PDGrapher ranks ground-truth targets up to 34.81% (Chemical-PPI-MDAMB231: 95.44% vs 60.63%) higher than the second-best baseline in the predicted list across Chemical-PPI datasets. **(C)** PDGrapher recovers ground-truth therapeutic targets at higher rates compared to competing methods for Chemical-PPI datasets.**(D)** The boxplots show the ranks across the experiments of different cell lines and metrics for each method. Higher value indicates better performance. The central line inside the box represents the median, while the top and bottom edges correspond to the first (Q1) and third (Q3) quartiles. The whiskers extend to the smallest and largest values within 1.5 times the interquartile range (IQR) from the quartiles. Each individual dot represents a data point for a specific cell line and metrics. **(E)** Shown is the difference of shortest-path distances between ground-truth therapeutic genes and predicted genes by PDGrapher and a Random baseline, with negative values indicating that PDGrapher predicts sets of therapeutic genes that are closer in the network to ground-truth therapeutic genes compared to what would be expected by chance [average shortest-path distances across cell lines for PDGrapher vs Random = 2.77 vs 3.11].

In the random splitting setting, we assess the ability of PDGrapher for combinatorial prediction of therapeutic targets across chemical PPI datasets (Chemical-PPI-Lung-A549, Chemical-PPI-Breast-MCF7, Chemical-PPI-Breast-MDAMB231, Chemical-PPI-Breast-BT20, Chemical-PPI-Prostate-PC3, Chemical-PPI-Prostate-VCAP, Chemical-PPI-Colon-HT29, Chemical-PPI-Skin-A375, and Chemical-PPI-Cervix-HELA). Specifically, we measure whether, given paired diseased-treated gene expression samples, PDGrapher can predict the set of therapeutic genes targeted by the chemical compound in the diseased sample to generate the treated sample. Given paired diseased-treated gene expression samples, PDGrapher ranks genes in the dataset according to their likelihood of being in the set of therapeutic targets. We quantify the ranking of ground-truth gene targets in the predicted ranked list such that a ranking equal to one signifies perfectly accurate predictions by the model (the top K-ranked gene targets correspond to the K ground-truth gene targets), and zero signifies the opposite (the bottom K ranked gene targets correspond to the K ground-truth gene targets). PDGrapher ranks ground-truth gene targets 5.85% (Chemical-PPI-Lung-MCF7), 5.85% (Chemical-PPI-Lung-A549), 23.23% (Chemical-PPI-Breast-MDAMB231), 34.81% (Chemical-PPI-Breast-BT20), 0.92% (Chemical-PPI-Prostate-PC3), 11.13% (Chemical-PPI-Prostate-VCAP), 11.69% (Chemical-PPI-Colon-HT29), 1.39% (Chemical-PPI-Skin-A375), and 17.66% (Chemical-PPI-Cervix-HELA) higher in the list compared to the second best-performing method, respectively (Figure 3B). In addition to evaluating the entire predicted target rank, it is even more practically crucial to assess the accuracy of the top-ranked predicted targets. To achieve this, we use partial accuracy and recall to evaluate the performance of PDGrapher and competing methods. Because perturbagens target multiple genes, we measure the fraction of samples in the test set for which we obtain a partially accurate prediction, where at least one of the top N predicted gene targets corresponds to an actual gene target. Here, N represents the number of known target genes of a drug. PDGrapher consistently provides accurate predictions for more samples in the test set than baselines. Specifically, it outperforms the second-best baseline by predicting ground-truth targets in an additional 7.85% (Chemical-PPI-Lung-MCF7), 9.37% (Chemical-PPI-Lung-A549), 13.33% (Chemical-PPI-Breast-MDAMB231), 4.51% (Chemical-PPI-Breast-BT20), 7.92% (Chemical-PPI-Prostate-PC3), 11.36% (Chemical-PPI-Prostate-VCAP), 4.01% (Chemical-PPI-Colon-HT29), 9.53% (Chemical-PPI-Skin-A375), and 8.41% (Chemical-PPI-Cervix-HELA) of samples (Figure 3A). We also evaluate the performance of PDGrapher using recall@1, recall@10, and recall@100, which calculate the ratio of true target genes included in the top 1, top 10, and top 100 predicted target genes, respectively. PDGrapher outperforms the second-best method in all the recall metrics with the averaged margin being 3.24% (Chemical-PPI-Lung-MCF7), 3.18% (Chemical-PPI-Lung-A549), 11.77% (Chemical-PPI-Breast-MDAMB231), 8.33% (Chemical-PPI-Breast-BT20), 3.64% (Chemical-PPI-Prostate-PC3), 9.44% (Chemical-PPI-Prostate-VCAP), 0.3% (Chemical-PPI-Colon-HT29), 2.92% (Chemical-PPI-Skin-A375), and 5.05% (Chemical-PPI-Cervix-HELA) (Figure 3C). We then consolidated the results using the rankings from experiments across different cell lines and metrics for each method. PDGrapher achieved the best overall rankings, with a median significantly higher than all the baselines. (Figure 3D)

PDGrapher not only provides accurate predictions for a larger proportion of samples and consistently predicts ground-truth therapeutic targets close to the top of the ranked list, but it also predicts gene targets that are closer in the network to ground-truth targets compared to what would be expected by chance (Figure 3E). In all cell lines, the ground-truth therapeutic targets predicted by PDGrapher are significantly closer to the ground-truth targets compared to what would be expected by chance (Table S6). For example, for Chemical-PPI-Lung-A549, the median distance between predicted and ground-truth therapeutic targets is 3.0 for both PDGrapher and the Random baseline. However, the distributions exhibit a statistically significant difference, with a one-sided Mann-Whitney U test yielding a p-value < 0.001, an effect size (rank-biserial correlation) of 0.3531 (95% CI [0.3515, 0.3549]), and a U statistic of 1.29e11. Similarly, for Chemical-PPI-Breast-MCF7, the median distance is 3.0 for both groups, yet the distributions are significantly different (p-value < 0.001, effect size = 0.2160 (95% CI [0.2145, 0.2173]), U statistic = 3.91e11)(Table S6). This finding is significant as it is supported by the local network hypothesis, which posits that genes in closer network proximity tend to exhibit greater similarity compared to those that are more distantly connected [33–35]. This result implies that PDGrapher not only identifies relevant gene targets but does so in a manner that reflects the underlying biological and network-based relationships [28], suggesting that its predictions are not random but are rooted in the inherent structure of the gene interaction network.

It is noted that frameworks that train individual models for each perturbagen (scGEN and CellOT) generally perform better in perturbagen prediction than those that train a single model for all perturbagens (Biolord, GEARS and ChemCPA). However, the former approach is exhaustively time-consuming when applied to large-scale datasets with numerous perturbagens. For example, without parallel training, scGEN would require approximately 8 years to complete the leave-cell-out experiments for both chemical and genetic perturbation data used in this study, which is unacceptable. Training process of PDGrapher is up to 25 times faster than scGEN and more than 100 times faster than CellOT if it is trained using the default setting of 100,000 epochs, significantly reducing the computational cost of predicting perturbagens and highlighting another advantage of PDGrapher over these methods. The enhanced efficiency of PDGrapher is attributed to its unique approach. Existing approaches are predominantly perturbation response methods that predict changes in the phenotype that occur upon perturbation. Thus, they can identify perturbagens only indirectly by exhaustively predicting responses to all perturbations in the library and then searching for the perturbagen with the desired response that reverses a disease state. Unlike methods that learn responses to perturbations, PDGrapher addresses the inverse problem, which is to infer the perturbagen necessary to achieve a specific response – i.e., directly predicting perturbagens by learning which perturbations elicit a desired response.

PDGrapher also exhibits superior performance across genetic datasets, specifically Genetic-PPI-Lung-A549, Genetic-PPI-Breast-MCF7, Genetic-PPI-Prostate-PC3, Genetic-PPI-Skin-A375, Genetic-PPI-Colon-HT29, Genetic-PPI-Ovary-ES2, Genetic-PPI-Head-BICR6, Genetic-PPI-Pancreas-YAPC, genetic-PPI-stomach-AGS, and Genetic-PPI-Brain-U251MG (Figure S3). Briefly, PDGrapher successfully detected real targets in 0.87% (Genetic-PPI-Lung-A549), 0.52% (Genetic-PPI-Breast-MCF7), 0.24% (Genetic-PPI-Prostate-PC3), 0.38% (Genetic-PPI-Skin-A375), 0.36% (Genetic-PPI-Colon-HT29), 1.09% (Genetic-PPI-Ovary-ES2), 0.54% (Genetic-PPI-Head-BICR6), 0.11% (Genetic-PPI-Pancreas-YAPC), and 0.92% (Genetic-PPI-Brain-U251MG) more samples compared to the second-best baseline (Figure S3A). Its excellent performance to predict targets from the top of the ranks are further demonstrated by metrics recall@1 and recall@10 (Figure S3C). We observed that the performance of PDGrapher and the competing methods on genetic data are comparatively modest than those on chemical data. This relative modesty could stem from the inherent characteristics of knockout interventions, which often produce a reduced phenotypic signal compared to small molecule interventions. Studies have shown that while gene knockouts are essential for understanding gene function, single-gene knockout studies sometimes offer limited insights into complex cellular processes due to compensatory mechanisms [36–38]. Despite the modest performance in genetic intervention datasets, PDGrapher outperforms competing methods in the combinatorial prediction of therapeutic targets. We also observed superior performance of PDGrapher in response prediction for both chemical (Figure S1) and genetic data (Figure S3E-G).

When using GRNs as proxy causal graphs, we see that PDGrapher has comparable performance with GRN and PPI across both chemical and genetic intervention datasets (Supplementary Figures S7 and S8). One difference is that gene regulatory networks were constructed individually for each cell line, which makes leave-cell-out setting prediction particularly challenging. Therefore, we only conducted within-cell line experiments for the GRNs-based datasets.

We also use PDGrapher to illuminate the mode of action of chemical perturbagens Vorinostat and Sorafenib in Chemical-PPI-Lung-A549. See the results in Supplementary Note 1.

### PDGrapher generalizes to new (previously unseen) cell lines and learns genetic and chemical perturbagens.

**Figure 4:**
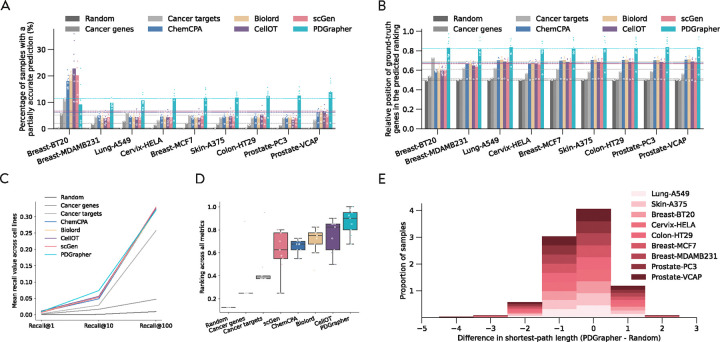
PDGrapher generalizes to new (previously unseen) cell lines and learns optimal chemical perturbagens in held out folds that contain both novel cell lines and novel samples. **(A)** PDGrapher consistently predicts ground-truth gene targets at the top of its ranked list of genes with up to 8.27% (Chemical-PPI-PC3: 12.55% vs 4.27%) more samples in the test set compared to the second-best baseline across Chemical-PPI datasets. **(B)** PDGrapher ranks ground-truth targets up to 15.18% (Chemical-PPI-MDAMB231: 81.81% vs 66.63%) higher than the second-best baseline in the predicted list across Chemical-PPI datasets **(C)** PDGrapher recovers ground-truth therapeutic targets at higher rates compared to competing methods for Chemical-PPI datasets. **(D)** The boxplots show the ranks across the experiments of different cell lines and metrics for each method. Higher value indicates better performance. The central line inside the box represents the median, while the top and bottom edges correspond to the first (Q1) and third (Q3) quartiles. The whiskers extend to the smallest and largest values within 1.5 times the interquartile range (IQR) from the quartiles. Each individual dot represents a data point for a specific cell line and metrics. **(E)** Shown is the difference of shortest-path distances between ground-truth therapeutic genes and predicted genes by PDGrapher and a Random baseline, with negative values indicating that PDGrapher predicts sets of therapeutic genes that are closer in the network to ground-truth therapeutic genes compared to what would be expected by chance [average shortest-path distances across cell lines for PDGrapher vs Random = 2.75 vs 3.11].

We observe consistently strong performance of PDGrapher across chemical intervention datasets in the leave-cell-out setting (Figure 4). In this setting, we use the trained models in the random-splitting setting for each cell line to predict therapeutic targets in the remaining cell lines. PDGrapher successfully predicts perturbagens that describe the cellular dynamics and shift gene expression phenotypes from a diseased to a treated state with 6.45% (Chemical-PPI-Lung-MCF7), 4.54% (Chemical-PPI-Lung-A549), 4.86% (Chemical-PPI-Breast-MDAMB231), 8.27% (Chemical-PPI-Prostate-PC3), 7.07% (Chemical-PPI-Prostate-VCAP), 6.94% (Chemical-PPI-Skin-A375), 7.09% (Chemical-PPI-Colon-HT29), and 6.86% (Chemical-PPI-Cervix-HELA) additional testing samples compared to the second-best baseline (Figure 4A). PDGrapher also outperforms the second-best method in nine cell lines by ranking the ground-truth targets 11.34% (Chemical-PPI-Lung-MCF7), 12.99% (Chemical-PPI-Lung-A549), 15.18% (Chemical-PPI-Breast-MDAMB231), 11.05% (Chemical-PPI-Breast-BT20), 13.08% (Chemical-PPI-Prostate-PC3), 12.82% (Chemical-PPI-Prostate-VCAP), 11.95% (Chemical-PPI-Skin-A375), 11.74% (Chemical-PPI-Colon-HT29), and 13.97% (Chemical-PPI-Cervix-HELA) higher in the predicted list (Figure 4B). Considering the overall performance across different cell lines and metrics, PDGrapher achieves the highest ranks, with a median surpassing all the baselines (Figure 4D). Additionally, combinations of therapeutic targets predicted by PDGrapher in chemical datasets are closer to ground-truth targets than expected by chance (Figure 4E, Table S7). For example, for Chemical-PPI-Lung-A549, the median distance between predicted and ground-truth therapeutic targets is 3.0 for both PDGrapher and the Random baseline. However, the distributions exhibit a statistically significant difference, with a one-sided Mann-Whitney U test yielding a p-value < 0.001, an effect size (rank-biserial correlation) of 0.2191 (95% CI [0.2182, 0.2200]), and a U statistic of 2.46e12. Similarly, for Chemical-PPI-Breast-MCF7, the median distance is 3.0 for both groups, yet the distributions are significantly different (p-value < 0.001, effect size = 0.2457 (95% CI [0.2450, 0.2464]), U statistic = 6.07e12) (Table S7). Besides chemical perturbations, PDGrapher outperforms existing methods in perturbagen prediction for genetic interventions across cell lines when considering the top targets on the predicted ranks (Figure S4A-D). PDGrapher also demonstrates superior performance in response prediction for both chemical (Figure S2) and genetic datasets (Figure S4E-G).

### PDGrapher predicts therapeutic targets for multiple cancer types.

**Figure 5:**
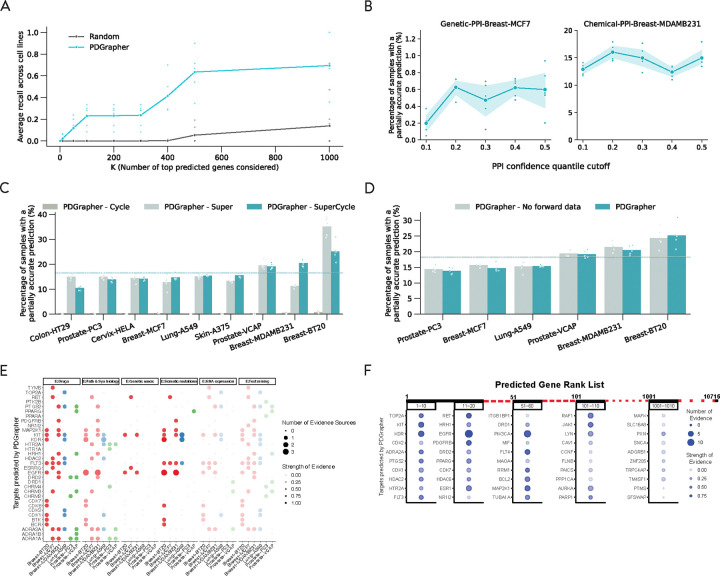
PDGrapher’s predictions illuminate mode of action of perturbagens. **(A)** Performance of PDGrapher to detect unseen approved drug targets in all cell lines with chemical perturbation evaluated by recall with K from 0 to 1000. K represents the number of predicted target genes considered in the predicted ranked list. **(B)** Performance of sensitivity analyses evaluated by partial accuracy for cell line MDAMB231 and MCF7 with chemical and genetic perturbation, respectively. The PPI used here is from STRING (string-db.org) with a confidence score for each edge. The edges are filtered by the 0.1, 0.2, 0.3, 0.4, and 0.5 quantiles of the confidence scores as cutoffs, resulting in five PPI networks with 625,818, 582,305, 516,683, 443,051, and 296,451 edges, respectively. **(C)** Performance metrics of ablation study on PDGrapher’s objective function components: PDGrapher-Cycle trained using only the cycle loss, PDGrapher-SuperCycle trained using the supervision and cycle loss, and PDGrapher-Super trained using only the supervision loss, evaluated by partial accuracy. **(D)** Performance metrics of the second ablation study on PDGrapher’s input data: PDGrapher- No forward data using only backward data and PDGrapher using both forward and backward data. The forward and backward data are organized as (healthy, mutation, disease) and (diseased, drug, treated), respectively. **(E)** Union of top 10 targets predicted by PDGrapher in lung, breast, and prostate cancer. The color intensity and size of the bubbles represent the number of evidence sources and the association scores for each type of evidence, respectively. Red, blue, and green dots stand for breast, lung, and prostate cancer, respectively. Symbol “E:” indicates evidence. See details of the scoring system in Supplementary Note 2. **(F)** Predicted targets from PDGrapher in five ranges, 1–10, 11–20, 51–60, 101–110, 501–510, and 1001–1010, on the predicted target list for lung cancer (A549). The color intensity and size of the bubbles represent the number of evidence sources and the global scores of targets from OpenTarget, respectively.

In addition to experiments with systematic and leave-cell-out splitting, we conducted experiments to examine PDGrapher ‘s ability to discover the targets of unseen approved drugs in training set for chemical cell lines with healthy data available (Chemical-PPI-Lung-A549, Chemical-PPI-Breast-MCF7, Chemical-PPI-Breast-MDAMB231, Chemical-PPI-Breast-BT20, Chemical-PPI-Prostate-PC3, Chemical-PPI-Prostate-VCAP). PDGrapher was used to predict gene targets to transition these diseased cell lines into their healthy states. Figure 5A shows the recovery of targets of FDA-approved drugs for varying values of K (K represents the number of predicted target genes considered in the predicted ranked list), indicating that PDGrapher can identify cancer drug targets among the top predictions.

We examined the Chemical-PPI-Breast-MCF7 dataset to evaluate PDGrapher for identifying drug targets for breast cancer. We excluded FDA-approved drugs from this evaluation in the training set to ensure an unbiased assessment of model performance. Using SAFE to visualize the PPI network used in training PDGrapher (Figure S5A), we highlight the targets of approved drugs (Supplementary Figure S5B) and compared them to the top K predicted targets by PDGrapher (Supplementary Figures S5C). There is a significant overlap between the spatial enrichment of the ground-truth gene targets of approved breast cancer drugs and the top K predicted gene targets by PDGrapher. This overlap strongly suggests that PDGrapher effectively predicts the targets that transition breast cancer cell states from diseased to healthy. Specifically, PDGrapher successfully identified CDK4 within the top 20 predictions and CDK6 within the top 100 predictions. Note that ribociclib and abemaciclib were absent from the training set of PDGrapher, underscoring PDGrapher ‘s robust capability to pinpoint therapeutic targets, even without prior exposure to known drug data.

We analyzed lung cancer by comparing the targets predicted by PDGrapher from lung cancer cell lines with the targets of candidate drugs currently in clinical development, curated from the Open Targets platform [39]. This allowed us to evaluate PDGrapher ‘s ability to predict combinatorial chemical perturbagens. Specifically, we compared the top 10 predicted targets for the A549 lung cancer cell line from PDGrapher with ten randomly selected genes. The results showed that the Open Targets scores and the number of supporting resources for the predicted targets were significantly higher than those for the random genes (Figure S15). Using a cutoff score of 0.5, 8 out of 10 predicted targets had evidence supporting their association with lung cancer, compared to only 2 out of 10 in the random set. Among these predicted targets, four drugs, tacedinaline (DrugBank ID: DB12291 and clinical trial ID: NCT00005093), selpercatinib (DrugBank ID: DB15685), pralsetinib (DrugBank ID: DB15822), and dexmedetomidine (DrugBank ID: DB00633 and [40]), were not included in the training set but have been identified as potential treatments for non-small cell lung cancer.

We then evaluated PDGrapher ‘s predictions by examining FDA-approved drugs that were not present in the training set of PDGrapher. Specifically, we assessed PDGrapher ‘s performance using the Chemical-PPI-Lung-A549 dataset, focusing initially on pralsetinib, a targeted cancer therapy primarily used to treat non-small cell lung cancer (NSCLC) [41]. Pralsetinib is a selective RET kinase inhibitor designed to block the activity of RET proteins that have become aberrantly active due to mutations or fusions. Pralsetinib is known to target 11 key proteins: RET, DDR1, NTRK3, FLT3, JAK1, JAK2, NTRK1, KDR, PDGFRB, FGFR1, and FGFR2 [42]. RET, the primary target of pralsetinib, was ranked 11th out of a total of 10,716 genes in the predicted list. Half of these targets (5 out of 11) were ranked within the top 100 predicted targets by PDGrapher, including KDR (ranked at 3), FLT3 (ranked at 10), RET (ranked at 11), PDGFRB (ranked at 14), and FGFR2 (ranked at 81). This substantial overlap highlights the potential of the candidate targets identified by PDGrapher for pralsetinib-based lung cancer treatment, particularly given that pralsetinib was not included in the training set and thus not encountered by PDGrapher during training.

Next, we examined EGFR as a therapeutic target for lung cancer. EGFR mutations are prevalent in NSCLC, particularly in adenocarcinoma (as modeled in A549 cells), and drive uncontrolled cell proliferation [43]. Targeted therapies, such as erlotinib, gefitinib, afatinib, osimertinib, and dacomitinib, are used to treat EGFR-mutant NSCLC [44]. PDGrapher identified EGFR among the top 20 predicted targets for Chemical-PPI-Lung-A549, despite having no prior knowledge of this target from the training set.

### PDGrapher predicts therapeutic targets and drugs for lung cancer.

Given that Open Targets offers more comprehensive evidence for targets currently under development, we conducted a second series of case studies utilizing Open Targets data to evaluate PDGrapher capability in identifying candidate therapeutic targets and drugs. This analysis aims to identify targets uniquely identified for lung cancer. Figure 5E presents a bubble plot illustrating the union of the top 10 predicted targets to transition cell states from diseased to healthy across the six cell lines from three cancer types that have healthy controls available. In the plot, the color intensity and size of the bubbles represent the number of evidence sources and the association scores for each type of evidence, respectively. Most predicted targets are supported by drugs, pathology and systemic biology, and somatic mutation databases, which were considered strong evidence sources. Two unique targets, TOP2A and CDK2, are predicted exclusively for the A549 lung cancer cell line (Figure S16AB). TOP2A is ranked as the top predicted target by PDGrapher. This gene encodes a crucial decatenating enzyme that alters DNA topology by binding to two double-stranded DNA molecules, introducing a double-stranded break, passing the intact strand through the break, and disciplining the broken strand. This mechanism is vital for DNA replication and repair processes. TOP2A might be a potential therapeutic target for anti-metastatic therapy of non-small cell lung cancer since it promotes the metastasis of NSCLC by stimulating the canonical Wnt signaling pathway and inducing EMT [45]. Leveraging TOP2A predicted target, PDGrapher then identified three drugs, aldoxorubicin, vosaroxin, and doxorubicin hydrochloride as candidate drugs. These drugs were not part of the training dataset of PDGrapher and are in the early stages of clinical development: aldoxorubicin and vosaroxin are in Phase II trials (ClinicalTrials.gov); doxorubicin hydrochloride is in Phase I but has been shown to improve the survival of patients with metastatic or surgically unresectable uterine or soft-tissue leiomyosarcoma [46].

Given that PDGrapher can rank all genes based on PPI or GRN data, we aimed to assess two key questions: (1) whether the genes ranked at the top have more substantial evidence from Open Targets compared to those ranked lower, and (2) what rank threshold should be considered to identify reliably predicted genes. Figure 5F illustrates the number of evidence sources and the global score for predicted target genes within the rank ranges of 1–10, 11–20, 51–60, 101–110, 501–510, and 1001–1010 for lung cancer (Chemical-PPI-Lung-A549). Our analysis revealed a clear trend: both the number of supporting evidence sources and the global scores decrease as the rank increases, further validating the predictive power of PDGrapher. We observed that most targets ranked within the top 100 show strong evidence from Open Targets, suggesting that a rank threshold of 100 could serve as a cutoff for selecting candidate targets.

### Training PDGrapher’s casually-inspired neural network models.

We perform an ablation study to analyze components in PDGrapher’s objective function across the chemical datasets. We train PDGrapher using only the cycle loss (PDGrapher-Cycle), only the supervision loss (PDGrapher-Super), and both (PDGrapher-SuperCycle) in the random splitting setting on all nine PPI chemical datasets. We evaluate the relative position of ground-truth therapeutic targets in the proportion of samples with the partially accurate prediction (Figure 5C), predicted gene ranking values, and recall values (Figure S11). We find that PDGrapher-Super has the highest performance when predicting correct perturbagens; however, it has the lowest performance in the reconstruction of treated samples. PDGrapher-SuperCycle (our choice throughout this study) has comparative performance when predicting correct perturbagens and the highest performance in the reconstruction of treated samples. Conversely, PDGrapher-Cycle performs poorly in identifying correct perturbagens and performs better when asked to predict (reconstruct) held-out treated samples. PDGrapher-SuperCycle appears as the best compromise between accuracy in predicting therapeutic genes and reconstruction of treated samples from diseased samples upon intervening on the predicted genes. The findings demonstrate that the supervision loss is crucial to PDGrapher ‘s overall performance. The PDGrapher- cycle model consistently underperforms across all cell lines and metrics. While PDGrapher- super often excels in ranking performance, including cycle loss (in PDGrapher- SuperCycle) proves its value by significantly improving top prediction metrics such as recall@1 and recall@10. PDGrapher- SuperCycle outperforms PDGrapher- Super in these top prediction metrics in more than half of the cell lines (5/9). We chose PDGrapher-SuperCycle throughout this work because we seek a model that provides: 1) accurate target gene predictions from the top-ranked genes in the predicted list; and 2) phenotypic-driven gene target predictions such that even incorrect predictions are guided by the changes they would generate in diseased samples.

Acknowledging the critical role of biological pathways in cellular functions, it becomes feasible for PDGrapher-SuperCycle to identify alternative gene targets within these pathways that may elicit similar phenotypic responses. The organization of genes in biological pathways, where each gene contributes to a specific biochemical process or signaling cascade, allows for the possibility that perturbations in different genes could lead to similar outcomes [47]. This pathway-based interconnectivity suggests that targeting different genes within a pathway may still achieve the desired therapeutic state, as these genes collectively influence certain cellular functions or phenotypes [48]. Consequently, while PDGrapher-SuperCycle may exhibit lower accuracy compared to PDGrapher-Super in pinpointing the precise original targets due to the interconnected and complex nature of biological pathways [49], it demonstrates enhanced efficacy in identifying a broader range of gene targets that can effectively transition cell states from diseased to treated conditions. This inductive bias aligns with the intricate organization of genes within biological pathways. It offers valuable insights into the diverse mechanisms of action of perturbagens, as substantiated by the results shown in the previous subsection. Such an approach underscores the potential of leveraging the organization of biological pathways to develop more comprehensive therapeutic interventions.

We conducted sensitivity analyses for PDGrapher using five PPIs built with varying edge confidence cutoffs. In these analyses, PDGrapher was trained using PPI data from STRING (https://string-db.org/) [50], which provides a confidence score for each edge. We filtered the edges using the 0.1, 0.2, 0.3, 0.4, and 0.5 quantiles of the confidence scores as cutoffs, resulting in five PPIs with varying numbers of edges. Results demonstrate robust performance across varying degrees of PPI confidence (Figure 5B). We selected two cell lines for the sensitivity analyses: Chemical-PPI-Breast-MDAMB231 and Genetic-PPI-Breast-MCF7. We processed the LINCS perturbation data for each cell line using the five PPI networks (see details in the method section). We ran PDGrapher with 1, 2, and 3 GNN layers, selecting the best configuration based on the performance of the validation set. As shown in Figure S12, PDGrapher maintains stable performance even as the number of missing edges in the PPI network increases across both chemical and genetic perturbation data. Notably, we did not observe any significant decline in performance as edges were removed from the PPIs, underscoring PDGrapher’s robust ability to handle incomplete PPI data during both training and inference. Similarly, we built two synthetic datasets with varying ratios of missing components and increasing intensity of latent confounder bias. Specifically, we introduced latent confounder bias into the gene expression data and imposed missing components on the PPIs (details of this data generation process are provided in the Methods section). PDGrapher also demonstrated stable performance in perturbagen prediction, with only a slight, non-significant drop in performance as more missing components or confounders were introduced (Figure S13 and S14).

Some cell lines might not have associated healthy control samples from disease-relevant tissues and cell types. These cell lines only have the treatment intervention data (diseased cell state, drug, treated cell state) without disease intervention data (healthy cell state, disease mutations, diseased cell state) in model training and inference. Response prediction and perturbagen discovery modules in PDGrapher can be trained using treatment intervention data only. For the cell lines with healthy control, we trained the response prediction module using both intervention data sources, while for the cell lines without healthy control, we trained it using only disease intervention data. To test PDGrapher when the model does not have access to healthy control data, we run PDGrapher with and without disease intervention data for six chemical cell lines (Chemical-PPI-Lung-A549, Chemical-PPI-Breast-MCF7, Chemical-PPI-Breast-MDAMB231, Chemical-PPI-Breast-BT20, Chemical-PPI-Prostate-PC3, Chemical-PPI-Prostate-VCAP) and three genetic cell lines (Genetic-PPI-Lung-A549, Genetic-PPI-Breast-MCF7, and Genetic-PPI-Prostate-PC3) that have healthy data available. Results show that the two versions of PDGrapher have very consistent performance over different cell types and data types (chemical and genetic) (Figure 5D and Figure S9 and S10). In about half of the cell lines (4/9), the model trained without disease intervention data outperforms the model trained with this data, indicating the weak dependency of PDGrapher on the healthy control data, suggesting that in the absence of healthy control data, PDGrapher can still perform well.

## Discussion

We introduce a novel problem formulation for phenotype-driven lead discovery. Given a diseased sample, the goal is to find genes that a genetic or chemical perturbagen should target to reverse disease effects and shift the sample to a treated state distributionally equivalent to a healthy state. In practice, this problem translates to predicting a combination of gene targets; therefore, we refer to this formulation of phenotype-driven lead discovery as a combinatorial prediction of therapeutic targets. To address this problem, we introduce PDGrapher. Given a diseased cell state represented as a gene expression signature and a proxy causal graph of gene-gene interactions, PDGrapher predicts candidate target genes to shift the cells to a desired treated state.

PDGrapher predicts perturbagens that shift cell line gene expression from a diseased to a treated state across two evaluation settings and eight datasets involving genetic and chemical interventions. Training PDGrapher models is faster than alternative response prediction methods, which rely on indirect prediction to identify candidate perturbagens. Instead of optimizing gene targets for existing compounds, PDGrapher aims to discover new therapeutic leads by optimizing gene targets to shift cells from diseased to treated states. This distinction sets it apart from other drug target prediction approaches [34, 35, 51–53].

The potential of PDGrapher is in enhancing the design of therapeutic leads and broadening the search space of perturbagens to include those above and beyond existing chemical and genetic libraries. PDGrapher effectively leverages large datasets of genetic and chemical interventions to find perturbagens as sets of candidate targets to shift cell line gene expressions from diseased to treated states. By flexibly selecting sets of therapeutic targets for intervention rather than on a specific perturbagen, PDGrapher enhances phenotype-driven lead discovery. PDGrapher’s approach in identifying therapeutic targets holds promise for personalized therapies, as it can enable tailoring treatments based on individual gene expression profiles, thereby addressing disease representations in each patient. PDGrapher’s capacity to simultaneously output multiple genes is highly relevant for diseases where dependencies involving multiple genes influence treatment efficacy and safety.

PDGrapher is developed under the assumption of no unobserved confounders. This assumption is stringent and difficult to verify empirically. Fruitful future directions include reevaluating and relaxing this assumption in the problem formulation, thereby refining the problem definition. Another limitation is the reliance on PPIs or GRNs as proxies for causal gene networks, as these networks are noisy and incomplete [54–56]. PDGrapher posits that leveraging representation learning can overcome incomplete causal graph approximations. A valuable research direction is to theoretically examine the impact of such approximations, focusing on how they influence the accuracy and reliability of predicted likelihoods. Such analyses could discover high-level causal variables with therapeutic effects from low-level observations and contribute towards reconciling structural causality and representation learning approaches, which are typically void of any causal understanding [57]. We performed two experiments to evaluate PDGrapher’s robustness. First, we tested PDGrapher on a PPI network with weighted edges, systematically removing increasing proportions of edges to assess its performance under data degradation. Second, we applied PDGrapher to synthetic datasets with varying levels of missing components in the graph and confounding factors in the gene expression data. In both experiments, PDGrapher consistently maintained stable performance.

There are limitations for phenotype-driven drug discovery that lie ahead of PDGrapher. One limitation is its reliance on transcriptomic data. While this offers broad applicability, other data modalities and experimental platforms, such as cell painting, can be valuable for phenotype-driven drug discovery. Cell painting can enhance phenotype-driven drug discovery by visually capturing cellular responses through staining organelles and cytoskeletal components [58,59]. This platform provides high-content image profiles, yielding signatures of cell changes that reveal nuanced effects of compounds. With the recent release of the JUMP Cell Painting dataset [60], this approach has the potential to synergize with databases like CMap and LINCS, providing a rich, image-based layer of phenotypic data that complements transcriptomic profiles. Integrating multimodal multi-omic datasets could offer a more comprehensive view of compound effects [61] and drastically expand the scope of PDGrapher [62, 63].

## Online Methods

### Datasets

We compiled and processed six primary data sources and two additional repositories of biological information. Data sources include protein-protein interactions (PPI), healthy and diseased cell line gene expression, diseased cell line gene expression upon chemical or genetic interventions, phenotype-associated genes, drug targets, and drug indications. The following is a description of the data sources and preprocessing steps.

#### Human protein-protein interaction network.

We built a PPI network by aggregating proteins and connections from BIOGRID [64] (accessed in March 2022), HuRI [65], and Menche et al. [48] In this graph, nodes represent human proteins, and edges exist between nodes if there is physical interaction between the proteins. We downloaded a gene ID mapping file from the HUGO Gene Nomenclature Committee. Using this file, we mapped proteins in BIOGRID and Menche et al. [48] from Entrez Gene ID [66] to HUGO Gene Nomenclature Committee ID [67], and proteins in HuRI from Ensembl Gene ID [68] to HUGO Gene Nomenclature Committee ID [67]. Our final PPI comprises the union of nodes and edges, resulting in a graph with 15,742 nodes and 222,498 undirected edges.

#### Gene expression data.

We downloaded Library of Integrated Network-Based Cellular Signatures (LINCS [69]) level 3 gene expression data from https://clue.io/releases/data-dashboard (accessed in February 2022). Level 3 data consists of quantile-normalized samples across each plate and is appropriate for cross-plate analyses. LINCS contains gene expression measurements for 12,327 genes upon genetic and chemical interventions. There are 387,317 samples upon CRISPR genetic interventions (treated samples), with 5,156 unique knocked-out genes across 27 unique cell lines. There is an average of 17.18 replicates per cell line-knocked-out gene pair. The number of unique genes knocked out in each cell line varies from 1 to 5,114, with an average of 2,042.14 unique genes knocked out per cell line.

Control data for CRISPR interventions, that is, diseased samples, are genetic interventions that either do not contain a gene-specific sequence or whose gene-specific sequence targets a gene not expressed in the human genome. There are 47,781 diseased samples across 50 cell lines. The number of diseased samples for each cell line varies from 1 to 6,890, with an average of 955.62 diseased samples per cell line.

There are 1,313,292 samples upon chemical interventions (treated samples), with 31,234 unique compounds across 229 unique cell lines. There is an average of 7.96 replicates per cell line-compound pair. The number of compounds tested in each cell line varies from 1 to 19,509, with an average of 719.69 unique compounds tested per cell line. Drugs are administered at different doses and measured at varying time points after treatment. On average, there are 2.73 different doses per compound-cell line pair, with a minimum of 1 and a maximum of 26 different doses. On average, gene expression is measured at 1.25 time points per compound-cell line pair, with a minimum of 1 and a maximum of 13 different time points.

Control data for chemical interventions, that is, diseased samples, are treated with a vehicle (dimethyl sulfoxide). There are 76,795 diseased samples across 226 cell lines. The number of diseased samples for each cell line varies from 1 to 7,336, with an average of 339.80 diseased samples per cell line. On average, gene expression of diseased samples is measured at 1.4 time points, with a minimum of 1 and a maximum of 5 different time points.

We filter cell lines to keep those treated with at least 4,000 unique genetic or chemical perturbagens, resulting in 10 selected cell lines for each genetic and chemical dataset. To find healthy cell line counterparts, we extracted all cell lines with the “Unknown” tumor phase in the downloaded LINCS dataset (N=145). Then, we filtered the cell lines by tissue type. To find the exact match to diseased cell lines, we performed a manual literature search to confirm their experimental use as healthy counterparts. We extracted healthy counterparts for three of the ten diseased cell lines: cell line NL20 as the healthy counterpart for A549, cell line MCF10A as the healthy counterpart for MCF7, and cell line RWPE1 as the healthy counterpart for PC3.

Genetic interventions correspond to gene experiment knockouts in which the gene expression of the knocked-out gene after the intervention is zero. Chemical interventions correspond to small molecule treatments, each targeting one or more proteins. Chemical interventions were performed at different dose levels and measured at different time points. We included replicates measured at all time points and doses. For each cell line and condition (healthy, diseased, and treated), we log-normalized level 3 gene expression data. We applied a min-max normalization to transform gene expression values into the range [0, 1] following established practices in the field.

We match genes in LINCS to proteins in our PPI using the HUGO Gene Nomenclature Committee ID [67], resulting in 10,716 overlapping genes and 151,839 undirected edges. Furthermore, we excluded treated samples from our datasets whose targeted genes were not included in the PPI.

We have healthy, diseased, and treated gene expression samples for each cell line treated with several genetic or chemical perturbagens (Table 1 and 2). For healthy counterparts, samples with the corresponding treatment (“vector” for genetic perturbagens, and “vehicle” for chemical perturbagens) are not available, therefore, we use the closest possible one (see “Sample category” in Table 1 and 2.

#### Gene regulatory networks.

We computed one gene regulatory network (GRN) for each diseased cell line in each condition (genetic and chemical datasets), using the GENIE3 [70] algorithm on gene expression values of each diseased cell line. We filtered genes in our gene expression dataset (LINCS) to contain only those in the PPI before running the GRN algorithm for consistency between the PPI and GRNs. GENIE3, introduced in 2010, won the Dialogue for Reverse Engineering Assessments and Methods 4 (DREAM4) challenge [71], which evaluates the success of GRN inference algorithms on benchmarks of simulated data. GENIE3 was introduced in the open source software for bioinformatics Bioconductor [72] in 2017 and is still used as a gold-standard for GRN generation [73–76]. It is a model based on an ensemble of regression trees and requires as input a matrix of gene expression levels under various conditions. Notably, this expression data is multifactorial. This means that they represent expression levels resulting from a perturbation over a set of genes rather than from a targeted experiment. Multifactorial expression can be obtained as samples from different patients or other biological systems. Therefore, cell line diseased samples are the closest to the ideal input data for GENIE3. GENIE3 produces a directed graph representing gene-gene regulatory interactions. This is achieved by assigning weights to regulatory links and maximizing weights for more significant links. Then, a significance threshold is used to determine which links are substantial enough to be predicted as a regulatory link. We adopted the threshold to generate GRNs with close network density as the PPI from STRING, which was achieved by keeping about 500 thousands directed edges.

#### Disease-associated genes.

We extracted disease-associated genes from COSMIC [77] (Accessed in October 2022) in addition to expert-curated genes available at https://cancer.sanger.ac.uk/cosmic/curation. Genes were represented using the HUGO Gene Nomenclature Committee ID. For each cell line in our dataset that has disease intervention data (see Disease intervention data header), we extracted cancer-causing mutations as the list of genes with “Verified” *Mutation verification status* in COSMIC and present in the list of genes curated by experts. Mapping the resulting genes to our list of genes in the PPI resulted in eight disease-associated genes for lung cancer cell line A549, nine disease-associated genes for breast cancer cell line MCF7, one disease-associated gene for prostate cancer cell line PC3, two disease-associated genes for prostate cancer cell line VCAP, six disease-associated genes for breast cancer cell line MDAMB231, and eight disease-associated genes for breast cancer cell line BT20.

#### Drug targets.

We downloaded drug-related data from DrugBank [78] (accessed in November 2022). We extracted drug names and synonyms, chemical identifiers, drug-gene targets, and all available synonyms for each gene target. Only the nominal targets, genes that produce proteins to which the drug physically interacts, were considered when processing the DrugBank data. Genes or gene products involved in a drug’s mechanism of action (MoA) were excluded and not retained for further analysis. We mapped drugs in DrugBank with chemical perturbagens in LINCS by InChI Key [79], resulting in 1,522 out of 31,234 unique LINCS compounds mapped to DrugBank with information of at least one target. We mapped drug targets to our PPI network using the HUGO Gene Nomenclature Committee ID, excluding any drug target that was not mapped. Chemical interventions target multiple genes, with a minimum of 1, a maximum of 300, and an average of 2.44 targets per compound.

#### Cancer drug and target information.

We extracted the list of cancer drugs by cancer type from NCI (https://www.cancer.gov/about-cancer/treatment/types/targeted-therapies/approved-drug-list; Accessed in July 2024). We mapped drug names to DrugBank to obtain cancer drug-gene targets. In total, there are 24 drugs associated with breast cancer (cell lines MCF7, MDAMB231, and BT20), 30 drugs associated with lung cancer (cell line A549), 11 drugs associated with prostate cancer (cell lines PC3 and VCAP), 13 drugs associated with colon cancer (cell line HT29), 18 drugs associated with skin cancer (cell line A375), three drugs associated with cervix cancer (cell line HELA), five drugs associated with ovary cancer (cell line ES2), four drugs associated with head and neck cancer (cell line BICR6), five drugs associated with pancreas cancer (cell line YAPC), five drugs associated with stomach cancer (cell line AGS), and six drugs associated with brain cancer (cell line U251MG).

#### Disease intervention data.

Disease intervention datasets consist of gene expression measurements of healthy cell lines, disease-associated genes, and gene expression measurements of diseased cell lines. Gene expression samples of healthy and diseased cell lines were retrieved from LINCS [69], and disease-associated genes were retrieved from COSMIC [77], as detailed previously. Each dataset $𝒯=\left\{T_{1},\ldots ,{\;T}_{M}\right\}$ is a collection of paired healthy-diseased cell lines where in each sample $T=<x^{h},𝒰,x^{d}>,x^{h}$ corresponds to gene expression values of the healthy cell line, set 𝒰 is comprised by a randomized subset of disease-associated genes, and $x^{d}$ corresponds to gene expression values of diseased cell lines (that is, upon mutations on genes in 𝒰). To select the randomized set of disease-associated genes, we first choose at random a proportion p∈{0.25,0.50,0.75,1}, and then select N disease-associated genes at random where N is the proportion multiplied by the total number of disease-associated genes. Given that more diseased samples are available than healthy samples (see Table 1 and 2) when building the triplets, we select a random sample from the set of healthy samples and, therefore, have non-unique healthy samples during training. In total, we built six datasets of disease interventions: the first three comprised of gene expression of healthy cell line MCF10A, breast cancer mutations, and gene expression of breast cancer cell line MCF7, MDAMB231, and BT20; the fourth comprised of gene expression of healthy cell line NL20, lung cancer mutations, and gene expression of lung cancer cell line A549; the fifth and sixth comprised of gene expression of healthy cell line RWPE1, prostate cancer mutations, and gene expression of prostate cancer cell line PC3 and VCAP. Find more details on data compilation and processing in previous subsections.

#### Treatment intervention data - genetic.

Genetic treatment intervention datasets consist of single-gene knockout experiments using CRISPR / Cas9-mediated gene knockout. Genetic treatment intervention data comprises gene expression measurements of diseased cell lines, single knocked-out genes, and gene expression measurements of treated cell lines. Gene expression samples of diseased and treated cell lines and knocked-out genes were retrieved from LINCS [69]. Each dataset $𝒯=\left\{T_{1},\ldots ,{\;T}_{M}\right\}$ is a collection of paired diseased-treated cell lines where in each sample $T=<x^{d},{𝒰}^{\prime },x^{t}>,x^{d}$ corresponds to gene expression values of the diseased cell line, set ${𝒰}^{\prime }$ is comprised by the knocked-out gene, and $x^{t}$ corresponds to gene expression values of treated cell lines (that is, upon knocking-out the gene in ${𝒰}^{\prime }$). Given that more treated samples are available than diseased samples (see Table 1) when building the triplets, we select a random sample from the set of diseased samples and, therefore, have non-unique diseased samples during training. In total, we built ten datasets of treatment interventions: A549, MCF7, PC3, A375, HT29, ES2, BICR6, YAPC, AGS, and U251MG. They are comprised of gene expression of diseased cells, knocked-out genes, and gene expression of treated cells. Find more details on data compilation and processing in previous subsections.

#### Treatment intervention data - chemical.

Chemical treatment intervention datasets consist of chemical compound treatment experiments. Chemical treatment intervention data comprises gene expression measurements of diseased cell lines, chemical compound therapeutic targets, and gene expression measurements of treated cell lines. Gene expression samples of diseased and treated cell lines were retrieved from LINCS, and chemical compound targets were retrieved from DrugBank, as detailed previously. Each dataset $𝒯=\left\{T_{1},\ldots ,{\;T}_{M}\right\}$ is a collection of paired diseased-treated cell lines where in each sample $T=<x^{d},{𝒰}^{\prime },x^{t}>,x^{d}$ corresponds to gene expression values of the diseased cell line, set ${𝒰}^{\prime }$ is comprised by the chemical compound targets, and $x^{t}$ correspond to gene expression values of treated cell lines (that is, upon treated with the chemical perturbagen targeting genes in ${𝒰}^{\prime }$). Given that more treated samples are available than diseased samples (see Table 2) when building the triplets, we select a random sample from the set of diseased samples and, therefore, have non-unique diseased samples during training. In total, we built nine datasets of treatment interventions: A549, MCF7, PC3, VCAP, MDAMB231, BT20, HT29, A375, and HELA. They comprised gene expression of diseased cells, chemical compound target genes, and gene expression of treated cells. Find more details on data compilation and processing in previous subsections.

### Related work

#### Learning optimal interventions.

The problem of learning interventions to achieve a desired state has gained interest in recent years. A few recent works formulate this problem as finding optimal interventions to optimize an associated outcome [80–83]. These works offer varied approaches. For example, Mueller et al. [80] aim to learn an intervention policy defined by a covariate transformation that produces the largest post-intervention improvement with high uncertainty. Pacchiano et al. [81] formalize the task as a bandit optimization problem in which each bandit’s arm corresponds to a covariate to intervene, and the goal is to recover an almost optimal arm in the least number of arm pulls possible. Mueller et al. [82] and Hie et al. [83] approach the problem of sequence-based data where each sequence is associated with an outcome, and the goal is to find mutations in the input sequence that increase a desired outcome. Other recent works formulate this problem as finding optimal interventions to shift the system to a desired state. Zhang et al. [84, 85] aimed to find an intervention that applied to a distribution helps match a desired distribution. Specifically, given a distribution P over X and a desired distribution Q over X, the goal is to find an optimal matching intervention I such that $P^{I}$ best matches Q under some metric. They address the special case of soft interventions (shift interventions) and use the expectation of distributions as the distance metric.

#### Neural networks and structural causal models (SCMs).

Causal representation learning has been a growing trend in recent years [86]. It aims to combine the strength of traditional causal learning methods with the robust capabilities of deep learning in the face of large and noisy data. Bottlenecks of traditional causal learning methods include unstructured high-dimensional variables, combinatorial optimization problems, unknown intervention, unobserved confounders, selection bias, and estimation bias [86]. There are three areas in which deep learning helps to overcome these bottlenecks [86]. First, in learning causal variables from high-dimensional unstructured data. Second, in learning the causal structure between causal variables, called *causal discovery* within the causal inference literature. Third, it facilitates the inference of interventional and counterfactual queries. Within the last branch, a promising approach aims to join SCMs and neural models to facilitate interventional and counterfactual querying. Parafita et al. put forward the requirements that any DL model should fulfill to approximate causal queries and introduced normalizing causal flows as a specific instantiation [87]. Pawlowski et al. followed a similar approach to introduce a model capable of computing counterfactual queries [88]. Xia et al. approached the problem differently, introducing a Neural Causal Model (NCM), a type of SCM with neural networks as structural equations [89]. Together with the NCM, they introduced an algorithm that provably performs identification and inference of interventional queries [89]. A follow-up work extended the NCM framework for identification and inference of counterfactual queries [90]. The concept of NCMs inspires our work by considering the graph in which we operate as a noisy version of a causal graph and our model operating on the graph as a proxy for the structural equations.

#### Interventions in graph neural networks (GNNs).

GNNs are a type of neural model that falls under the umbrella term of geometric deep learning [91–93]. These models use graph-structured data to compute transformed representations useful for downstream predictive tasks. Their ability to operate over graphs makes them especially relevant to NCMs. A recent work by Zecevic et al. [94] explored this connection. It introduced interventional GNNs, a GNN in which interventions are represented through mutilations in the input graph, and interventional inference as GNN computations on the mutilated graph [95]. We borrow this concept in our work and extend the representational capabilities of GNNs by assigning learnable embeddings to input nodes.

## Methods

### Preliminaries.

A calligraphic letter 𝒳 indicates a set, an italic uppercase letter X denotes a graph, uppercase X denotes a matrix, lowercase x denotes a vector, and a monospaced letter X indicates a tuple. Uppercase letter X indicates a random variable, and lowercase letter x indicates its corresponding value; bold uppercase X denotes a set of random variables, and lowercase letter x indicates its corresponding values. We denote P(X) as a probability distribution over a set of random variables X and P(X=x) as the probability of X is equal to the value of x under the distribution P(X). For simplicity, P(X=x) is abbreviated as P(x).

### Problem formulation - combinatorial prediction of therapeutic targets.

Intuitively, given a diseased cell line sample, we would like to predict the set of therapeutic genes that need to be targeted to reverse the effects of disease, that is, the genes that need to be perturbed to shift the cell gene expression state as close as possible to the healthy state. Next, we formalize our problem formulation. Let M=<E,V,ℱ,P(E)> be an SCM associated with causal graph G, where E is a set of exogenous variables affecting the system, V are the system variables, ℱ are structural equations encoding causal relations between variables and P(E) is a probability distribution over exogenous variables. Let $𝒯=\left\{T_{1},\ldots ,{\;T}_{m}\right\}$ be a dataset of paired healthy and diseased samples, where each element is a 3-tuple $T=<v^{h},U,v^{d}>$ with $v^{h}\in [0,\;1{]}^{N}$ being gene expression values of healthy cell line (variable states before perturbation), $V_{U}$ being the disease-causing perturbed variable (gene) set in V, and $v^{d}\in [0,\;1{]}^{N}$ being gene expression values of diseased cell line (variable states after perturbation). Our goal is to find, for each sample $T=<v^{h},U,v^{d}>$, the variable set $U^{\prime }$ with the highest likelihood of shifting variable states from diseased $v^{d}$ to healthy $v^{h}$ state. To increase generality, we refer to the desired variable states as *treated* $\left(v^{t}\right)$. Our goal can then be expressed as:

(1) $${argmax}_{U^{\prime }}P^{G^{U}}\left(V=v^{t}|do\left(U^{\prime }\right)\right),$$

where $P^{G^{U}}$ represents the probability over graph G mutilated upon perturbations in variables in U. Under the assumption of no unobserved confounders, the above interventional probability can be expressed as a conditional probability on the mutilated graph $G^{U^{\prime }}$:

(2) $${argmax}_{U^{\prime }}P^{G^{U^{\prime }}}\left(V=v^{t}|U^{\prime }\right),$$

which under the causal Markov condition is:

(3) $$argmax_{U^{\prime }}\prod_{i}P\left(V_{i}=v_{i}^{t}|Pa_{v_{i}}\right),$$

where ${Pa}_{V_{i}}$ represents parents of variable $V_{i}$ according to graph $G^{U^{\prime }}$ (that is, the mutilated graph upon intervening on variables in $U^{\prime }$). Here, state of a variable $V_{j}\in {\;Pa}_{V_{i}}$ will be equal to an arbitrary value $v_{j}^{\prime }$ if $V_{j}\in U^{\prime }$. Therefore, intervening on the variable set $U^{\prime }$ modifies the graph used to obtain conditional probabilities and determine the state of variables in $U^{\prime }$.

### Problem formulation - representation-learning-based combinatorial prediction of therapeutic targets.

In the previous section, we drew on the SCM framework to introduce a generic formulation for the task of combinatorial prediction of therapeutic targets. Instead of approaching the problem from a purely causal inference perspective, we draw upon representation learning to approximate the queries of interest to address the limiting real-world setting of a noisy and incomplete causal graph. Formulating our problem using the SCM framework allows for explicit modeling of interventions and formulation of interventional queries. Inspired by this principled problem formulation, we next introduce the problem formulation using a representation learning paradigm.

We let G=(𝒱,ℰ) denote a graph with |𝒱|=n nodes and |ℰ| edges, which contains partial information on causal relationships between nodes in 𝒱 and some noisy relationships. We refer to this graph as *proxy causal graph*. Let $𝒯=\left\{T_{1},\ldots ,{\;T}_{M}\right\}$ be a dataset with an individual sample being a 3-tuple $T=<x^{h},𝒰,x^{d}>$ with $x^{h}\in [0,\;1{]}^{n}$ being the node states (attributes) of healthy cell sample (before perturbation), 𝒰 being the set of disease-causing perturbed nodes in 𝒱, and $x^{d}\in [0,\;1{]}^{n}$ being the node states (attributes) of diseased cell sample (after perturbation). We denote by $G^{𝒰}=(𝒱,{ℰ}^{𝒰})$ the graph resulting from the mutilation of edges in G as a result of perturbing nodes in 𝒰 (one graph per perturbagen; we avoid using superindices for simplicity). Here again, we refer to the desired variable states as *treated* $\left(x^{t}\right)$. Our goal is then to learn a function:

(4) $$f\;:G^{{𝒰}^{\prime }},x^{d},x^{t}\to {argmax}_{{𝒰}^{\prime }}P^{G^{{𝒰}^{\prime }}}\left(x=x^{t}|x^{d},{𝒰}^{\prime }\right).$$

That is, given the graph $G^{{𝒰}^{\prime }}$, the diseased $x^{d}$ and treated $x^{t}$ node states, predicts the combinatorial set of nodes ${𝒰}^{\prime }$ that if perturbed have the highest chance of shifting the node states to the treated state $x^{t}$. We note here that $P^{G^{{𝒰}^{\prime }}}$ represents probabilities over graph $G^{𝒰}$ mutilated upon perturbations in nodes in ${𝒰}^{\prime }$. Under Causal Markov Condition, we can factorize $P^{G^{{𝒰}^{\prime }}}$ over graph $G^{{𝒰}^{\prime }}$:

(5) $$f\;:G^{{𝒰}^{\prime }},x^{d},x^{t}\to argmax_{{𝒰}^{\prime }}\prod_{i}P\left(x_{i}=x_{i}^{t}|x_{𝒫{𝒜}_{i}}\right),$$

that is, the probability of each node depending only on its parents $𝒫{𝒜}_{i}$ in graph $G^{{𝒰}^{\prime }}$.

We assume (i) real-valued node states, (ii) G is fixed and given, and (iii) atomic and non-atomic perturbagens (intervening on individual nodes or sets of nodes). Given that the value of each node should depend only on its parents on the graph $G^{{𝒰}^{\prime }}$, a message-passing framework appears especially suited to compute the factorized probabilities P.

In the SCM framework, the conditional probabilities in Equation 3 are computed recursively on the graph, each being an expectation over exogenous variables **E**. Therefore, node states of the previous time point are not necessary. To translate this query into the representation learning realm, we discard the existence of noise variables and directly try to learn a function encoding the transition from an initial state to a desired state. An exhaustive approach to solving Equation 5 would be to search the space of all potential sets of therapeutic targets ${𝒰}^{\prime }$ and score how effective each is in achieving the desired treated state. This is, indeed, how many cell response prediction approaches can be used for perturbagen discovery [21, 22, 96]. However, with moderately sized graphs, this is highly computationally expensive, if not intractable. Instead, we propose to search for potential perturbagens efficiently with a perturbagen discovery module $\left(f_{p}\right)$ and a way to score each potential perturbagen with a response prediction module $\left(f_{r}\right)$.

### Relationship to conventional graph prediction tasks.

Given that the prediction for each variable is dependent only on its parents in a graph, GNNs appear especially suited for this problem. We can formulate the query of interest under a graph representation learning paradigm as: Given a graph G=(𝒱,ℰ), and paired sets of node attributes $𝒳=\left\{X_{1},X_{2},\ldots ,X_{m}\right\}$ and node labels $𝒴=\left\{Y_{1},Y_{2},\ldots ,Y_{m}\right\}$ where each $Y=\left\{y_{1},\ldots ,y_{n}\right\}$, with $y_{i}\in [0,\;1]$, we aim at training a neural message passing architecture that given node attributes $X_{i}$ predicts the corresponding node labels $Y_{i}$. There are, however, some major differences between our problem formulation and the conventional graph prediction tasks, namely, graph and node classification (summarized in Table 3).

In node classification, a single graph G is paired with node attributes X, and the task is to predict the node labels Y. Our formulation differs in that we have m paired sets of node attributes 𝒳 and labels 𝒴 instead of a single set, yet they are similar in that there is a single graph in which GNNs operate. In graph classification, a set of graphs $𝒢=\left\{G_{1},\ldots ,G_{m}\right\}$ is paired with a set of node attributes $𝒳=\left\{X_{1},X_{2},\ldots ,X_{m}\right\}$ and the task is to predict a label for each graph $y=\left\{y_{1},\ldots ,y_{m}\right\}$. Here, graphs have a varying structure, and both the topological information and node attributes predict graph labels. In our formulation, a single graph is combined with each node attribute $X_{i}$, and the goal is to predict a label for each node, not for the whole graph.

### PDGrapher model.

PDGrapher is an approach for combinatorial prediction of therapeutic targets composed of two modules. First, a perturbagen discovery module $f_{p}$ searches the space of potential gene sets to predict a suitable candidate ${𝒰}^{\prime }$. Next, a response prediction module $f_{r}$ checks the goodness of the predicted set ${𝒰}^{\prime }$, that is, how effective intervening on variables in ${𝒰}^{\prime }$ is to shift node states to the desired treated state $x^{t}$.


(1) xd,xt→fp𝒰^′

(2) xd,𝒰^′→frxˆt.

### Model optimization.

We optimize our response prediction module $f_{r}$ using cross-entropy loss on known triplets $<x^{h},𝒰,x^{d}>$ and $<x^{d},{𝒰}^{\prime },x^{t}>$:

(6) $${ℒ}_{f_{r}}=CE\left(x^{d},f_{r}\left(x^{h},𝒰\right)\right)+CE\left(x^{t},f_{r}\left(x^{d},{𝒰}^{\prime }\right)\right).$$

We optimize our intervention discovery module $f_{p}$ using a cycle loss such that the response upon a predicted ${𝒰}^{\prime }$ is as close to the desired treated state as possible. In addition, we provide a supervisory signal for predicting ${𝒰}^{\prime }$ in the form of cross-entropy loss as follows:

(7) $${ℒ}_{f_{p}}=CE\left(x^{t},f_{r}\left(x^{d},f_{p}\left(x^{d},x^{t}\right)\right)\right)+CE\left({𝒰}^{\prime },f_{p}\left(x^{d},x^{t}\right)\right)\;\text{(with}f_{r}\;\text{frozen).}$$

We train $f_{p}$ and $f_{r}$ in parallel and implement early stopping separately (see *Experimental setup* for more details). Trained modules $f_{p}$ and $f_{r}$ are then used to predict, for each diseased cell sample, which nodes should be perturbed $\left({𝒰}^{\prime }\right)$ to achieve a desired treated state (Figure 1A).

### Response prediction module.

Our response prediction module $f_{r}$ should learn to map preperturbagen node values to post-perturbagen node values through learning relationships between connected nodes (equivalent to learning structural equations in SCMs) and propagating the effects of perturbations downstream in the graph (analogous to the recursive nature of query computations in SCMs).

Given a triplet $<x^{h},𝒰,x^{d}>$, we propose a neural model operating on a mutilated graph, $G^{𝒰}$ where the node attributes are the concatenation of $x^{h}$ and $x_{𝒰}^{\prime }$, predicting diseased node values $x^{d}$. Each node i has a two-dimensional attribute vector $d_{i}=\left[x_{i}^{h}\|x_{𝒰}^{\prime }\right]$, where the first element is its gene expression value $x_{i}^{h}$, and the second is a perturbation flag: a binary label indicating whether a perturbation occurs at node i. In practice, we embed each node feature into a high-dimensional continuous space by assigning learnable embeddings to each node based on the value of each input feature dimension. Specifically, for each node, we use the binary perturbation flag to assign a d-dimensional learnable embedding, which is different between nodes but shared across samples for each node. To embed the gene expression value $x_{i}^{h}\in [0,\;1]$, we first calculate thresholds using quantiles to assign the gene expression value into one of the B bins. We use the bin index to assign a d-dimensional learnable embedding, which is different between nodes but shared across samples for each node. To increase our model’s representation power, we concatenate a d-dimensional positional embedding (d-dimensional vector initialized randomly following a normal distribution). Concatenating these three embeddings results in an input node representation of dimensionality 3d. For each node i∈𝒱, an embedding $z_{i}$ is computed using a graph neural network operating on the node’s neighbors’ attributes. The most general formulation of a GNN layer is:

(8) $$h_{i}^{\prime }=\phi \left(h_{i},\underset{j\in {𝒩}^{i}}{⊕}\psi \left(h_{i},\;h_{j}\right)\right),$$

where $h_{i}^{\prime }$ represents the updated information of node i, and $h_{i}$ represents the information of node i in the previous layer, with embedded $d_{i}$ being the input to the first layer. ψ is a *MESSAGE* function, ⨁ an *AGGREGATE* function (permutation-invariant), and ϕ is an *UPDATE* function. We obtain an embedding $z_{i}$ for node i by stacking K GNN layers. Node embedding $z_{i}\in R$ is then passed to a multilayer feed-forward neural network to obtain an estimate of the post-perturbation node values $x^{d}$.

### Perturbation discovery module.

Our perturbagen prediction module $f_{p}$ should learn the nodes in the graph that should be perturbed to shift node states (attributes) from diseased $x^{d}$ to a desired treated state $x^{t}$.

Given a triplet $<x^{d},{𝒰}^{\prime },x^{t}>$, we propose a neural model operating on graph $G^{{𝒰}^{\prime }}$ with node features $x^{d}$ and $x^{t}$ that predicts a ranking for each node where the top P ranked nodes should be predicted as the nodes in ${𝒰}^{\prime }$. Each node i has a two-dimensional attribute vector: $d_{i}=\left[x_{i}^{d}\|x_{i}^{t}\right]$. In practice, we represent these binary features in a continuous space using the same approach as described for our response prediction module $f_{r}$.

For each node i∈𝒱, an embedding $z_{i}$ is computed using a graph neural network operating on the node’s neighbors’ attributes. We obtain an embedding $z_{i}$ for node i by stacking K GNN layers. Node embedding $z_{i}\in R$ is then passed to a multilayer feed-forward neural network to predict a real-valued number for node i.

### Network proximity between predicted and ground truth perturbagens.

Let 𝒫 be the set of predicted therapeutic targets, ℛ be the set of ground truth therapeutic targets, and spd(p,r) be the shortest-path distance between nodes in P and R. We measure the closest distance between P and R as:

(9) $$d\left(P,R\right)=\frac{1}{\left|R\right|\left|P\right|}\sum_{r\in R}\sum_{p\in P}spd\left(p,r\right).$$

### Model implementation and training.

We implement PDGrapher using PyTorch 1.10.1 [97] and the Torch Geometric 2.0.4 Library [98]. The implemented architecture yields a neural network with the following hyperparameters: number of GNN layers and number of prediction layers. We set the number of prediction layers to two and performed a grid search over the number of GNN layers (1–3 layers). We train our model using a 5-fold cross-validation strategy and report PDGrapher’s performance resulting from the best-performing hyperparameter setting.

### Further details on statistical analysis

We next outline the evaluation setup, baseline models, and statistical tests used to evaluate PDGrapher. We evaluate the performance of PDGrapher against a set of baselines:

- **Random baseline:** Given a sample $T=<x^{d},{𝒰}^{\prime },x^{t}>$, the random baseline returns N random genes as the prediction of genes in ${𝒰}^{\prime }$, where N is the number of genes in ${𝒰}^{\prime }$.
- **Cancer genes:** Given a sample $T=<x^{d},{𝒰}^{\prime },x^{t}>$, the cancer genes baseline returns the top N genes from an ordered list where the first M genes are disease-associated (cancer-driver genes). The remaining genes are ranked randomly, and N is the number of genes in ${𝒰}^{\prime }$. The processing of cancer genes is described in the section on Disease-associated genes.
- **Cancer drug targets:** Given a sample $T=<x^{d},{𝒰}^{\prime },x^{t}>$, the cancer genes baseline returns the top N genes from an ordered list where the first M genes are cancer drug targets and the remaining genes are ranked randomly, and N is the number of genes in ${𝒰}^{\prime }$. The processing of drug target information is described in sections on Drug targets and Cancer drug and target information.
- **scGen** [21]: scGen is a widely-used gold-standard latent variable model for response prediction [99–102]. Given a set of observed cell type in control and perturbed state, scGen predicts the response of a new cell type to the perturbagen seen in training. To utilize scGen as a baseline, we first fit it to our LINCS gene expression data for each dataset type to predict response to perturbagens, training one model per perturbagen (chemical or genetic). Then, given a sample of paired diseased-treated cell line states, $T=<x^{d},{𝒰}^{\prime },x^{t}>$, we compute the response of cell line with gene expression $x^{d}$ to all perturbagens. The predicted perturbagen is that whose predicted response is closest to $x^{t}$ in $R^{2}$ score. Since scGen trains one model per perturbagen, it needs an exhaustively long training time for datasets with a large number of perturbagens, especially in the leave-cell-out setting. Therefore, we set the maximum training epochs to 100 and only conducted leave-cell-out tests for one split of data for scGEN.
- **Biolord** [31]: Biolord can predict perturbagen response for both chemical and genetic datasets. We followed the official tutorial from the Biolord GitHub repository: https://github.com/nitzanlab/biolord, using the recommended parameters. To prevent memory and quota errors, we implemented two filtering steps: 1) Instead of storing the entire response gene expression (rGEX) matrix of all input (control) cells for each perturbagen, we only store a vector of the averaged rGEX of the input cells per perturbagen, which is necessary for calculating $R^{2}$ for evaluation. 2) During prediction, if the number of control cells exceeds 10,000, we randomly down-sample the control cells to 10,000. Similar to scGEN, we calculate the responses gene expression $x^{d\prime }$ for all perturbagens and use them to calculate $R^{2}$ to get the rank of predicted perturbagens.
- **ChemCPA** [30]: ChemCPA is specifically designed for chemical perturbation. We followed the official tutorials on GitHub for running this model (https://github.com/theislab/chemCPA), with all parameters set following the authors’ recommendations. Data processing was also conducted using the provided scripts. We constructed drug embedding using RDKit with canonical SMILES sequences, as this is the default setting in the model and the tutorial. Since the original ChemCPA model lacks functionality to obtain the predicted rGEX for each drug (averaging over the dosages), we developed a custom script to perform this task. These predictions were subsequently used for calculating $R^{2}$ to get the rank of predicted perturbagens/targets.
- **GEARS** [32]: GEARS is capable of predicting perturbagen responses for genetic perturbation datasets, specifically for predicting the rGEX to unseen perturbagens. However, it is limited to predicting only those genes that are present in the gene network used as prior knowledge for model training. Additionally, GEARS cannot process perturbagens with only one sample, so we filtered the data accordingly. We followed the official tutorial from the GEARS GitHub repository (https://github.com/snap-stanford/GEARS), using the recommended parameters. After confirming with the authors, we established that GEARS is suitable only for within-cell-line prediction. Consequently, our experiments with GEARS were conducted exclusively within this scenario.
- **CellOT** [26]: CellOT is capable of working with both chemical and genetic datasets. We ran this model by following the official tutorial from GitHub (https://github.com/bunnech/cellot), ensuring that all parameters were set according to the provided guidelines. Due to CellOT’s limitation in processing perturbagens with small sample sizes, we filtered the data to retain only those perturbagens with more than five samples or cells. We then used the predicted response gene expression $x^{d\prime }$ to calculate $R^{2}$ and the predicted perturbagen ranks. Similar to scGEN, CellOT trains one model per perturbagen, which results in an exhaustively long training time for datasets with a large number of perturbagens. This issue becomes even more significant when applying cross-cell line comparisons. Therefore, for this method, we set the maximum training epochs to 100 and only conduct one split in cross-cell line tests.

### Dataset splits and evaluation settings.

We evaluate PDGrapher and baseline methods on two different settings:

- **Systematic random dataset splits:** Our dataset is split randomly into train and test sets to measure our model performance in an IID setting.
- **Leave-cell-line-out dataset splits:** To measure model performance on unseen cell lines, we train our model with random splits on one cell line and test on a new cell line. Specifically, for chemical perturbation data, we train a model for each random split per cell line and test it on the entire dataset of the remaining eight cell lines. For genetic data, we train a model for each random split per cell line and test it on the entire dataset of the remaining nine cell lines.

### Evaluation setup.

For all dataset split settings, our model is trained using 5-fold cross-validation, and metrics are reported as the average on the test set. Within each fold, we further split the training set into training and validation sets (8:2) to perform early stopping: we train the model on the training set until the validation loss has not decreased at least 10^−5^ for 15 continuous epochs.

### Evaluation metrics.

We report average sample-wise $R^{2}$ score, and average perturbagen-wise $R^{2}$ score to measure performance in the prediction of $x^{t}$. The sample-wise $R^{2}$ score is computed as the square of Pearson correlation between the predicted sample ${\overset{ˆ}{x}}^{t}\in R^{N}$ and real sample $x^{t}\in R^{N}$. The perturbagen-wise $R^{2}$ score is adopted from scGen. It is computed as the square of Pearson correlation of a linear least-squares regression between a set of predicted treated samples ${\overset{ˆ}{X}}^{t}\in R^{N\times S}$ and a set of real treated samples $X^{t}\in R^{N\times S}$ for the same perturbagen. Higher values indicate better performance in predicting the treated sample $x^{t}$ given the diseased sample $x^{d}$ and predicted perturbagen. When evaluating competing methods that cannot predict perturbagen ranks for chemical perturbation data, we first calculate the rank of drugs based on the $R^{2}$ score. We then build a target gene rank from the drug rank by substituting the drugs with their target genes acquired from DrugBank [78] (accessed in November 2022) (see details in the data section). Notably, a single drug can have multiple target genes, which we place in the rank in random order. Since some methods cannot predict unseen drugs, their predicted target gene lists are often short, introducing bias in evaluation. To address this, we shuffle the missing target genes and attach them to the predicted ranks to create a complete rank. For genetic perturbation data, we directly obtain the target gene rank from the results, then attach the shuffled missing genes to the rank.

We also report the average ranking of real therapeutic gene targets in the predicted ordered list of therapeutic targets to measure the ability of our model to rank targets correctly. We normalize the ranking to the range [0, 1] as 1-ranking/N where N is the total number of genes in our dataset. Higher values indicate better performance; that is, the model ranks ground truth therapeutic targets closer to the top of the predicted list. In addition, we report the proportion of test samples for which the predicted therapeutic targets set has at least one overlapping gene with the ground-truth therapeutic targets set. We also calculated the ratio of correct perturbagens that appeared in the top 1, top 10, and top 100 predicted perturbagens in the predicted rank, denoted as recall@1, recall@10, and recall@100, respectively.

### Spatial enrichment analysis of PDGrapher’s predicted genes.

We quantify the spatial enrichment for PDGrapher ‘s predicted therapeutic targets using SAFE [29], a systematic approach that identifies regions that are over-represented for a feature of interest (Figure S5). SAFE requires networks and annotations for each node as an input. We use the PPI network as input and label gene nodes based on PDGrapher’s predictions: nodes are labeled as 1 if they are predicted to belong to the therapeutic targets set, and 0 otherwise. We compute enrichment analyses for two chemical compounds in the lung cancer cell line A540 test set: Raloxifene and Sertindole. We apply SAFE with the recommended settings: neighborhoods are defined using the short-path weighted layout metric for node distance and neighborhood radius of 0.15, and p-values are computed using the hypergeometric test with multiple testing correction (1,000 iterations). We use the Python implementation of SAFE: https://github.com/baryshnikova-lab/safepy.

### Ablation studies.

In the ablation study, we evaluated PDGrapher by running it with only the supervision loss (excluding the cycle loss) and with only the cycle loss (excluding the supervision loss) across all the chemical cell lines. We then compared the perturbagen prediction performance of these sub-models with that of PDGrapher. For each cell line, we maintained the same GNN layers across all models, selecting them based on the validation set’s performance of PDGrapher.

### Sensitivity studies.

To test the sensitivity of PDGrapher on protein-protein interactions (PPIs), we utilized data from STRING (string-db.org), which provides a confidence score for each edge. The specific dataset used was ”9606.protein.physical.links.detailed.v12.0.txt.gz” for Homo sapiens. We filtered the edges by using the 0.1, 0.2, 0.3, 0.4, and 0.5 quantiles of the confidence scores as cutoffs, resulting in five PPI networks with 625,818, 582,305, 516,683, 443,051, and 296,451 edges, respectively. Subsequently, we further filtered these PPIs to retain only the nodes (proteins) that were present in the gene expression data. For the sensitivity tests, we selected two cell lines: MDAMB231 for chemical data and MCF7 for genetic data. For each cell line, we processed the data as described above using the five PPI networks. We also ran PDGrapher with 1, 2, and 3 GNN layers, selecting the best configuration based on the performance of the validation set.

### Synthetic datasets.

We generated two synthetic datasets: (1) dataset with missing components: this dataset is generated by progressively removing bridge edges from the existing PPI network. Bridge edges are those whose removal disconnects parts of the network. We vary the fraction of bridge edges removed in increments (from 0 to 1), and for each fraction, we create a new edge list representing the modified network (Table S5). This process ensures that different levels of network sparsity are introduced, affecting the overall structure and connectivity. We pair these networks with gene expression data from Chemical-PPI-Breast-MDAMB231. (2) dataset with latent confounder noise: our starting point is the Chemical-PPI-Breast-MDAMB231 dataset. We simulate latent confounder bias in the treated gene expression data by introducing different Gaussian noise distributions to distinct subsets of genes across the network. First, genes are grouped into random subsets, and for each subset, a unique Gaussian noise distribution, defined by distinct means and variances, is applied. The fraction of gene groups (network communities) affected increases progressively (from 0 to 1), thus introducing varying degrees of confounding bias in the treated gene expression across the network. This strategy ensures that different latent biases are introduced globally to gene expression patterns while maintaining controlled variability. We pair the noisy version of the gene expression data with the global unperturbed PPI network.

## Supplementary Material

Supplement 1

Supplement 2

## Figures and Tables

**Table 1:** Table shows several healthy, diseased, and treated samples for lung cancer (A549), breast cancer (MCF7), and prostate cancer (PC3), and only diseased and treated samples for skin cancer (A375), colon cancer (HT29), ovarian cancer (ES2), head and neck cancer (BICR6), pancreatic cancer (YAPC), stomach cancer (AGS), and brain cancer (U251MG) with genetic perturbations.

Cancer type	Cell line	Sample type	N samples	Category	N perturbagens

Lung cancer	A549	healthy	50	vehicle	-
		diseased	4,327	vector	-
		treated	24,255	CRISPR	3,711

Breast cancer	MCF7	healthy	113	untreated	-
		diseased	4,852	vector	-
		treated	18,774	CRISPR	3,090

Prostate cancer	PC3	healthy	185	vector	-
		diseased	6,890	vector	-
		treated	21,229	CRISPR	3,710

Skin cancer	A375	diseased	4,777	vector	-
		treated	21,794	CRISPR	3,709

Colon cancer	HT29	diseased	4,235	vector	-
		treated	20,525	CRISPR	3,706

Ovary cancer	ES2	diseased	1,277	vector	-
		treated	23,708	CRISPR	3,654

Head and Neck	BICR6	diseased	1,362	vector	-
		treated	21,183	CRISPR	3,711

Pancreas cancer	YAPC	diseased	1,275	vector	-
		treated	20,135	CRISPR	3,711

Stomach cancer	AGS	diseased	1,352	vector	_-_
		treated	21,284	CRISPR	3,712

Brain cancer	U251MG	diseased	1,449	vector	-
		treated	26,323	CRISPR	3,712

**Table 2:** Table shows several healthy, diseased, and treated samples for lung cancer (A549), breast cancer (MCF7, MDAMB231, and BT20), and prostate cancer (PC3 and VCAP), and only diseased and treated samples for cervical cancer (HELA), colon cancer (HT29), and skin cancer (A375) with chemical perturbations.

Cancer type	Cell line	Sample type	N samples	Category	N perturbagens

Lung cancer	A549	healthy	50	vehicle	-
		diseased	5,261	vehicle	-
		treated	23,100	compound	1,041

Breast cancer	MCF7	healthy	2,675	untreated	-
		diseased	7,336	vehicle	-
		treated	35,421	compound	1,154

Breast cancer	MDAMB231	healthy	2,675	untreated	-
		diseased	1,591	vehicle	-
		treated	10,004	compound	526

Breast cancer	BT20	healthy	2,675	untreated	-
		diseased	409	vehicle	-
		treated	1,403	compound	39

Prostate cancer	PC3	healthy	185	vector	-
		diseased	7,202	vehicle	-
		treated	32,555	compound	1,182

Prostate cancer	VCAP	healthy	185	untreated	-
		diseased	3,904	vehicle	-
		treated	7,364	compound	738

Colon cancer	HT29	diseased	4,317	vehicle	-
		treated	19,386	compound	1,053

Skin cancer	A375	diseased	5,165	vehicle	-
		treated	25347	compound	1,093

Cervix cancer	HELA	diseased	2,905	vehicle	-
		treated	20,308	compound	683

**Table 3:** Our problem formulation is similar to conventional node and graph classification tasks, albeit some major differences exist.

Task	Number of graphs	Number of node attribute sets	Label dimensions

Graph Classification	m	m	m × 1 (one for each graph)
Node Classification	1	1	1 × n (one for each node)
Ours	1	m	m × n (one for each node of each graph)

## Data Availability

Processed data used in this paper, including the cell line gene expression dataset, protein-protein interaction network, drug targets, and disease-associated genes, are available via the project website at https://zitniklab.hms.harvard.edu/projects/PDGrapher or directly at https://figshare.com/articles/dataset/Combinatorial_prediction_of_therapeutic_targets_using_a_causally-inspired_neural_network/24798855. The raw protein-protein interaction network data was obtained from https://downloads.thebiogrid.org/File/BioGRID/Release-Archive/BIOGRID-3.5.186/BIOGRID-MV-Physical-3.5.186.tab3.zip, https://www.science.org/doi/suppl/10.1126/science.1257601/suppl_file/datasets_s1-s4.zip, and http://www.interactome-atlas.org/data/HuRI.tsv. Raw gene expression datasets were obtained from https://clue.io/releases/data-dashboard. Disease-associated genes were obtained from COSMIC at https://cancer.sanger.ac.uk/cell_lines/archive-download#:~:text=Complete%20mutation%20data and https://cancer.sanger.ac.uk/cosmic/curation. Drug targets were extracted from DrugBank at https://go.drugbank.com/releases/5-1-9, and a list of cancer drugs was obtained from NCI at https://www.cancer.gov/about-cancer/treatment/types/targeted-therapies/approved-drug-list.
