# Combinatorial prediction of therapeutic perturbations using causally-inspired neural networks

**DOI:** 10.1101/2024.01.03.573985

**Published:** 2025-01-28

**Authors:** Guadalupe Gonzalez, Xiang Lin, Isuru Herath, Kirill Veselkov, Michael Bronstein, Marinka Zitnik

**Affiliations:** 1 Imperial College London, London, UK; 2 Prescient Design, Genentech, South San Francisco, CA, USA; 3 F. Hoffmann-La Roche Ltd, Basel, Switzerland; 4 Merck & Co., South San Francisco, CA, USA; 5 Cornell University, Ithaca, NY, USA; 6 University of Oxford, Oxford, UK; 7 Harvard Medical School, Boston, MA, USA; 8 Kempner Institute for the Study of Natural and Artificial Intelligence, Harvard University, Cambridge, MA, USA; 9 Broad Institute of MIT and Harvard, Cambridge, MA, USA; 10 Harvard Data Science Initiative, Cambridge, MA, USA

## Abstract

Phenotype-driven approaches identify disease-counteracting compounds by analyzing the phenotypic signatures that distinguish diseased from healthy states. These approaches can guide the discovery of targeted perturbations, including small-molecule drugs and genetic interventions, that modulate disease phenotypes toward healthier states. Here, we introduce PDGrapher, a causally inspired graph neural network (GNN) designed to predict combinatorial perturbagens (sets of therapeutic targets) capable of reversing disease phenotypes. Unlike methods that learn how perturbations alter phenotypes, PDGrapher solves the inverse problem of directly predicting the perturbagens needed to achieve a desired response. PDGrapher is a GNN that embeds disease cell states into gene regulatory or protein-protein interaction networks, learns a latent representation of these states, and identifies the optimal combinatorial perturbations that most effectively shift the diseased state toward the desired treated state within that latent space. In experiments in nine cell lines with chemical perturbations, PDGrapher identified effective per-turbagens in up to 13.33% more test samples than competing methods and achieved a normalized discounted cumulative gain of up to 0.12 higher to classify therapeutic targets. It also demonstrated competitive performance on ten genetic perturbation datasets. A key advantage of PDGrapher is its direct prediction paradigm, in contrast to the indirect and computationally intensive models traditionally employed in phenotype-driven research. This approach accelerates training by up to 25 times compared to existing methods. PDGrapher provides a fast approach for identifying therapeutic perturbations and advancing phenotype-driven drug discovery.

## Main

Target-driven drug discovery, which has been the dominant approach since the 1990s, focuses on designing highly specific compounds to act against targets, such as proteins or enzymes, that are implicated in disease, often through genetic evidence [1–3]. An example of target-driven drug discovery is the development of small-molecule kinase inhibitors like imatinib. Imatinib stops the progression of chronic myeloid leukemia (CML) by inhibiting BCR-ABL tyrosine kinase, a mutated protein involved in uncontrolled proliferation of leukocytes in patients with CML [4]. Another notable example is monoclonal antibodies such as trastuzumab, which specifically targets the HER2 receptor, a protein overexpressed in certain types of breast cancer. Trastuzumab inhibits cell proliferation while engaging the body’s immune system to initiate anti-cancer response [5]. These examples illustrate the success of target-driven drug discovery, yet the past decade has seen a revival of phenotype-driven approaches. This shift has been fueled by the observation that many first-in-class drugs approved by the US Food and Drug Administration (FDA) between 1999 and 2008 were discovered without a drug target hypothesis [6]. Instead of the ”one drug, one gene, one disease” model of target-driven approaches, phenotype-driven drug discovery focuses on identifying compounds or, more broadly, perturbagens—combinations of therapeutic targets—that reverse phenotypic disease effects, as measured by phenotypic assays without predefined targets [1, 7]. While ivacaftor was developed through a target-driven approach by modulating the cystic fibrosis transmembrane conductance regulator protein in individuals with cystic fibrosis and specific mutations, its development used phenotypic assays to confirm functional improvements, such as increased chloride transport [8–10].

Phenotype-driven drug discovery has been bolstered by the advent of chemical and genetic libraries such as the Connectivity Map (CMap [11]) and the Library of Integrated Network-based Cellular Signatures (LINCS [12]). CMap and LINCS contain gene expression profiles of dozens of cell lines treated with thousands of genetic and chemical perturbagens. CMap introduced connectivity scores to quantify similarities between compound response and disease gene expression signatures. Identifying compounds with gene expression signatures either similar to those of known disease-treating drugs or that counter disease signatures can help in selecting therapeutic leads [13–16]. These strategies have successfully identified drugs that demonstrate high in vitro efficacy [16] for a variety of diseases [17–19].

Deep learning methods have been used for lead discovery by predicting gene expression responses to perturbagens, including perturbagens that were not yet experimentally tested [20–23]. However, these approaches rely on chemical and genetic libraries, meaning they select per-turbagens from predefined libraries and cannot identify perturbagens as combinations of drug targets. Further, these approaches are predominantly perturbation response methods that predict changes in phenotypes upon perturbations. Thus, they can identify perturbagens by exhaustively predicting responses to all perturbations in the library and then searching for perturbagens with the desired response. Unlike existing methods that learn responses to perturbations, phenotype-based approaches needs to solve the inverse problem, which is to infer perturbagens necessary to achieve a specific response – i.e., directly predicting perturbagens by learning which perturbations elicit a desired response.

In causal discovery, the problem of identifying which elements of a system should be per-turbed to achieve a desired state is referred to as optimal intervention design [24–26]. Using insights from causal discovery and geometric deep learning, here we introduce PDGrapher, an approach to combinatorial prediction of therapeutic targets that can shift gene expression from an initial diseased state to a desired treated state. PDGrapher is formulated using a causal model, where genes represent the nodes in a causal graph, and structural causal equations define their causal relationships. Given a genetic or chemical intervention dataset, PDGrapher pinpoints a set of genes that a perturbagen should target to facilitate the transition of node states from diseased to treated. PDGrapher utilizes protein-protein interaction networks (PPI) and gene regulatory networks (GRN) as approximations of the causal graph, operating under the assumption of no unobserved confounders. PDGrapher tackles the optimal intervention design using representation learning, using a graph neural network (GNN) to represent structural equations.

PDGrapher is trained on a dataset of disease-treated sample pairs to predict therapeutic gene targets that can shift the gene expression phenotype from a diseased to a normal state. Once trained, PDGrapher processes a new diseased sample and outputs a perturbagen – a combination or set of therapeutic targets – predicted to counteract the disease effects in that specific sample. We evaluate PDGrapher across 19 datasets, comprising genetic and chemical interventions across 11 cancer types and two proxy causal graphs, and consider different evaluation setups, including settings where held out folds contain new samples and settings where held out folds contain new samples from a cancer type that PDGrapher has never encountered during training. In held out folds that contain new samples, PDGrapher ranks ground-truth therapeutic targets up to 12.58% higher in chemical intervention datasets than existing methods and comparable to existing methods in genetic intervention datasets. Even in held-out folds containing new samples from a previously unseen disease, PDGrapher maintains robust performance. Unlike methods that indirectly identify perturbagens by predicting cell responses, PDGrapher directly predicts perturbagens that can shift gene expression from diseased to treated states. This feature of PDGrapher enables model training up to 25 times faster than indirect prediction methods such as scGen [22] and CellOT [27]. Since these approaches build a separate model for each perturbation, they become almost unusable for data with many perturbagens. For example, with the default setting, cellOT needs 10 hours to train for a single perturbagen in a cell line from the LINCS dataset. We find that in chemical intervention datasets, candidate therapeutic targets predicted by PDGrapher are on average up to 11.58% closer to ground-truth therapeutic targets in the gene-gene interaction network than what would be expected by chance. Additionally, PDGrapher can aid in elucidating the mechanism of action of chemical perturbagens (Figure S5), which we show in the case of vorinonstat, a histone deacety-lase inhibitor used to treat cutaneous T-cell lymphoma, and sorafeniv, a multi-kinase inhibitor used in the treatment of several types of cancers (Supplementary Note 1). PDGrapher can suggest potential anti-cancer drug targets. It highlighted KDR as a top-predicted target for non-small cell lung cancer. It identified associated drugs, vandetanib, sorafenib, catequentinib and rivoceranib, which inhibit KDR’s kinase activity. These drugs block VEGF signaling, suppressing endothelial cell proliferation, migration, and blood vessel formation that tumors rely on for growth and metastasis [28, 29]. By predicting combinatorial therapeutic targets based on phenotypic transitions, PDGrapher provides a scalable and efficient approach to phenotype-driven perturbation modeling.

## Results

### Overview of perturbation datasets and gene-gene networks.

We consider a total of 19 datasets across two treatment types (genetic and chemical interventions), 11 cancer types (lung cancer, breast cancer, prostate cancer, colon cancer, skin cancer, cervical cancer, head and neck cancer, pancreatic cancer, stomach cancer, brain cancer, and ovarian cancer), and two proxy causal graphs (protein-protein interaction network (PPI), and gene regulatory networks (GRN)), which we denote as follows: Chemical-PPI-Lung-A549, Chemical-PPI-Breast-MCF7, Chemical-PPI-Breast-MDAMB231, Chemical-PPI-Breast-BT20, Chemical-PPI-Prostate-PC3, Chemical-PPI-Prostate-VCAP, Chemical-PPI-Colon-HT29, Chemical-PPI-Skin-A375, and Chemical-PPI-Cervix-HELA, Genetic-PPI-Lung-A549, Genetic-PPI-Breast-MCF7, Genetic-PPI-Prostate-PC3, Genetic-PPI-Skin-A375, Genetic-PPI-Colon-HT29, Genetic-PPI-Ovary-ES2, Genetic-PPI-Head-BICR6, Genetic-PPI-Pancreas-YAPC, Genetic-PPI-stomach-AGS, and Genetic-PPI-Brain-U251MG, Chemical-GRN-Lung-A549, Chemical-GRN-Breast-MCF7, Chemical-GRN-Breast-MDAMB231, Chemical-GRN-Breast-BT20, Chemical-GRN-Prostate-PC3, Chemical-GRN-Prostate-VCAP, Chemical-GRN-Colon-HT29, Chemical-GRN-Skin-A375, and Chemical-GRN-Cervix-HELA, Genetic-GRN-Lung-A549, Genetic-GRN-Breast-MCF7, Genetic-GRN-Prostate-PC3, Genetic-GRN-Skin-A375, Genetic-GRN-Colon-HT29, Genetic-GRN-Ovary-ES2, Genetic-GRN-Head-BICR6, Genetic-GRN-Pancreas-YAPC, Genetic-GRN-Stomach-AGS, and Genetic-GRN-Brain-U251MG. Genetic interventions are single-gene knockout experiments by CRISPR/Cas9-mediated gene knockouts, while chemical interventions are multiple-gene treatments induced using chemical compounds. We utilize a PPI network that has 10,716 nodes and 151,839 undirected edges. We additionally construct gene regulatory networks for each disease-treatment type pair using a gene regulatory network inference method (Online Methods) with GRNs on average having 10,000 nodes and 500,000 directed edges. Each dataset is made up of disease intervention data, if available, and treatment intervention data. For cell lines corresponding to lung, breast, and prostate cancer, the disease intervention data contains paired healthy and diseased gene expression samples and disease-associated genes. For the remaining cancer types, healthy control samples are not available. Treatment intervention data contains paired diseased and treated gene expression samples and genetic or chemical perturbagens. Tables 1 and 2 summarize the number of samples for each cell line and intervention dataset type.

### Overview of PDGrapher model.

**Figure 1:**
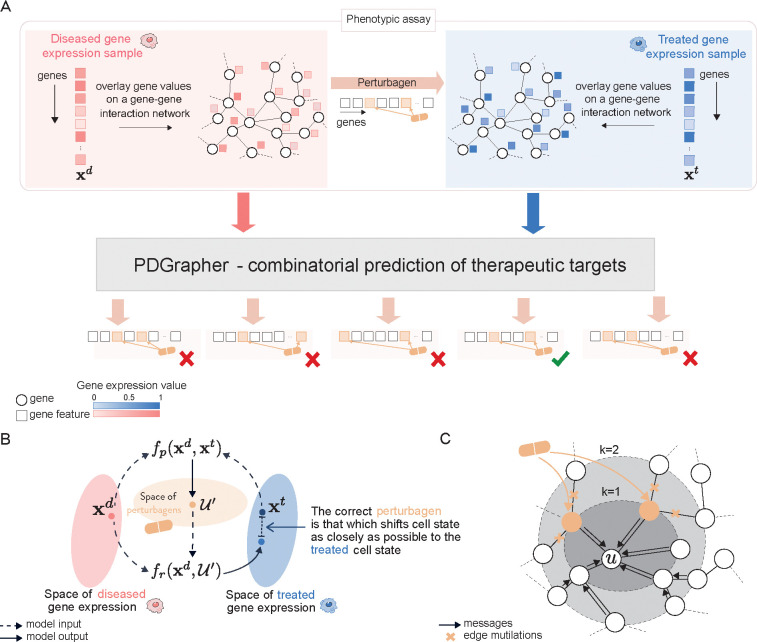
Overview of PDGrapher. **(A)** Given a paired diseased and treated gene expression samples and a proxy causal graph, PDGrapher predicts a candidate set of therapeutic targets to shift cell gene expression from diseased to treated state. **(B)** PDGrapher is comprised by two modules. A perturbagen discovery module $f_{p}$ that, given a pair of diseased and treated gene expression samples, computes a candidate set of therapeutic targets ${𝒰}^{\prime }$, and a response prediction module $f_{r}$ that predicts the response of the diseased sample to the predicted candidate perturbagen. $f_{p}$ is optimized using 2 losses: a cross-entropy cycle loss to predicted a perturbagen ${𝒰}^{\prime }$ which would shift diseased cell state to a state as close as possible to the treated state: $CE\left(x^{t},f_{r}\left(x^{d},f_{p}\left(x^{d},x^{t}\right)\right)\right)$ (with $f_{r}$ frozen), and a cross-entropy supervision loss that directly supervises the prediction of ${𝒰}^{\prime }:\;CE\left({𝒰}^{\prime },f_{p}\left(x^{d},x^{t}\right)\right)$. See Methods for more details. **(C)** PDGrapher has two modules, $f_{r}$ and $f_{p}$, both based on GNNs. Depicted is $f_{r}$, which takes as input a diseased gene expression and a perturbagen (therapeutic gene set) and represents perturbagens effects in the graph as edge mutilations. Both $f_{r}$ and $f_{p}$ follow the standard message-passing framework where node representations are updated by aggregating the information from neighbors in the graph.

Given a diseased cell state (gene expression), the goal of PDGrapher is to predict the genes that, if targeted by a perturbagen on the diseased cell, would shift the cell to a treated state (Figure 1A). Unlike methods for learning the response of cells to a given perturbation [22,27,30,31], PDGrapher focuses on the inverse problem by learning which perturbation elicits a desired response. PDGrapher predicts perturbagens to shift cell states under the assumption that an optimal perturbagen will be that which shifts the cell gene expression as close as possible to the desired gene expression. Our approach comprises two modules (Figure 1B). First, a perturbagen discovery module $f_{p}$ takes the initial and desired cell states and outputs a candidate perturbagen as a set of therapeutic targets ${𝒰}^{\prime }$. Then, a response prediction module $f_{r}$ takes the initial state and the predicted perturbagen ${𝒰}^{\prime }$ and predicts the cell response upon perturbing genes in ${𝒰}^{\prime }$. Our response prediction and perturbagen discovery modules are graph neural network (GNN) models that operate on a proxy causal graph, where edge mutilations represent the effects of interventions on the graph (Figure 1C).

PDGrapher is trained using an objective function with two components, one for each module, $f_{r}$ and $f_{p}$. The response prediction module $f_{r}$ is trained using all available data on cell state transitions (that is, disease and treatment intervention data). Response prediction module $f_{r}$ is trained so cell states are close to the known perturbed cell states upon interventions. The perturbagen discovery module $f_{p}$ is trained using treatment intervention data; given a diseased cell state, $f_{p}$ predicts the set of therapeutic targets ${𝒰}^{\prime }$ that caused the corresponding treated cell state. The objective function for the perturbagen discovery module consists of two elements: (1) a cycle loss that optimizes the parameters of $f_{p}$ such that the response upon intervening on the predicted genes in ${𝒰}^{\prime }$, as measured by $f_{r}$, is as close as possible to the real treated state and (2) a supervision loss on the therapeutic targets set ${𝒰}^{\prime }$ that directly pushes PDGrapher to predict the correct perturbagen. Both models are trained simultaneously using early stopping independently so that each model finishes training upon convergence.

**Figure 2:**
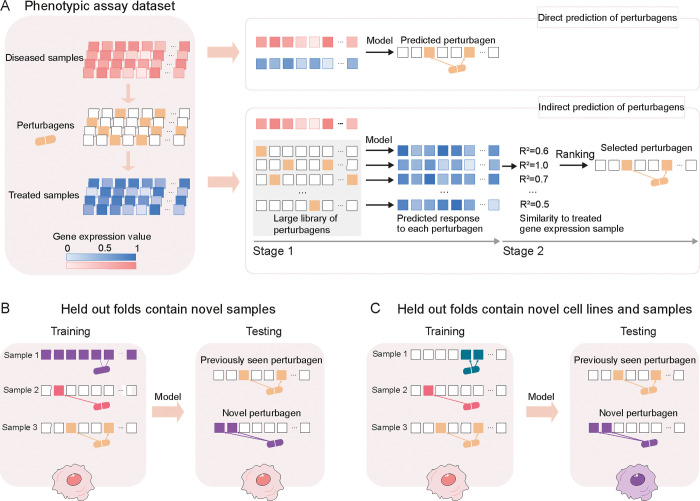
Overview of evaluation settings and data splits. **(A)** Given a dataset with paired diseased and treated samples and a set of perturbagens, PDGrapher makes a direct prediction of candidate perturbagens that shift gene expression from a diseased to a treated state, for each disease-treated sample pair. The direct prediction means that PDGrapher directly infers the perturbation necessary to achieve a specific response. In contrast to direct prediction of perturbagens, existing methods predict perturbagens only indirectly through a two-stage approach: for a given diseased sample, they learn the response to each one of the perturbagen candidates from an existing library upon intervention and return the perturbagen whose response is as close as possible to the desired treated state. Existing methods learn the response of cells to a given perturbation [22,27,30,31], whereas PDGrapher focuses on the inverse problem by learning which perturbagen elicit a given response, even in the most challenging cases when the combinatorial composition of perturbagen was never seen before. **(B-C)** We evaluate PDGrapher’s performance across two settings: randomly splitting samples between training and test set (B), and splitting samples based on the cell line where we train in a cell line and evaluate PDGrapher’s performance on another cell line the model never encountered before (C).

When trained, PDGrapher predicts perturbagens (as a set of candidate target genes) to shift cells from diseased to treated. Given a pair of diseased and treated samples, PDGrapher directly predicts perturbagens by learning which perturbations elicit target responses. In contrast, existing approaches are perturbation response methods that predict changes in phenotype that occur upon perturbation, thus, they can only indirectly predict perturbagens (Figure 2A). Given a disease-treated sample pair, a response prediction module (such as scGen [22], ChemCPA [32], Biolord [33], GEARS [34], and CellOT [27]) is used to predict the response of the diseased sample to a library of perturbagens. The predicted perturbagen is the one that produces a response that is the most similar to the treated sample. We evaluate PDGrapher’s performance in two separate settings (Figure 2BC): (1) a random splitting setting, where the samples are split randomly between training and test sets, and (2) a leave-cell-out setting, where PDGrapher is trained in one cell line, and its performance is evaluated in a cell line the model never encountered during training to test how well the model generalizes to a new disease. Tables S1 and S2 show the numbers of unseen perturbagens in the random and leave-cell-out splits in chemical perturbation datasets, respectively; Table S3 and S4 show the number of unseen perturbagens in the random and leave-cell-out splits in genetic perturbation datasets, respectively.

### PDGrapher predicts genetic and chemical perturbations to reverse disease phenotypes.

In the random splitting setting, we assess the ability of PDGrapher for combinatorial prediction of therapeutic targets across chemical PPI datasets (Chemical-PPI-Lung-A549, Chemical-PPI-Breast-MCF7, Chemical-PPI-Breast-MDAMB231, Chemical-PPI-Breast-BT20, Chemical-PPI-Prostate-PC3, Chemical-PPI-Prostate-VCAP, Chemical-PPI-Colon-HT29, Chemical-PPI-Skin-A375, and Chemical-PPI-Cervix-HELA). Specifically, we measure whether, given paired diseased-treated gene expression samples, PDGrapher can predict the set of therapeutic genes targeted by the chemical compound in the diseased sample to generate the treated sample. Given paired diseased-treated gene expression samples, PDGrapher ranks genes in the dataset according to their likelihood of being in the set of therapeutic targets.

**Figure 3:**
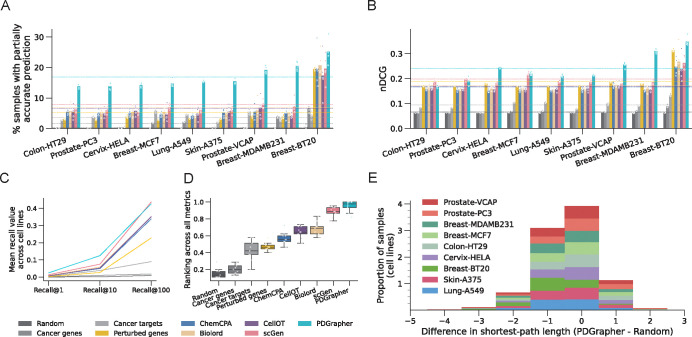
PDGrapher efficiently predicts chemical perturbagens to shift cells from diseased to treated states in held out folds containing novel samples. (A) PDGrapher provides partially accurate predictions in up to 13.37% (Chemical-PPIBreast-MDAMB231: 20.43% vs 7.05%) more samples in the test set compared to the second-best baseline. **(B)** PDGrapher achieves up to 0.12 (Chemical-PPI-Breast-MDAMB231: 0.31 vs 0.18) higher nDCG than the second-best baseline in the predicted list across Chemical-PPI datasets. **(C)** PDGrapher recovers ground-truth therapeutic targets at higher rates compared to competing methods for Chemical-PPI datasets. **(D)** The boxplots show the ranks across the experiments of different cell lines and metrics for each method. Higher value indicates better performance. The central line inside the box represents the median, while the top and bottom edges correspond to the first (Q1) and third (Q3) quartiles. The whiskers extend to the smallest and largest values within 1.5 times the interquartile range (IQR) from the quartiles. Each individual dot represents a data point for a specific cell line and metrics. **(E)** Shown is the difference of shortest-path distances between ground-truth therapeutic genes and predicted genes by PDGrapher and a Random baseline. Contributions across cell lines are stacked for each bin, leading to y-axis values exceeding 1 in some cases. Predominantly negative values indicate that PDGrapher predicts sets of therapeutic genes that are closer in the network to ground-truth therapeutic genes compared to what would be expected by chance [average shortest-path distances across cell lines for PDGrapher vs Random = 2.77 vs 3.11].

We quantify the ranking quality using normalized discounted cumulative gain (nDCG), where the gain reflects the ranking accuracy of the model. An nDCG value close to 1 indicates highly accurate predictions, with the top-ranked gene targets closely matching the ground-truth targets, while lower nDCG values indicate poorer ranking performance. This metric provides a normalized and scalable measure of ranking quality, enabling consistent comparison across different datasets and models. PDGrapher outperforms competing methods in all cell lines, achieving 0.02 (Chemical-PPI-Lung-A549), 0.13 (Chemical-PPI-Breast-MDAMB231), 0.03 (Chemical-PPI-Breast-BT20), 0.004 (Chemical-PPI-Breast-MCF7), 0.07 (Chemical-PPI-Prostate-VCAP), 0.005 (Chemical-PPI-Prostate-PC3), 0.03 (Chemical-PPI-Skin-A375), 0.06 (Chemical-PPI-Cervix-HELA), and 0.001 (Chemical-PPI-Colon-HT29), higher nDCG values compared to the second-best competing method (Figure 3B). In addition to evaluating the entire predicted target rank, it is even more practically crucial to assess the accuracy of the top-ranked predicted targets. To achieve this, we use partial accuracy and recall to evaluate the performance of PDGrapher and competing methods. Because perturbagens target multiple genes, we measure the fraction of samples in the test set for which we obtain a partially accurate prediction, where at least one of the top N predicted gene targets corresponds to an actual gene target. Here, N represents the number of known target genes of a drug. PDGrapher consistently provides accurate predictions for more samples in the test set than competing methods. Specifically, it outperforms the second-best competing method by predicting ground-truth targets in an additional 7.73% (Chemical-PPI-Breast-MCF7), 9.32% (Chemical-PPILung-A549), 13.37% (Chemical-PPI-Breast-MDAMB231), 4.50% (Chemical-PPI-Breast-BT20), 7.88% (Chemical-PPI-Prostate-PC3), 11.53% (Chemical-PPI-Prostate-VCAP), 7.56% (Chemical-PPI-Colon-HT29), 9.55% (Chemical-PPI-Skin-A375), and 8.41% (Chemical-PPI-Cervix-HELA) of samples (Figure 3A). We also evaluate the performance of PDGrapher using recall@1, recall@10, and recall@100, which calculate the ratio of true target genes included in the top 1, top 10, and top 100 predicted target genes, respectively. Although the absolute recall values are modest due to the inherent difficulty of the task, PDGrapher consistently outperforms all competing methods, demonstrating its relative strength and robustness. Specifically, PDGrapher outperforms the second-best method in all the recall metrics with the averaged margin being 3.31% (Chemical-PPI-Lung-MCF7), 3.28% (Chemical-PPI-Lung-A549), 11.65% (Chemical-PPI-Breast-MDAMB231), 7.27% (Chemical-PPI-Breast-BT20), 2.50% (Chemical-PPI-Prostate-PC3), 9.53% (Chemical-PPI-Prostate-VCAP), 3.08% (Chemical-PPI-Colon-HT29), 2.87% (Chemical-PPI-Skin-A375), and 5.13% (Chemical-PPI-Cervix-HELA) (Figure 3C). We then consolidated the results using the rankings from experiments across different cell lines and metrics for each method. PDGrapher achieved the best overall rankings, with a median significantly higher than all competing methods. (Figure 3D). P-values of the chemical perturbagen discovery tests are provided in Table S9.

PDGrapher not only provides accurate predictions for a larger proportion of samples and consistently predicts ground-truth therapeutic targets close to the top of the ranked list, but it also predicts gene targets that are closer in the network to ground-truth targets compared to what would be expected by chance (Figure 3E). In all cell lines, the ground-truth therapeutic targets predicted by PDGrapher are significantly closer to the ground-truth targets compared to what would be expected by chance (Table S6). For example, for Chemical-PPI-Lung-A549, the median distance between the predicted and ground-truth therapeutic targets is 3.0 for both PDGrapher and the Random reference. However, the distributions exhibit a statistically significant difference, with a one-sided Mann-Whitney U test that yields a p-value < 0.001, an effect size (rank-biserial correlation) of 0.3531 (95% CI [0.3515, 0.3549]), and a U statistic of 1.29e11. Similarly, for Chemical-PPI-Breast-MCF7, the median distance is 3.0 for both groups, yet the distributions are significantly different (p-value < 0.001, effect size = 0.2160 (95% CI [0.2145, 0.2173]), U statistic = 3.91e11)(Table S6). This finding aligns with the local network hypothesis, which posits that genes in closer network proximity tend to exhibit greater similarity than those that are more distantly connected [35–37]. PDGrapher predicts targets in a manner that reflects protein-protein interactions [31].

Approaches that train individual models for each perturbagen (scGEN and CellOT) generally perform better in perturbagen prediction than those that train a single model for all perturbagens (Biolord, GEARS, and ChemCPA). However, the former approach is exhaustively time-consuming when applied to large-scale datasets with numerous perturbagens. For example, without parallel training, scGEN would require approximately 8 years to complete the leave-cell-out experiments for chemical and genetic perturbation data used in this study, which is intractable. The training process of PDGrapher is up to 25 times faster than scGEN and more than 100 times faster than CellOT if it is trained using the default setting of 100,000 epochs, significantly reducing the computational cost of predicting perturbagens and highlighting another advantage of PDGrapher over these methods. The enhanced efficiency of PDGrapher is attributed to its unique approach. Existing approaches are predominantly perturbation response methods that predict changes in the phenotype that occur upon perturbation. Thus, they can identify perturbagens only indirectly by exhaustively predicting responses to all perturbations in the library and then searching for the perturbagen with the desired response that reverses a disease state. Unlike methods that learn responses to perturbations, PDGrapher addresses the inverse problem, which is to infer the perturbagen necessary to achieve a specific response – i.e., directly predicting perturbagens by learning which perturbations elicit a desired response.

PDGrapher also exhibits superior performance across genetic datasets, specifically Genetic-PPI-Lung-A549, Genetic-PPI-Breast-MCF7, Genetic-PPI-Prostate-PC3, Genetic-PPI-Skin-A375, Genetic-PPI-Colon-HT29, Genetic-PPI-Ovary-ES2, Genetic-PPI-Head-BICR6, Genetic-PPI-Pancreas-YAPC, genetic-PPI-stomach-AGS, and Genetic-PPI-Brain-U251MG (Figure S3). Briefly, PDGrapher successfully detected real targets in 0.87% (Genetic-PPI-Lung-A549), 0.50% (Genetic-PPI-Breast-MCF7), 0.24% (Genetic-PPI-Prostate-PC3), 0.38% (Genetic-PPI-Skin-A375), 0.36% (Genetic-PPI-Colon-HT29), 1.09% (Genetic-PPI-Ovary-ES2), 0.54% (Genetic-PPI-Head-BICR6), 0.11% (Genetic-PPI-Pancreas-YAPC), and 0.92% (Genetic-PPI-Brain-U251MG) more samples compared to the second-best competing method (Figure S3A). Its ability to effectively predict targets at the top of the ranks is further supported by the metrics recall@1 and recall@10 (Figure S3C). P-values of the genetic perturbagen discovery tests are provided in Table S9. We observed that the performance of PDGrapher and the competing methods on genetic data are comparatively modest than those on chemical data. This relative modesty could stem from the inherent characteristics of knockout interventions, which often produce a reduced phenotypic signal compared to small molecule interventions. Studies have shown that while gene knockouts are essential for understanding gene function, single-gene knockout studies sometimes offer limited insights into complex cellular processes due to compensatory mechanisms [38–40]. Despite the modest performance in genetic intervention datasets, PDGrapher outperforms competing methods in the combinatorial prediction of therapeutic targets. We also observed superior performance of PDGrapher in response prediction for both chemical (Figure S1) and genetic data (Figure S3E–G). P-values of the response prediction tests are provided in Table S10.

When using GRNs as proxy causal graphs, we see that PDGrapher has comparable performance with GRN and PPI across both genetic and chemical intervention datasets (Figures S6 and S7). One difference is that gene regulatory networks were constructed individually for each cell line, which makes leave-cell-out splitting setting prediction particularly challenging. Therefore, we only conducted random splitting setting experiments for GRN datasets. We also use PDGrapher to illuminate the mode of action of chemical perturbagens vorinostat and sorafenib in Chemical-PPI-Lung-A549 (Supplementary Note 1).

### PDGrapher generalizes to new cell lines unseen during model training.

**Figure 4:**
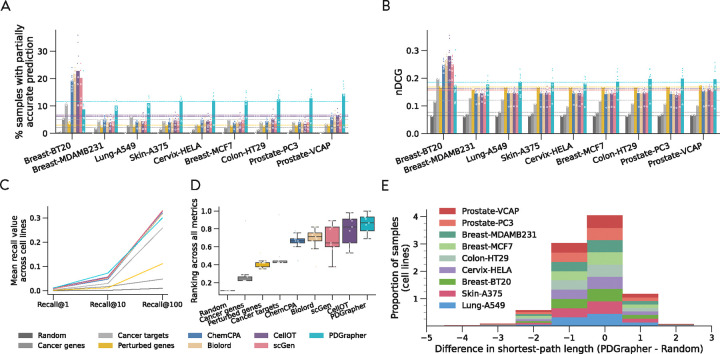
PDGrapher generalizes to new (previously unseen) cell lines and learns optimal chemical perturbagens in held out folds that contain both novel cell lines and novel samples. **(A)** PDGrapher provides partially accurate predictions in up to 8.67% (Chemical-PPI-Prostate-PC3: 12.81% vs 4.13%) more samples in the test set compared to the second-best baseline across Chemical-PPI datasets. **(B)** PDGrapher achieves up to 0.03 (Chemical-PPI-Colon-HT29: 0.19 vs 0.16) higher nDCG than the second-best baseline in the predicted list across Chemical-PPI datasets. **(C)** PDGrapher recovers ground-truth therapeutic targets at higher rates compared to competing methods for Chemical-PPI datasets. **(D)** The boxplots show the ranks across the experiments of different cell lines and metrics for each method. Higher value indicates better performance. The central line inside the box represents the median, while the top and bottom edges correspond to the first (Q1) and third (Q3) quartiles. The whiskers extend to the smallest and largest values within 1.5 times the interquartile range (IQR) from the quartiles. Each individual dot represents a data point for a specific cell line and metrics. **(E)** Shown is the difference of shortest-path distances between ground-truth therapeutic genes and predicted genes by PDGrapher and a Random baseline. Contributions across cell lines are stacked for each bin, leading to y-axis values exceeding 1 in some cases. Predominantly negative values indicate that PDGrapher predicts sets of therapeutic genes that are closer in the network to ground-truth therapeutic genes compared to what would be expected by chance [average shortest-path distances across cell lines for PDGrapher vs Random = 2.75 vs 3.11].

We observe consistently strong performance of PDGrapher in chemical intervention datasets in the leave-cell-out setting (Figure 4). In this setting, we use the trained models in the random-splitting setting for each cell line to predict therapeutic targets in the remaining cell lines. PDGrapher successfully predicts perturbagens that describe the cellular dynamics and shift gene expression phenotypes from a diseased to a treated state in 4.87% (Chemical-PPI-Breast-MCF7), 6.66% (Chemical-PPI-Lung-A549), 7.51% (Chemical-PPI-Breast-MDAMB231), 2.36% (Chemical-PPI-Prostate-PC3), 2.65% (Chemical-PPI-Prostate-VCAP), 7.31% (Chemical-PPI-Skin-A375), 1.47% (Chemical-PPI-Colon-HT29), and 3.16% (Chemical-PPI-Cervix-HELA) additional testing samples compared to the second-best competing method (Figure 4A). PDGrapher also outperforms the competing methods in six of out nine cell lines by predicting nDCG values 0.03 (Chemical-PPI-Breast-MCF7), 0.03 (Chemical-PPI-Lung-A549), 0.07 (Chemical-PPI-Breast-MDAMB231), 0.01 (Chemical-PPI-Prostate-VCAP), 0.03 (Chemical-PPI-Skin-A375), and 0.04 (Chemical-PPI-Cervix-HELA) higher than the second-best competing method (Figure 4B). Considering the overall performance across different cell lines and metrics, PDGrapher achieves the highest rank, with a median surpassing all competing methods (Figure 4D). P-values of the leave-cell-out perturbagen discovery tests and response prediction tests are provided in Table S9 and S10, respectively. Additionally, combinations of therapeutic targets predicted by PDGrapher in chemical datasets are closer to ground-truth targets than expected by chance (Figure 4E, Table S7). For example, for Chemical-PPI-Lung-A549, the median distance between predicted and ground-truth therapeutic targets is 3.0 for both PDGrapher and the Random reference. However, the distributions exhibit a statistically significant difference, with a one-sided Mann-Whitney U test yielding a p-value < 0.001, an effect size (rank-biserial correlation) of 0.2191 (95% CI [0.2182, 0.2200]), and a U statistic of 2.46e12. Similarly, for Chemical-PPI-Breast-MCF7, the median distance is 3.0 for both groups, yet the distributions are significantly different (p-value < 0.001, effect size = 0.2457 (95% CI [0.2450, 0.2464]), U statistic = 6.07e12) (Table S7). Besides chemical perturbations, PDGrapher outperforms existing methods in perturbagen prediction for genetic interventions across cell lines when considering the top targets on the predicted ranks (Figure S4A–D). PDGrapher also demonstrates superior performance in response prediction for both chemical (Figure S2) and genetic datasets (Figure S4E–G).

### PDGrapher predicts therapeutic targets for multiple cancer types.

**Figure 5:**
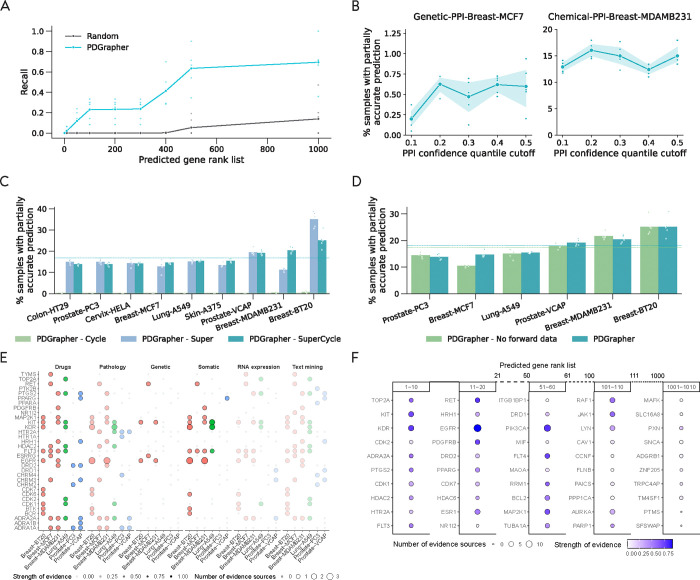
PDGrapher’s predictions illuminate mode of action of perturbagens. **(A)** Performance of PDGrapher in the prediction of unseen approved drug targets to reverse disease effects across all cell lines with healthy counterparts in chemical perturbation datasets. Individual datapoints represent individual cell lines. **(B)** Performance of sensitivity analyses evaluated by partial accuracy for cell line MDAMB231 and MCF7 with chemical and genetic perturbation, respectively. The PPI used here is from STRING (string-db.org) with a confidence score for each edge. The edges are filtered by the 0.1, 0.2, 0.3, 0.4, and 0.5 quantiles of the confidence scores as cutoffs, resulting in five PPI networks with 625,818, 582,305, 516,683, 443,051, and 296,451 edges, respectively. **(C)** Performance metrics of ablation study on PDGrapher’s objective function components: PDGrapher-Cycle trained using only the cycle loss, PDGrapher-SuperCycle trained using the supervision and cycle loss, and PDGrapher-Super trained using only the supervision loss, evaluated by partial accuracy.PDGrapher-Cycle demonstrates inferior performance, resulting in limited visibility in the bar plot. **(D)** Performance metrics of the second ablation study on PDGrapher’s input data: PDGrapher- No forward data using only backward data and PDGrapher using both forward and backward data. The forward and backward data are organized as (healthy, mutation, disease) and (diseased, drug, treated), respectively. **(E)** Union of top 10 targets predicted by PDGrapher in lung, breast, and prostate cancer. The color intensity and size of the bubbles represent the number of evidence sources and the association scores for each type of evidence, respectively. Red, blue, and green dots stand for breast, lung, and prostate cancer, respectively. Symbol “E:” indicates evidence. See details of the scoring system in Supplementary Note 2. **(F)** Predicted targets from PDGrapher in five ranges, 1–10, 11–20, 51–60, 101–110, 501–510, and 1001–1010, on the predicted target list for lung cancer (A549). The color intensity and size of the bubbles represent the number of evidence sources and the global scores of targets from OpenTarget, respectively.

In addition to experiments with random and leave-cell-out splitting, we conducted experiments to examine PDGrapher ‘s ability to discover the targets of unseen approved drugs in training set for chemical cell lines with healthy data available (Chemical-PPI-Lung-A549, Chemical-PPI-Breast-MCF7, Chemical-PPI-Breast-MDAMB231, Chemical-PPI-Breast-BT20, Chemical-PPI-Prostate-PC3, Chemical-PPI-Prostate-VCAP). PDGrapher was used to predict gene targets to shift these diseased cell lines into their healthy states. Figure 5A shows the recovery of targets of FDA-approved drugs for varying values of K (K represents the number of predicted target genes considered in the predicted ranked list), indicating that PDGrapher can identify cancer drug targets among the top predictions.

We analyzed lung cancer by comparing the targets predicted by PDGrapher from lung cancer cell lines with the targets of candidate drugs currently in clinical development, curated from the Open Targets platform [41]. This allowed us to evaluate PDGrapher ‘s ability to predict combinatorial chemical perturbagens. Specifically, we compared the top 10 predicted targets for the A549 lung cancer cell line from PDGrapher with ten randomly selected genes. The results showed that the Open Targets scores and the number of supporting resources for the predicted targets were significantly higher than those for the random genes (Figure S15). Using a cutoff score of 0.5, 8 out of 10 predicted targets had evidence supporting their association with lung cancer, compared to only 2 out of 10 in the random set. Four drugs, tacedinaline (DrugBank ID: DB12291 and clinical trial ID: NCT00005093), selpercatinib (DrugBank ID: DB15685), pralsetinib (DrugBank ID: DB15822), and dexmedetomidine (DrugBank ID: DB00633 and [42]), targeting on these predicted genes were not included in the training set but have been identified as potential treatments for non-small cell lung cancer.

We then evaluated PDGrapher’s predictions by examining FDA-approved drugs that were not present in the training set of PDGrapher. Specifically, we assessed PDGrapher’s performance using the Chemical-PPI-Lung-A549 dataset, focusing initially on pralsetinib, a targeted cancer therapy primarily used to treat non-small cell lung cancer (NSCLC) [43]. Pralsetinib is a selective RET kinase inhibitor designed to block the activity of RET proteins that have become aberrantly active due to mutations or fusions. Pralsetinib is known to target 11 key proteins: RET, DDR1, NTRK3, FLT3, JAK1, JAK2, NTRK1, KDR, PDGFRB, FGFR1, and FGFR2 [44]. RET, the primary target of pralsetinib, was ranked 11th out of 10,716 genes in the predicted list. Half of these targets (5 out of 11) were ranked within the top 100 predicted targets by PDGrapher, including KDR (ranked at 3), FLT3 (ranked at 10), RET (ranked at 11), PDGFRB (ranked at 14), and FGFR2 (ranked at 81). This substantial overlap highlights the potential of the candidate targets identified by PDGrapher for pralsetinib-based lung cancer treatment, given that pralsetinib was not included in the training set of PDGrapher.

Next, we examined KDR as a therapeutic target for lung cancer. KDR, also known as VEGFR-2, has been identified as a significant therapeutic target in A549 lung adenocarcinoma cells. These cells express VEGFR-2 at both mRNA and protein levels, facilitating autocrine signaling that promotes tumor cell survival and proliferation [28]. Activation of VEGFR-2 enhances tumor angiogenesis and growth by upregulating oncogenic factors such as Enhancer of Zeste Homolog 2 (EZH2), which is associated with increased cell proliferation and migration. Inhibiting VEGFR-2 has demonstrated promising therapeutic effects, including reduced cell proliferation and induced apoptosis. For instance, VEGFR-2 inhibitors have been shown to decrease the malignant potential of lung adenocarcinoma cells by downregulating EZH2 expression and increasing sensitivity to chemotherapy [29]. These findings underscore the importance of VEGFR-2 as a therapeutic target in A549 lung adenocarcinoma cells, highlighting its role in tumor progression and the potential benefits of its inhibition in cancer treatment strategies. Importantly, PDGrapher has successfully identified KDR among the top 20 predicted targets in chemical-PPI-lung-A549, validating its precision in detecting key therapeutic targets for lung cancer.

### PDGrapher predicts promising combinations of drug targets for lung cancer.

Given that Open Targets offers more comprehensive evidence for targets currently under development, we conducted a second series of case studies using Open Targets data to evaluate PDGrapher capability to identify candidate therapeutic targets and drugs. This analysis aims to identify targets for lung cancer. Figure 5E presents a bubble graph that illustrates the union of the top 10 predicted targets to transition cell states from diseased to healthy in the six cell lines of three types of cancer that have available healthy controls. In the plot, the color intensity and size of the bubbles represent the number of evidence sources and the association scores for each type of evidence, respectively. Most predicted targets are supported by drugs, pathology and systemic biology, and somatic mutation databases, which were considered strong evidence sources. Two unique targets, TOP2A and CDK2, are predicted exclusively for the lung cancer cell line (Figure S16AB). TOP2A is ranked as the top predicted target by PDGrapher. This gene encodes a crucial decatenating enzyme that alters DNA topology by binding to two double-stranded DNA molecules, introducing a double-strand break, passing the intact strand through the break, and disciplining the broken strand. This mechanism is vital for DNA replication and repair processes. TOP2A could be a potential therapeutic target for anti-metastatic therapy of nonsmall cell lung cancer, since it promotes metastasis of NSCLC by stimulating the canonical Wnt signaling pathway and inducing EMT [45]. Using the predicted target of TOP2A, PDGrapher then identified three drugs, aldoxorubicin, vosaroxin, and doxorubicin hydrochloride, as candidate drugs. These drugs were not part of the training dataset of PDGrapher and are in the early stages of clinical development: aldoxorubicin and vosaroxin are in Phase II trials (ClinicalTrials.gov); doxorubicin hydrochloride is in Phase I but has been shown to improve survival in patients with metastatic or surgically unresectable uterine or soft tissue leiomyosarcoma [46]. Given that PDGrapher can rank all genes based on PPI or GRN data, we assessed two key questions: whether top-ranked genes have stronger evidence from Open Targets compared to lower-ranked genes, and what rank threshold should be used to identify reliably predicted genes. Figure 5F shows the number of sources of evidence and the global scores for the predicted target genes within the rank ranges of 1–10, 11–20, 51–60, 101–10, 501–510 and 1001–1010 for lung cancer (Chemical-PPI-Lung-A549). The analysis revealed a clear trend: both the number of supporting evidence sources and global scores decrease with increasing rank, validating the predictive accuracy of PDGrapher. Most targets ranked within the top 100 have strong evidence from Open Targets, indicating that a rank threshold of 100 could serve as a cut-off for selecting candidate targets.

### Training PDGrapher models.

We conduct an ablation study to evaluate the components of PDGrapher’s objective function using the chemical datasets. We train PDGrapher under three configurations: with only the cycle loss (PDGrapher-Cycle), only the supervision loss (PDGrapher-Super), and both losses combined (PDGrapher-SuperCycle). The experiments are performed in the random splitting setting across all nine PPI chemical datasets. We assess performance using several metrics, including the proportion of samples with partially accurate predictions (Figure 5C), nDCG, recall values (Figure S10), and strength of evidence (Figure S17). The results show that PDGrapher-Super achieves the highest performance in predicting correct perturbagens but performs the worst in reconstructing treated samples. In contrast, PDGrapher-Cycle performs poorly in identifying correct perturbagens but shows improved performance in predicting (reconstructing) held-out treated samples. PDGrapher-SuperCycle (the configuration used throughout this study) strikes a balance between these two objectives, achieving competitive performance in predicting therapeutic genes while demonstrating the best performance in reconstructing treated samples from diseased samples after intervening on the predicted genes. This makes PDGrapher-SuperCycle the most effective choice for balancing accuracy in perturbagen prediction with reconstruction fidelity. The findings demonstrate that the supervision loss is crucial to PDGrapher ‘s overall performance. The PDGrapher-Cycle model consistently underperforms in all cell lines and metrics. Although PDGrapher-Super often excels in ranking performance, including cycle loss (in PDGrapher-SuperCycle) proves its value by moderately improving top prediction metrics such as recall@1 and recall@10. Besides, when healthy control data is available, the top prediction of PDGrapher-SuperCycle demonstrates stronger strength of evidence compared to those of PDGrapher-Super in more than half (4/6) of the cell lines (Figure S17). We chose PDGrapher-SuperCycle for this work because it provides accurate target gene predictions from the top-ranked genes in the predicted list and bases its predictions on the changes they would induce in diseased samples.

Recognizing the role of biological pathways in disease phenotypes, PDGrapher-SuperCycle can identify alternative gene targets within these pathways that may produce similar phenotypic outcomes. The organization of genes within biological pathways, where each gene contributes to specific biochemical processes or signaling cascades, allows perturbations in different genes to yield analogous effects [47]. This pathway-based interconnectivity implies that targeting different genes within a pathway can achieve therapeutic outcomes, as these genes collectively influence cellular functions and phenotypic states [48]. Although PDGrapher-SuperCycle shows slightly lower performance than PDGrapher-Super in pinpointing targets (Figure 5C), it excels in identifying sets of gene targets capable of transitioning cell states from diseased to treated conditions (Figure S10 B and S17).

We conducted sensitivity analyses for PDGrapher using five PPI networks constructed with varying edge confidence cutoffs. The PPI network was obtained from STRING (https://string-db.org/) [49], which assigns a confidence score to each edge. To create networks with different levels of confidence, we filter edges based on the quantiles 0.1, 0.2, 0.3, 0.4, and 0.5 of the confidence scores, resulting in five networks with decreasing numbers of edges. PDGrapher performs robustly at all levels of confidence in PPIs (Figure 5B). For this analysis, we selected two cell lines: Chemical-PPI-Breast-MDAMB231 and Genetic-PPI-Breast-MCF7. The LINCS perturbation data for each cell line were processed using the five PPI networks (Online Methods). We trained PDGrapher with 1, 2, and 3 GNN layers, selecting the best configuration based on the performance of the validation set. As shown in Figure S11, PDGrapher maintains stable performance on both chemical and genetic perturbations, even as an increasing number of edges are removed from the PPI networks.

We created two sparse PPI networks using different edge removal strategies and one synthetic gene expression dataset with increasing levels of latent confounders. We applied two edge removal strategies to the PPI network: removing increasing numbers of either bridge edges or random edges. The third dataset introduces latent confounders in the gene expression data. Details of the data generation process are provided in the Online Methods. PDGrapher demonstrated stable performance in perturbagen prediction, with only a slight decrease in performance as stronger confounders were introduced (Figure S12). Results from the edge removal experiments indicate that while bridge edges are structurally critical, their limited number in the PPI graph reduces their overall impact on model predictions. In contrast, the removal of random edges, which include both high-confidence and redundant connections, has a more pronounced effect on performance, highlighting the model’s sensitivity to network perturbations (Figure S13 and S14).

Some cell lines lack associated healthy control samples from disease-relevant tissues and cell types. These cell lines contain only treatment intervention data (diseased cell state, drug, treated cell state) without disease intervention data (healthy cell state, disease mutations, diseased cell state) for model training and inference. The response prediction and perturbagen discovery modules in PDGrapher can be trained exclusively on treatment intervention data. For cell lines with healthy controls, we trained the response prediction module using both intervention datasets. For cell lines without healthy controls, we trained PDGrapher using only treatment intervention data. To evaluate PDGrapher in the absence of healthy controls, we trained the model in settings with and without disease intervention data in six chemical cell lines (Chemical-PPI-Lung-A549, Chemical-PPI-Breast-MCF7, Chemical-PPI-Breast-MDAMB231, Chemical-PPI-Breast-BT20, Chemical-PPI-Prostate-PC3, Chemical-PPI-Prostate-VCAP) and three genetic cell lines (Genetic-PPI-Lung-A549, Genetic-PPI-Breast-MCF7, Genetic-PPI-Prostate-PC3) for which healthy control data were available. The results indicate that the two versions of PDGrapher perform consistently in different cell types and data types (chemical and genetic) (Figure 5D, Figure S8, and S9). In half of the cell lines (4/9), the model trained without disease intervention data outperformed the model trained with such data, demonstrating the weak dependency of PDGrapher on healthy control data, suggesting that PDGrapher can perform well even in the absence of healthy control data.

## Discussion

We formulate phenotype-driven lead discovery as the combinatorial prediction of therapeutic drug targets. Given a diseased sample, the objective is to identify genes that a genetic or chemical perturbagen should target to reverse disease effects, shifting the sample to a treated state that is distributionally equivalent to a healthy state. This problem involves predicting a combination of gene targets and is therefore framed as a combinatorial prediction of therapeutic targets. To address this problem, we introduce PDGrapher. Using a diseased cell state represented as a gene expression signature and a proxy causal graph of gene-gene interactions, PDGrapher predicts candidate target genes to transition cells to the desired treated state. PDGrapher consists of two modules: a perturbagen discovery module that proposes a set of therapeutic targets based on the diseased and treated states, and a response prediction module that evaluates the effect of applying the predicted perturbagen to the diseased state. Both modules are GNN models operating on gene-gene networks, which approximate noisy causal graphs. We use PPI networks and GRNs as two representations of these noisy causal graphs. PDGrapher predicts perturbagens that shift gene expression from diseased to treated states across two evaluation settings and eight datasets involving genetic and chemical interventions. Unlike alternative response prediction methods, which rely on indirect prediction to identify perturbagens, PDGrapher directly selects candidate gene targets to achieve the desired transformation [36,37,50–52].

The potential of PDGrapher is to improve the design of therapeutic leads and broaden the search space for perturbagens. PDGrapher leverages large datasets of genetic and chemical interventions to find perturbagens as sets of candidate targets to shift cell line gene expressions from diseased to treated states. By flexibly selecting sets of therapeutic targets for intervention rather than on a specific perturbagen, PDGrapher enhances phenotype-driven lead discovery. PDGrapher’s approach in identifying therapeutic targets holds promise for personalized therapies, as it can allow the tailoring of treatments based on individual gene expression profiles. PDGrapher’s capacity to output multiple genes is highly relevant for diseases in which dependencies involving multiple genes influence the efficacy and safety of treatment.

PDGrapher operates under the assumption that there are no unobserved confounders, a stringent condition that is challenging to validate empirically. Future work could focus on reevaluating and relaxing this assumption. Another limitation lies in the reliance on PPIs and GRNs as proxies for causal gene networks, as these networks are inherently noisy and incomplete [53–55]. PDGrapher posits that representation learning can overcome incomplete causal graph approximations. A valuable research direction is to theoretically examine the impact of such approximations, focusing on how they influence the accuracy and reliability of predicted likelihoods. Such analyses could uncover high-level causal variables with therapeutic effects from low-level observations and contribute to reconciling structural causality and representation learning approaches, which generally lack any causal understanding [56]. We performed two experiments to evaluate the robustness of PDGrapher. First, we tested PDGrapher on a PPI network with weighted edges, systematically removing increasing proportions of edges to assess its performance under data degradation. Second, we applied PDGrapher to synthetic datasets with varying levels of missing components in the graph and confounding factors in gene expression data. In both experiments, PDGrapher consistently maintained stable performance.

Phenotype-driven drug discovery using PDGrapher faces certain limitations, one of which is its reliance on transcriptomic data. Although transcriptomics is broadly applicable, including other data modalities, such as cell morphology screens, could produce more comprehensive models. Cell morphology screens, including cell painting, capture cellular responses by staining organelles and cytoskeletal components, generating image profiles that capture the effects of genetic or chemical perturbations [57, 58]. These screens allow identification of phenotypic signatures that correlate with compound activity, mechanisms of action, and potential off-target effects. The recent release of the JUMP Cell Painting dataset [59] exemplifies how high-content morphological profiling can complement databases such as CMap and LINCS, creating integrated datasets for phenotype-driven discovery. By integrating multimodal data, including phenotypic layers based on transcriptomic and image data, it becomes possible to uncover more comprehensive patterns of compound effects [60]. Such an integration would expand the scope of PDGrapher, enabling it to capture broader mechanistic insights and support more effective therapeutic discovery efforts [61,62]. A limitation of our study is the use of NL20 as a control cell line for A549 [63–65]. Although NL20 is a normal human bronchial epithelial cell line and A549 is a human lung carcinoma cell line derived from the alveolar region, the two cell lines differ in anatomical origin and molecular characteristics. This mismatch could introduce biases in comparative analyses due to variations in baseline gene expression profiles and cellular behaviors. To mitigate this concern, we evaluate PDGrapher’s performance across datasets with and without healthy control data. PDGrapher performs consistently regardless of the inclusion of healthy controls, indicating that its predictions are robust to the absence of matched control cells. In our ablation study, we found that incorporating cycle loss improved PDGrapher’s performance in top target predictions for 5 out of 9 cell lines. Based on this improvement, we included the cycle loss in all experiments. Cycle loss plays a role in maintaining the robustness and biological relevance of the model predictions. It achieves this by training the model to predict drug targets that transition from state A to state B by converting diseased gene expression to treated or healthy gene expression, and then reconstructing state B from state A and the predicted targets by predicting response gene expression B given diseased gene expression A and a perturbagen with a set of targets. This bidirectional approach enforces the fidelity of predicted targets as they must contain sufficient information to reconstruct state B from state A. Cycle loss also acts as a regularizer, penalizing discrepancies between the original input and its reconstruction [66].

PDGrapher is a graph neural network approach for combinatorial prediction of perturbations that transition diseased cells to treated states. By leveraging causal reasoning and representation learning on gene networks, PDGrapher identifies perturbagens necessary to achieve specific phenotypic changes. This approach enables the direct prediction of therapeutic targets that can reverse disease phenotypes, bypassing the need for exhaustive response simulations across large perturbation libraries. Its design and evaluation provide a foundation for future advances in the phenotype-based modeling of therapeutic perturbations by improving the precision and scalability of methods to predict perturbations in biological contexts.

## Online Methods

### Datasets

We compiled and processed six data sources and two additional biological information repositories. The data sources include protein-protein interactions (PPI), gene expression in healthy and diseased cell lines, gene expression in diseased cell lines upon chemical or genetic interventions, phenotype-associated genes, drug targets, and drug indications. The following describes the data sources and preprocessing steps.

#### Human protein-protein interaction network.

We built a PPI network by aggregating proteins and protein-protein interactions from BIOGRID [67] (accessed in March 2022), HuRI [68], and Menche et al. [48] In this graph, nodes represent human proteins, and edges exist between nodes if there is physical interaction between proteins. We downloaded a gene ID mapping file from the HUGO Gene Nomenclature Committee. Using this file, we mapped proteins in BIOGRID and Menche et al. [48] from Entrez Gene ID [69] to HUGO Gene Nomenclature Committee ID [70], and proteins in HuRI from Ensembl Gene ID [71] to HUGO Gene Nomenclature Committee ID [70]. Our final PPI comprises the union of nodes and edges, resulting in a graph with 15,742 nodes and 222,498 undirected edges.

#### Human protein-protein interaction network for sensitivity analyses.

To test the sensitivity of PDGrapher on protein-protein interactions, we utilized data from STRING (string-db.org), which provides a confidence score for each edge. The specific dataset used was “9606.protein.physical.links.detailed.v12.0.txt.gz” for Homo sapiens. We filtered the edges by using the 0.1, 0.2, 0.3, 0.4, and 0.5 quantiles of the confidence scores as cutoffs, resulting in five PPI networks with 625,818, 582,305, 516,683, 443,051, and 296,451 edges, respectively. Subsequently, we filtered these PPIs to retain only the nodes (proteins) that were present in the gene expression data.

#### Gene expression data.

We downloaded the Library of Integrated Network-Based Cellular Signatures (LINCS [72]) level 3 gene expression data from https://clue.io/releases/data-dashboard (accessed in February 2022). Level 3 data consists of quantile-normalized samples across each plate and is appropriate for cross-plate analyses. LINCS contains measurements of gene expression for 12,327 genes after genetic and chemical interventions. There are 387,317 samples upon CRISPR genetic interventions (treated samples), with 5,156 unique knocked-out genes in 27 cell lines. There are an average of 17.18 replicates per cell line-knocked-out gene pair. The number of unique genes knocked out in each cell line varies from 1 to 5,114, with an average of 2,042.14 unique genes knocked out per cell line.

Control data for CRISPR interventions, that is, diseased samples, are genetic interventions that do not contain a gene-specific sequence or whose gene-specific sequence targets a gene not expressed in the human genome. There are 47,781 diseased samples in 50 cell lines. The number of diseased samples for each cell line varies from 1 to 6,890, with an average of 955.62 diseased samples per cell line.

There are 1,313,292 samples after chemical interventions (treated samples), with 31,234 unique compounds in 229 cell lines. There is an average of 7.96 replicates per cell line-compound pair. The number of compounds tested in each cell line varies from 1 to 19,509, with an average of 719.69 unique compounds tested per cell line. Drugs are administered at different doses and measured at varying time points after treatment. On average, there are 2.73 different doses per compound-cell line pair, with a minimum of 1 and a maximum of 26 different doses. On average, gene expression is measured at 1.25 time points per compound-cell line pair, with a minimum of 1 and a maximum of 13 different time points.

Control data for chemical interventions, that is, diseased samples, are treated with a vehicle (dimethyl sulfoxide). There are 76,795 diseased samples across 226 cell lines. The number of diseased samples for each cell line varies from 1 to 7,336, with an average of 339.80 diseased samples per cell line. On average, gene expression of diseased samples is measured at 1.4 time points, with a minimum of 1 and a maximum of 5 different time points.

We restricted our analysis to a subset of LINCS cell lines to ensure sufficient perturbational coverage and the inclusion of healthy cell line counterparts. For chemical perturbagens, we first extracted cell lines with “trt_cp” perturbation types and no recorded “failure_mode”, and then filtered out any cell lines with fewer than 1,000 treated samples. Next, we applied the following selection criteria: (1) we selected those that had healthy counterparts available, yielding six cell lines, (2) from the remaining cell lines lacking healthy counterparts, we selected those with more than 15,000 treated samples, (3) of all remaining cell lines, we excluded those which were not found in COSMIC Cell Line Project. Table S11 contains all cell lines that have more than or equal than 1,000 treated samples and their corresponding criteria for exclusion or inclusion. For genetic perturbagens, we first extracted cell lines with “trt_xpr” perturbation types and no recorded “failure_mode”, and then filtered out any cell lines with fewer than 5,000 treated samples. Next, we applied the following selection criteria: (1) we selected those with healthy counterparts available, resulting in three cell lines; (2) from the remaining cell lines lacking healthy counterparts, we selected those with more than 15,000 treated samples. Table S12 contains all cell lines that have more or equal than 5,000 treated samples and their corresponding reason for exclusion or inclusion.

To find healthy cell line counterparts, we extracted all cell lines with the “Unknown” tumor phase in the downloaded LINCS dataset (N=145). Then, we filtered the cell lines by tissue type. To find the exact match to diseased cell lines, we performed a manual literature search to confirm their experimental use as healthy counterparts. We extracted healthy counterparts for three of the ten diseased cell lines: cell line NL20 as the healthy counterpart for A549, cell line MCF10A as the healthy counterpart for MCF7, and cell line RWPE1 as the healthy counterpart for PC3.

We acknowledge that NL20 is a normal human bronchial epithelial cell line, while A549 is a human lung carcinoma cell line derived from a tumor in the alveolar region. These cell lines are not perfectly matched. However, without a more closely matched control cell line for lung cancer research, we have opted to use NL20 as a control for A549.

Genetic interventions correspond to gene experiment knockouts in which the gene expression of the knocked-out gene after the intervention is zero. Chemical interventions correspond to small molecule drugs, each targeting one or more proteins. Chemical interventions were performed at different dose levels and measured at different time points. We included replicates measured at all time points and doses. For each cell line and condition (healthy, diseased, and treated), we log-normalized the level 3 gene expression data. We applied a min-max normalization to transform gene expression values into the range [0*,*1] following established practices in the field.

We match genes in LINCS to proteins in our PPI using the HUGO Gene Nomenclature Committee ID [70], resulting in 10,716 overlapping genes and 151,839 undirected edges. Furthermore, we excluded treated samples from our datasets whose targeted genes were not included in the PPI.

We have healthy, diseased, and treated gene expression samples for each cell line treated with several genetic or chemical perturbagens (Table 1 and 2). For healthy counterparts, samples with the corresponding treatment (“vector” for genetic perturbagens, and “vehicle” for chemical perturbagens) are not available; therefore, we use the closest possible (see “Sample category” in Table 1 and 2.

#### Gene regulatory networks.

We computed a gene regulatory network (GRN) for each diseased cell line in each condition (genetic and chemical datasets), using the GENIE3 [73] algorithm on the gene expression values of each diseased cell line. We filtered genes in our gene expression dataset (LINCS) to contain only those in the PPI before running the GRN algorithm for consistency between the PPI and GRNs. GENIE3, introduced in 2010, won the Dialogue for Reverse Engineering Assessments and Methods 4 (DREAM4) challenge [74], which evaluates the success of GRN inference algorithms on benchmarks of simulated data. GENIE3 was introduced in open source software for bioinformatics bioconductor [75] and is often used for GRN generation [76–79]. It is a model based on an ensemble of regression trees and requires as input a matrix of gene expression levels under various conditions. This expression data are multifactorial. This means that they represent expression levels resulting from a perturbation over a set of genes rather than from a targeted experiment. Multifactorial expression can be obtained as samples from different patients or other biological systems. Therefore, cell line diseased samples are closest to the ideal input data for GENIE3. GENIE3 produces a directed graph that represents regulatory interactions between genes and genes. This is achieved by assigning weights to regulatory links and maximizing weights for more significant links. Then a significance threshold is used to determine which links are substantial enough to be predicted as a regulatory link. We adopted the threshold to generate GRNs with close network density as the PPI from STRING, which was achieved by keeping about 500,000 directed edges.

#### Disease-gene information.

We extracted disease-associated genes from COSMIC [80] (Accessed in September 2022) in addition to expert-curated genes available at https://cancer.sanger.ac.uk/cosmic/curation (Accessed in March 2024). Genes were represented using the HUGO Gene Nomenclature Committee ID. For each cell line in our dataset that has disease intervention data (see Disease intervention data header), we extracted cancer-causing mutations as the list of genes with “Verified” *Mutation verification status* in COSMIC that are also included in the list of genes curated by experts. The mapping of the resulting genes to our list of genes in the PPI produced eight disease-associated genes for the lung cancer cell line A549, nine disease-associated genes for breast cancer cell line MCF7, one disease-associated gene for prostate cancer cell line PC3, two disease-associated genes for prostate cancer cell line VCAP, six disease-associated genes for breast cancer cell line MDAMB231, and eight disease-associated genes for breast cancer cell line BT20.

#### Drug-target information.

We retrieved drug-target information from DrugBank [81] (accessed in November 2022). We extracted drug names and synonyms, chemical identifiers, drug-gene targets, and all available synonyms for each drug target. Only the nominal targets, genes that produce proteins to which the drug physically interacts, were considered when processing the DrugBank data. Genes or gene products involved in the mechanism of action (MoA) of a drug were excluded and not retained for further analysis. We mapped drugs in DrugBank with chemical perturbagens in LINCS using InChI Key [82], resulting in 1,522 out of 31,234 unique LINCS compounds mapped to DrugBank with information about at least one target. We mapped drug targets to our PPI network using the HUGO Gene Nomenclature Committee ID, excluding any drug target that was not mapped. Chemical interventions target multiple genes, with a minimum of 1, a maximum of 300, and an average of 2.44 targets per compound.

#### Cancer drug and target information.

We extracted the list of cancer drugs by cancer type from NCI (https://www.cancer.gov/about-cancer/treatment/types/targeted-therapies/approved-drug-list; Accessed in July 2024). We mapped drug names to DrugBank to obtain cancer drug-gene targets. In total, there are 24 drugs associated with breast cancer (cell lines MCF7, MDAMB231, and BT20), 30 drugs associated with lung cancer (cell line A549), 11 drugs associated with prostate cancer (cell lines PC3 and VCAP), 13 drugs associated with colon cancer (cell line HT29), 18 drugs associated with skin cancer (cell line A375), three drugs associated with cervical cancer (cell line HELA), five drugs associated with ovarian cancer (cell line ES2), four drugs associated with head and neck cancer (cell line BICR6), five drugs associated with pancreatic cancer (cell line YAPC), five drugs associated with stomach cancer (cell line AGS), and six drugs associated with brain cancer (cell line U251MG).

#### Disease intervention data.

Disease intervention datasets consist of gene expression measurements of healthy cell lines, disease-associated genes, and gene expression measurements of diseased cell lines. Gene expression samples from healthy and diseased cell lines were retrieved from LINCS [72], and disease-associated genes were retrieved from COSMIC [80], as detailed previously. Each dataset $𝒯=\left\{T_{1},\ldots ,{\;T}_{M}\right\}$ is a collection of paired healthy-diseased cell lines where in each sample $T=<x^{h},𝒰,x^{d}>$, $x^{h}$ corresponds to gene expression values of the healthy cell line, set 𝒰 is comprised by a randomized subset of disease-associated genes, and $x^{d}$ corresponds to gene expression values of diseased cell lines (that is, upon mutations on genes in 𝒰). To select the randomized set of disease-associated genes, we first choose a proportion p∈{0.25,0.50,0.75,1}, and then select N disease-associated genes at random where N is the proportion multiplied by the total number of disease-associated genes. Given that more diseased samples are available than healthy samples (see Table 1 and 2) when building the triplets, we select a random sample from the set of healthy samples and, therefore, have non-unique healthy samples during training. In total, we built three genetic disease intervention datasets and six chemical disease intervention datasets. The first genetic disease intervention dataset is comprised of gene expression of healthy cell line MCF10A, breast cancer mutations, and gene expression of breast cancer cell line MCF7; the second is comprised of gene expression of healthy cell line NL20, lung cancer mutations, and gene expression of lung cancer cell line A549; and the third is comprised of gene expression of healthy cell line RWPE1, prostate cancer mutations, and gene expression of prostate cancer cell line PC3. The first three chemical disease intervention datasets are comprised of gene expression of healthy cell line MCF10A, breast cancer mutations, and gene expression of breast cancer cell line MCF7, MDAMB231, and BT20; the fourth comprised of gene expression of healthy cell line NL20, lung cancer mutations, and gene expression of lung cancer cell line A549; the fifth and sixth comprised of gene expression of healthy cell line RWPE1, prostate cancer mutations, and gene expression of prostate cancer cell line PC3 and VCAP.

#### Treatment intervention data - genetic.

Genetic treatment intervention datasets consist of singlegene knockout experiments using CRISPR / Cas9-mediated gene knockout. The genetic treatment intervention data include measurements of gene expression of diseased cell lines, single knocked-out genes, and measurements of gene expression of treated cell lines. Gene expression samples from diseased and treated cell lines and knocked-out genes were retrieved from LINCS [72]. Each dataset $𝒯=\left\{T_{1},\ldots ,{\;T}_{M}\right\}$ is a collection of paired diseased-treated cell lines where in each sample $T=<x^{d},{𝒰}^{\prime },x^{t}>$, $x^{d}$ corresponds to gene expression values of the diseased cell line, set ${𝒰}^{\prime }$ is comprised by the knocked-out gene, and $x^{t}$ corresponds to gene expression values of treated cell lines (that is, upon knocking-out the gene in ${𝒰}^{\prime }$). Given that more treated samples are available than diseased samples (see Table 1) when building the triplets, we select a random sample from the set of diseased samples and, therefore, have non-unique diseased samples during training. In total, we built ten datasets of treatment interventions: A549, MCF7, PC3, A375, HT29, ES2, BICR6, YAPC, AGS, and U251MG. They are comprised of gene expression of diseased cells, knocked-out genes, and gene expression of treated cells. Find more details on data compilation and processing in previous subsections.

#### Treatment intervention data - chemical.

Chemical treatment intervention datasets consist of chemical compound treatment experiments. The chemical treatment intervention data include measurements of gene expression of diseased cell lines, drug targets of chemical compounds, and measurements of gene expression of treated cell lines. Gene expression samples of diseased and treated cell lines were retrieved from LINCS, and chemical compound targets were retrieved from DrugBank, as detailed previously. Each dataset $𝒯=\left\{T_{1},\ldots ,{\;T}_{M}\right\}$ is a collection of paired diseased-treated cell lines where in each sample $T=<x^{d},{𝒰}^{\prime },x^{t}>$, $x^{d}$ corresponds to gene expression values of the diseased cell line, set ${𝒰}^{\prime }$ is comprised by the chemical compound targets, and $x^{t}$ correspond to gene expression values of treated cell lines (that is, upon treated with the chemical perturbagen targeting genes in ${𝒰}^{\prime }$). Given that more treated samples are available than diseased samples (see Table 2) when building the triplets, we select a random sample from the set of diseased samples and, therefore, have non-unique diseased samples during training. In total, we built nine datasets of treatment interventions: A549, MCF7, PC3, VCAP, MDAMB231, BT20, HT29, A375, and HELA. They are comprised by gene expression of diseased cells, drug targets, and gene expression of treated cells.

### Related work

#### Learning optimal interventions.

The problem of learning interventions to achieve a desired state has gained interest in recent years. Recent research formulates this problem as the finding of optimal interventions to optimize an associated outcome [83–86]. These works offer varied approaches. For example, Mueller et al. [83] aim to learn an intervention policy defined by a covariate transformation that produces the largest post-intervention improvement with high uncertainty. Pacchiano et al. [84] formalize the task as a bandit optimization problem in which each bandit’s arm corresponds to a covariate to intervene, and the goal is to recover an almost optimal arm in the least number of arm pulls possible. Mueller et al. [85] and Hie et al. [86] approach the problem of sequence-based data where each sequence is associated with an outcome, and the goal is to find mutations in the input sequence that increase a desired outcome. Other recent works formulate this problem as finding optimal interventions to shift the system to a desired state. Zhang et al. [87, 88] aimed to find an intervention that applied to a distribution helps match a desired distribution. Specifically, given a distribution P over X and a desired distribution Q over X, the goal is to find an optimal matching intervention I such that $P^{I}$ best matches Q under some metric. They address the special case of soft interventions (shift interventions) and use the expectation of distributions as the distance metric.

#### Neural networks and structural causal models (SCMs).

Causal representation learning has been a growing trend in recent years [89]. It aims to combine the strength of traditional causal learning methods with the robust capabilities of deep learning in the face of large and noisy data. Bottlenecks of traditional causal learning methods include unstructured high-dimensional variables, combinatorial optimization problems, unknown intervention, unobserved confounders, selection bias, and estimation bias [89]. There are three areas in which deep learning helps to overcome these bottlenecks [89]. First, in learning causal variables from high-dimensional unstructured data. Second, in learning the causal structure between causal variables, called *causal discovery* within the causal inference literature. Third, it facilitates the inference of interventional and counterfactual queries. Within the last branch, a promising approach aims to join SCMs and neural models to facilitate interventional and counterfactual querying. Parafita et al. put forward the requirements that any DL model should fulfill to approximate causal queries and introduced normalizing causal flows as a specific instantiation [90]. Pawlowski et al. followed a similar approach to introduce a model capable of computing counterfactual queries [91]. Xia et al. approached the problem differently, introducing a Neural Causal Model (NCM), a type of SCM with neural networks as structural equations [92]. Together with the NCM, they introduced an algorithm that provably performs the identification and inference of interventional queries [92]. A follow-up work extended the NCM framework for the identification and inference of counterfactual queries [93]. The concept of NCMs inspires our work by considering the graph in which we operate as a noisy version of a causal graph and our model operating on the graph as a proxy for the structural equations.

#### Interventions in graph neural networks (GNNs).

GNNs are a type of neural model that falls under the umbrella term of geometric deep learning [94–96]. These models use graph-structured data to compute transformed representations useful for downstream predictive tasks. Their ability to operate over graphs makes them especially relevant to NCMs. A recent work by Zecevic et al. [97] explored this connection. It introduced interventional GNNs, a GNN in which interventions are represented through mutilations in the input graph, and interventional inference as GNN computations on the mutilated graph [98]. We borrow this concept in our work and extend the representational capabilities of GNNs by assigning learnable embeddings to input nodes.

## Methods

### Preliminaries.

A calligraphic letter 𝒳 indicates a set, an italic uppercase letter X denotes a graph, uppercase X denotes a matrix, lowercase x denotes a vector, and a monospaced letter X indicates a tuple. Uppercase letter X indicates a random variable, and lowercase letter x indicates its corresponding value; bold uppercase X denotes a set of random variables, and lowercase letter x indicates its corresponding values. We denote P(X) as a probability distribution over a set of random variables X and P(X=x) as the probability of X is equal to the value of x under the distribution P(X). For simplicity, P(X=x) is abbreviated as P(x).

### Problem formulation - combinatorial prediction of therapeutic targets.

Intuitively, given a diseased cell line sample, we would like to predict the set of therapeutic genes that need to be targeted to reverse the effects of disease, that is, the genes that need to be perturbed to shift the cell gene expression state as close as possible to the healthy state. Next, we formalize our problem formulation. Let M=<E,V,ℱ,P(E)> be an SCM associated with causal graph G, where E is a set of exogenous variables affecting the system, V are the system variables, ℱ are structural equations encoding causal relations between variables and P(E) is a probability distribution over exogenous variables. Let $𝒯=\left\{T_{1},\ldots ,{\;T}_{m}\right\}$ be a dataset of paired healthy and diseased samples, where each element is a 3-tuple $T=<v^{h},U,v^{d}>$ with $v^{h}\in [0,\;1{]}^{N}$ being gene expression values of healthy cell line (variable states before perturbation), $V_{U}$ being the disease-causing perturbed variable (gene) set in V, and $v^{d}\in [0,\;1{]}^{N}$ being gene expression values of diseased cell line (variable states after perturbation). Our goal is to find, for each sample $T=<v^{h},U,v^{d}>$, the variable set $U^{\prime }$ with the highest likelihood of shifting variable states from diseased $v^{d}$ to healthy $v^{h}$ state. To increase generality, we refer to the desired variable states as *treated* $\left(v^{t}\right)$. Our goal can then be expressed as:

(1) $${argmax\;}_{U^{\prime }}P^{G^{U}}\left(V=v^{t}|do\left(U^{\prime }\right)\right),$$

where $P^{G^{U}}$ represents the probability on the graph G mutilated by perturbations in variables in U. Under the assumption of no unobserved confounders, the above interventional probability can be expressed as a conditional probability on the mutilated graph $G^{U^{\prime }}$:

(2) $${argmax\;}_{U^{\prime }}P^{G^{U^{\prime }}}\left(V=v^{t}|U^{\prime }\right),$$

which under the causal Markov condition is:

(3) $${argmax\;}_{U^{\prime }}\prod_{i}P\left(V_{i}=v_{i}^{t}|{Pa}_{V_{i}}\right),$$

where ${Pa}_{V_{i}}$ represents parents of variable $V_{i}$ according to graph $G^{U^{\prime }}$ (that is, the mutilated graph upon intervening on variables in $U^{\prime }$). Here, state of a variable $V_{j}\in {\;Pa}_{v_{i}}$ will be equal to an arbitrary value $v_{j}^{\prime }$ if $V_{j}\in U^{\prime }$. Therefore, intervening on the variable set $U^{\prime }$ modifies the graph used to obtain conditional probabilities and determine the state of variables in $U^{\prime }$.

### Problem formulation - representation-learning-based combinatorial prediction of therapeutic targets.

In the previous section, we drew on the SCM framework to introduce a generic formulation for the task of combinatorial prediction of therapeutic targets. Instead of approaching the problem from a purely causal inference perspective, we draw upon representation learning to approximate the queries of interest to address the limiting real-world setting of a noisy and incomplete causal graph. Formulating our problem using the SCM framework allows for explicit modeling of interventions and formulation of interventional queries. Inspired by this principled problem formulation, we next introduce the problem formulation using a representation learning paradigm.

We let G=(𝒱,ℰ) denote a graph with |𝒱|=n nodes and |ℰ| edges, which contains partial information on causal relationships between nodes in 𝒱 and some noisy relationships. We refer to this graph as *proxy causal graph*. Let $𝒯=\left\{T_{1},\ldots ,{\;T}_{M}\right\}$ be a dataset with an individual sample being a 3-tuple $T=<x^{h},𝒰,x^{d}>$ with $x^{h}\in [0,\;1{]}^{n}$ being the node states (attributes) of healthy cell sample (before perturbation), 𝒰 being the set of disease-causing perturbed nodes in 𝒱, and $x^{d}\in [0,\;1{]}^{n}$ being the node states (attributes) of diseased cell sample (after perturbation). We denote by $G^{𝒰}=(𝒱,{ℰ}^{𝒰})$ the graph resulting from the mutilation of edges in G as a result of perturbing nodes in 𝒰 (one graph per perturbagen; we avoid using superindices for simplicity). Here again, we refer to the desired variable states as *treated* $\left(x^{t}\right)$. Our goal is then to learn a function:

(4) $$f:G^{{𝒰}^{\prime }},x^{d},x^{t}\to {argmax\;}_{{𝒰}^{\prime }}P^{G^{{𝒰}^{\prime }}}\left(x=x^{t}|x^{d},{𝒰}^{\prime }\right).$$

That is, given the graph $G^{{𝒰}^{\prime }}$, the diseased $x^{d}$ and treated $x^{t}$ node states, predicts the combinatorial set of nodes ${𝒰}^{\prime }$ that if perturbed have the highest chance of shifting the node states to the treated state $x^{t}$. We note here that $P^{G^{{𝒰}^{\prime }}}$ represents probabilities over graph $G^{𝒰}$ mutilated upon perturbations in nodes in ${𝒰}^{\prime }$. Under Causal Markov Condition, we can factorize $P^{G^{{𝒰}^{\prime }}}$ over graph $G^{{𝒰}^{\prime }}:$

(5) $$f:G^{{𝒰}^{\prime }},x^{d},x^{t}\to {argmax\;}_{{𝒰}^{\prime }}\prod_{i}P\left(x_{i}=x_{i}^{t}|x_{𝒫{𝒜}_{i}}\right),$$

that is, the probability of each node depending only on its parents $𝒫{𝒜}_{i}$ in graph $G^{{𝒰}^{\prime }}$.

We assume (i) real-valued node states, (ii) G is fixed and given, and (iii) atomic and non-atomic perturbagens (intervening on individual nodes or sets of nodes). Given that the value of each node should depend only on its parents in the graph $G^{{𝒰}^{\prime }}$, a message-passing framework appears especially suited to compute the factorized probabilities P.

In the SCM framework, the conditional probabilities in Equation 3 are computed recursively on the graph, each being an expectation over exogenous variables E. Therefore, node states of the previous time point are not necessary. To translate this query into the representation learning realm, we discard the existence of noise variables and directly try to learn a function encoding the transition from an initial state to a desired state. An exhaustive approach to solving Equation 5 would be to search the space of all potential sets of therapeutic targets ${𝒰}^{\prime }$ and score how effective each is in achieving the desired treated state. This is, indeed, how many cell response prediction approaches can be used for perturbagen discovery [22, 23, 99]. However, with moderately sized graphs, this is highly computationally expensive, if not intractable. Instead, we propose to search for potential perturbagens efficiently with a perturbagen discovery module $\left(f_{p}\right)$ and a way to score each potential perturbagen with a response prediction module $\left(f_{r}\right)$.

### Relationship to conventional graph prediction tasks.

Given that the prediction for each variable is dependent only on its parents in a graph, GNNs appear especially suited for this problem. We can formulate the query of interest under a graph representation learning paradigm as: Given a graph G=(𝒱,ℰ), and paired sets of node attributes $𝒳=\left\{X_{1},X_{2},\ldots ,X_{m}\right\}$ and node labels $𝒴=\left\{Y_{1},Y_{2},\ldots ,Y_{m}\right\}$ where each $Y=\left\{y_{1},\ldots ,y_{n}\right\}$, with $y_{i}\in [0,\;1]$, we aim at training a neural message passing architecture that given node attributes $X_{i}$ predicts the corresponding node labels $Y_{i}$. There are, however, differences between our problem formulation and the conventional graph prediction tasks, namely, graph and node classification (summarized in Table 3).

In node classification, a single graph G is paired with node attributes X, and the task is to predict the node labels Y. Our formulation differs in that we have m paired sets of node attributes 𝒳 and labels 𝒴 instead of a single set, yet they are similar in that there is a single graph in which GNNs operate. In graph classification, a set of graphs $𝒢=\left\{G_{1},\ldots ,G_{m}\right\}$ is paired with a set of node attributes $𝒳=\left\{X_{1},X_{2},\ldots ,X_{m}\right\}$ and the task is to predict a label for each graph $Y\;=\left\{y_{1},\ldots ,y_{m}\right\}$. Here, graphs have a varying structure, and both the topological information and node attributes predict graph labels. In our formulation, a single graph is combined with each node attribute $X_{i}$, and the goal is to predict a label for each node, not for the whole graph.

### PDGrapher model.

PDGrapher is an approach for combinatorial prediction of therapeutic targets composed of two modules. First, a perturbagen discovery module $f_{p}$ searches the space of potential gene sets to predict a suitable candidate ${𝒰}^{\prime }$. Next, a response prediction module $f_{r}$ checks the goodness of the predicted set ${𝒰}^{\prime }$, that is, how effective intervening on variables in ${𝒰}^{\prime }$ is to shift node states to the desired treated state $x^{t}$.

$$(1)\;x^{d},x^{t}\overset{f_{p}\;}{\to }{\overset{ˆ}{𝒰}}^{\prime }$$

$$(2)\;x^{d},{\overset{ˆ}{𝒰}}^{\prime }\overset{f_{r}\;}{\to }{\overset{ˆ}{x}}^{t}.$$

### Model optimization.

We optimize our response prediction module $f_{r}$ using cross-entropy loss on known triplets $<x^{h},𝒰,x^{d}>$ and $<x^{d},{𝒰}^{\prime },x^{t}>$:

(6) $${ℒ}_{f_{r}}=CE\left(x^{d},f_{r}\left(x^{h},𝒰\right)\right)+CE\left(x^{t},f_{r}\left(x^{d},{𝒰}^{\prime }\right)\right).$$

We optimize our intervention discovery module $f_{p}$ using a cycle loss such that the response upon a predicted ${𝒰}^{\prime }$ is as close to the desired treated state as possible. In addition, we provide a supervisory signal for predicting ${𝒰}^{\prime }$ in the form of cross-entropy loss as follows:

(7) $${ℒ}_{f_{p}}=CE\left(x^{t},f_{r}\left(x^{d},f_{p}\left(x^{d},x^{t}\right)\right)\right)+CE\left({𝒰}^{\prime },f_{p}\left(x^{d},x^{t}\right)\right)\;\text{(with}\;f_{r}\;\text{frozen).}$$

We train $f_{p}$ and $f_{r}$ in parallel and implement early stopping separately (see *Experimental setup* for more details). Trained modules $f_{p}$ and $f_{r}$ are then used to predict, for each diseased cell sample, which nodes should be perturbed $\left({𝒰}^{\prime }\right)$ to achieve a desired treated state (Figure 1A).

### Response prediction module.

Our response prediction module $f_{r}$ should learn to map pre-perturbagen node values to post-perturbagen node values through learning relationships between connected nodes (equivalent to learning structural equations in SCMs) and propagating the effects of perturbations downstream in the graph (analogous to the recursive nature of query computations in SCMs).

Given a triplet $<x^{h},𝒰,x^{d}>$, we propose a neural model operating on a mutilated graph, $G^{𝒰}$ where the node attributes are the concatenation of $x^{h}$ and $x_{𝒰}^{\prime }$, predicting diseased node values $x^{d}$. Each node i has a two-dimensional attribute vector $d_{i}=\left[x_{i}^{h}\|x_{𝒰}^{\prime }\right]$, where the first element is its gene expression value $x_{i}^{h}$, and the second is a perturbation flag: a binary label indicating whether a perturbation occurs at node i. In practice, we embed each node feature into a high-dimensional continuous space by assigning learnable embeddings to each node based on the value of each input feature dimension. Specifically, for each node, we use the binary perturbation flag to assign a d-dimensional learnable embedding, which is different between nodes but shared across samples for each node. To embed the gene expression value $x_{i}^{h}\in [0,\;1]$, we first calculate thresholds using quantiles to assign the gene expression value into one of the B bins. We use the bin index to assign a d-dimensional learnable embedding, which is different between nodes but shared across samples for each node. To increase our model’s representation power, we concatenate a d-dimensional positional embedding (d-dimensional vector initialized randomly following a normal distribution). Concatenating these three embeddings results in an input node representation of dimensionality 3d. For each node i∈𝒱, an embedding $z_{i}$ is computed using a graph neural network operating on the node’s neighbors’ attributes. The most general formulation of a GNN layer is:

(8) $$h_{i}^{\prime }=\phi \left(h_{i},\underset{j\in {𝒩}^{i}}{⨁}\;\psi \left(h_{i},h_{j}\right)\right),$$

where $h_{i}^{\prime }$ represents the updated information of node i, and $h_{i}$ represents the information of node i in the previous layer, with embedded $d_{i}$ being the input to the first layer. ψ is a *MESSAGE* function, ⨁ an *AGGREGATE* function (permutation-invariant), and ϕ is an *UPDATE* function. We obtain an embedding $z_{i}$ for node i by stacking K GNN layers. The node embedding $z_{i}\in R$ is then passed to a multilayer feedforward neural network to obtain an estimate of the values of the post-perturbation nodes $x^{d}$.

### Perturbation discovery module.

Our perturbagen prediction module $f_{p}$ should learn the nodes in the graph that should be perturbed to shift the node states (attributes) from diseased $x^{d}$ to the desired treated state $x^{t}$. Given a triplet $<x^{d},{𝒰}^{\prime },x^{t}>$, we propose a neural model operating on graph $G^{{𝒰}^{\prime }}$ with node features $x^{d}$ and $x^{t}$ that predicts a ranking for each node where the top P ranked nodes should be predicted as the nodes in ${𝒰}^{\prime }$. Each node i has a two-dimensional attribute vector: $d_{i}=\left[x_{i}^{d}\|x_{i}^{t}\right]$. In practice, we represent these binary features in a continuous space using the same approach as described for our response prediction module $f_{r}$.

For each node i∈𝒱, an embedding $z_{i}$ is computed using a graph neural network operating on the node’s neighbors’ attributes. We obtain an embedding $z_{i}$ for node i by stacking K GNN layers. The node embedding $z_{i}\in R$ is then passed to a multilayer feedforward neural network to predict a real-valued number for node i.

### Model implementation and training.

We implement PDGrapher using PyTorch 1.10.1 [100] and the Torch Geometric 2.0.4 Library [101]. The implemented architecture yields a neural network with the following hyperparameters: number of GNN layers and number of prediction layers. We set the number of prediction layers to two and performed a grid search over the number of GNN layers (1–3 layers). We train our model using a 5 -fold cross-validation strategy and report PDGrapher’s performance resulting from the best-performing hyperparameter setting.

### Further details on statistical analysis

We next outline the evaluation setup, baseline models, and statistical tests used to evaluate PDGrapher. We evaluate the performance of PDGrapher against a set of baselines:

- **Random baseline:** Given a sample $T=<x^{d},{𝒰}^{\prime },x^{t}>$, the random baseline returns N random genes as the prediction of genes in ${𝒰}^{\prime }$, where N is the number of genes in ${𝒰}^{\prime }$.
- **Cancer genes:** Given a sample $T=<x^{d},{𝒰}^{\prime },x^{t}>$, the cancer genes baseline returns the top N genes from an ordered list where the first M genes are disease-associated (cancer-driver genes). The remaining genes are ranked randomly, and N is the number of genes in ${𝒰}^{\prime }$. The processing of cancer genes is described in the section on *Disease-associated genes*.
- **Cancer drug targets:** Given a sample $T=<x^{d},{𝒰}^{\prime },x^{t}>$, the cancer targets baseline returns the top N genes from an ordered list where the first M genes are cancer drug targets and the remaining genes are ranked randomly, and N is the number of genes in ${𝒰}^{\prime }$. The processing of drug target information is described in sections on *Drug targets and Cancer drug and target information*.
- **Perturbed genes:** Given a sample $T=<x^{d},{𝒰}^{\prime },x^{t}>$, the perturbed genes baseline returns the top N genes from an ordered list where the first M genes are all perturbed genes in the training set and the remaining genes are ranked randomly, and N is the number of genes in ${𝒰}^{\prime }$.
- **scGen** [22]: scGen is a widely-used gold-standard latent variable model for response prediction [102–105]. Given a set of observed cell type in control and perturbed state, scGen predicts the response of a new cell type to the perturbagen seen in training. To utilize scGen as a baseline, we first fit it to our LINCS gene expression data for each dataset type to predict response to perturbagens, training one model per perturbagen (chemical or genetic). Then, given a sample of paired diseased-treated cell line states, $T=<x^{d},{𝒰}^{\prime },x^{t}>$, we compute the response of cell line with gene expression $x^{d}$ to all perturbagens. The predicted perturbagen is that whose predicted response is closest to $x^{t}$ in $R^{2}$ score. Since scGen trains one model per perturbagen, it needs an exhaustively long training time for datasets with a large number of perturbagens, especially in the leave-cell-out setting. Therefore, we set the maximum training epochs to 100 and only conducted leave-cell-out tests for one split of data for scGEN.
- **Biolord** [33]: Biolord can predict perturbagen response for both chemical and genetic datasets. We followed the official tutorial from the Biolord GitHub repository: https://github.com/nitzanlab/biolord, using the recommended parameters. To prevent memory and quota errors, we implemented two filtering steps: 1) Instead of storing the entire response gene expression (rGEX) matrix of all input (control) cells for each perturbagen, we only store a vector of the averaged rGEX of the input cells per perturbagen, which is necessary for calculating $R^{2}$ for evaluation. 2) During prediction, if the number of control cells exceeds 10,000, we randomly down-sample the control cells to 10,000. Similar to scGEN, we calculate the responses gene expression $x^{d\prime }$ for all perturbagens and use them to calculate $R^{2}$ to get the rank of predicted perturbagens.
- **ChemCPA** [32]: ChemCPA is specifically designed for chemical perturbation. We followed the official tutorials on GitHub for running this model (https://github.com/theislab/chemCPA), with all parameters set following the authors’ recommendations. Data processing was also conducted using the provided scripts. We constructed drug embedding using RDKit with canonical SMILES sequences, as this is the default setting in the model and the tutorial. Since the original ChemCPA model lacks functionality to obtain the predicted rGEX for each drug (averaging over the dosages), we developed a custom script to perform this task. These predictions were subsequently used for calculating $R^{2}$ to get the rank of predicted perturbagens/targets.
- **GEARS** [34]: GEARS is capable of predicting perturbagen responses for genetic perturbation datasets, specifically for predicting the rGEX to unseen perturbagens. However, it is limited to predicting only those genes that are present in the gene network used as prior knowledge for model training. Additionally, GEARS cannot process perturbagens with only one sample, so we filtered the data accordingly. We followed the official tutorial from the GEARS GitHub repository (https://github.com/snap-stanford/GEARS), using the recommended parameters. After confirming with the authors, we established that GEARS is suitable only for within-cell-line prediction. Consequently, our experiments with GEARS were conducted exclusively within this scenario.
- **CellOT** [27]: CellOT is capable of working with both chemical and genetic datasets. We ran this model by following the official tutorial from GitHub (https://github.com/bunnech/cellot), ensuring that all parameters were set according to the provided guidelines. Due to CellOT’s limitation in processing perturbagens with small sample sizes, we filtered the data to retain only those perturbagens with more than five samples or cells. We then used the predicted response gene expression $x^{d\prime }$ to calculate $R^{2}$ and the predicted perturbagen ranks. Similar to scGEN, CellOT trains one model per perturbagen, which results in an exhaustively long training time for datasets with a large number of perturbagens. This issue becomes even more significant when doing leave-cell-out evaluations. Therefore, for this method, we set the maximum training epochs to 100 and only conduct one split in leave-cell-out tests.

### Dataset splits and evaluation settings.

We evaluate PDGrapher and competing methods on two different settings:

- **Systematic random dataset splits:** Our dataset is split randomly into train and test sets to measure our model performance in an IID setting.
- **Leave-cell-out dataset splits:** To measure model performance on unseen cell lines, we train our model with random splits on one cell line and test on a new cell line. Specifically, for chemical perturbation data, we train a model for each random split per cell line and test it on the entire dataset of the remaining eight cell lines. For genetic data, we train a model for each random split per cell line and test it on the entire dataset of the remaining nine cell lines. For example, with nine cell lines with chemical perturbation (A549, MDAMB231, BT20, VCAP, MCF7, PC3, A375, HT29, and HELA), we conducted an experiment where each split of cell line A549 was used as the training set, and the trained model was tested on the remaining eight cell lines (MDAMB231, BT20, VCAP, MCF7, PC3, A375, HT29, and HELA). Similarly, for cell line MDAMB231, we trained the model on each split of it and tested the model on the other eight cell lines (A549, BT20, VCAP, MCF7, PC3, A375, HT29, and HELA). This process was repeated for all cell lines, providing a comprehensive evaluation of PDGrapher and all competing methods.

### Evaluation setup.

For all dataset split settings, our model is trained using 5-fold cross-validation, and metrics are reported as the average on the test set. Within each fold, we further split the training set into training and validation sets (8:2) to perform early stopping: we train the model on the training set until the validation loss has not decreased at least 10^−5^ for 15 continuous epochs.

### Evaluation metrics.

We report average sample-wise $R^{2}$ score, and average perturbagen-wise $R^{2}$ score to measure performance in the prediction of $x^{t}$. The sample-wise $R^{2}$ score is computed as the square of Pearson correlation between the predicted sample ${\overset{ˆ}{x}}^{t}\in R^{N}$ and real sample $x^{t}\in R^{N}$. The perturbagen-wise $R^{2}$ score is adopted from scGen. It is computed as the square of Pearson correlation of a linear least-squares regression between a set of predicted treated samples ${\overset{ˆ}{X}}^{t}\in R^{N\times S}$ and a set of real treated samples $X^{t}\in R^{N\times S}$ for the same perturbagen. Higher values indicate better performance in predicting the treated sample $x^{t}$ given the diseased sample $x^{d}$ and predicted perturbagen. When evaluating competing methods that cannot predict perturbagen ranks for chemical perturbation data, we first calculate the rank of drugs based on the $R^{2}$ score. We then build a target gene rank from the drug rank by substituting the drugs with their target genes acquired from DrugBank [81] (accessed in November 2022) (see details in the data section). Notably, a single drug can have multiple target genes, which we place in the rank in random order. Since some methods cannot predict unseen drugs, their predicted target gene lists are often short, introducing bias in evaluation. To address this, we shuffle the missing target genes and attach them to the predicted ranks to create a complete rank. For genetic perturbation data, we directly obtain the target gene rank from the results, then attach the shuffled missing genes to the rank.

To evaluate the performance of our model in ranking predicted therapeutic targets, we use the Normalized Discounted Cumulative Gain (nDCG), a widely-used metric in information retrieval adapted for our setting. The raw DCG score is computed by summing the relevance of each correct target based on its rank in the predicted list, with relevance weighted by a logarithmic discount factor to prioritize higher-ranked interventions. The gain function is defined as 1-ranking/N, ensuring that the score reflects the quality of the ranking relative to the total number of nodes in the system. To ensure comparability across datasets or experiments with different numbers of correct interventions, DCG is normalized by the Ideal DCG (IDCG), which represents the maximum possible score for a perfect ranking. This results in nDCG values in the range [0, 1], where higher values indicate better ranking performance and alignment with the ground truth. This metric is particularly suited for our task as it emphasizes the accuracy of top-ranked interventions while accounting for the diminishing importance of lower-ranked predictions.

In addition, we report the proportion of test samples for which the predicted therapeutic targets set has at least one overlapping gene with the ground-truth therapeutic targets set. We also calculated the ratio of correct therapeutic targets that appeared in the top 1, top 10, and top 100 predicted therapeutic targets in the predicted rank, denoted as recall@ 1, recall@10, and recall@100, respectively.

To assess the overall performance across all experiments and metrics, we calculated an aggregated metric, averaging all metric values for each method.

The performance of response prediction is assessed by calculating the $R^{2}$ value between the predicted and actual gene expression responses to a perturbagen. To obtain perturbagen-level data, the response gene expression of all samples subjected to the same perturbagen are averaged. The mechanistic baseline for response prediction involves directly calculating the $R^{2}$ value between the gene expression profiles of the disease (unperturbed) state and the treated (perturbed) state.

### Statistical tests

In the benchmarking experiments, we performed a one-tailed pairwise t-test to evaluate whether PDGrapher significantly outperforms the competing methods. For other experiments, such as ablation studies, we employed a two-tailed t-test to determine whether there is a significant difference in performance between the two models. A significance threshold of 0.05 was used for all tests. We conducted two levels of testing to evaluate the methods: (1) performance comparison within individual training cell lines, assessing how well each method performed in isolation for a specific training cell line (with multiple testing cell lines in leave-cell-out experiments), and (2) performance comparison across the entire experiment, analyzing the overall effectiveness of the methods when considering all cell lines together. P-values of perturbagen discovery and response prediction tests are presented in Table S9 and S10, respectively.

### Ablation studies.

In the ablation study, we evaluated PDGrapher by optimizing it with only the supervision loss (PDGrapher-Super) and with only the cycle loss (PDGrapher-Cycle) across all chemical datasets. We then compared the perturbagen prediction performance of these sub-models with that of PDGrapher (PDGrapher-SuperCycle). To train PDGrapher-Super and PDGrapher-Cycle, for each cell line, we set the number of layers to that which was found optimal for the validation set in the random splitting setting for PDGrapher-SuperCycle.

### Sensitivity studies.

To test the sensitivity of PDGrapher on protein-protein interactions (PPIs), we utilized data from STRING (string-db.org), which provides a confidence score for each edge. The method for acquiring and preprocessing the PPIs from STRING is detailed in the Dataset section. For the sensitivity tests, we selected two cell lines: the chemical dataset MDAMB231 and the genetic dataset MCF7. For each cell line, we processed the data as described above using the five PPI networks. We optimized PDGrapher using 5-fold cross-validation as described in the *Evaluation setup* header and optimized the number of GNN layers using the validation set in each split.

### Synthetic datasets.

We generated three synthetic datasets: **(1) dataset with missing components removing bridge edges:** this dataset is generated by progressively removing bridge edges from the existing PPI network. Bridge edges are those whose removal disconnects parts of the network. We vary the fraction of bridge edges removed in increments (from 0 to 1), and for each fraction, we create a new edge list representing the modified network (Table S5). This process ensures that different levels of network sparsity are introduced, affecting the overall structure and connectivity. We pair these networks with gene expression data from Chemical-PPI-Breast-MDAMB231. **(2) Dataset with missing components removing random edges:** this dataset is generated by progressively removing random edges from the existing PPI network. We vary the fraction of bridge edges removed in increments (from [0, 0.1, … 0.6]), and for each fraction, we create a new edge list representing the modified network. The number of remaining directed edges in the network upon random edge removal are 273,319; 242,912; 212,525; 182,177; 151,811; 121,472. **(3) Dataset with latent confounder noise:** our starting point is the Chemical-PPI-Breast-MDAMB231 dataset. The synthetic datasets were constructed with varying levels of confounding bias introduced into the gene expression data. To simulate latent confounder effects, Gaussian noise with distinct means and variances was progressively added to random subsets of genes. Genes were grouped into 50 predefined subsets, each representing a latent confounder group. For each group, a Gaussian distribution was defined, with the mean drawn randomly from a uniform distribution in the range [0.5, 0.5] and the standard deviation from [0.1, 0.5]. A fraction ([0.2,0.4, 0.6, 0.8,1]) of these subsets was randomly selected for perturbation, and for each gene in these subsets, its expression value was incremented by a value sampled from the respective Gaussian distribution. The perturbed gene expression values were then clamped between 0 and 1 to ensure validity. This strategy ensures that different latent biases are introduced globally to gene expression patterns while maintaining controlled variability. We pair the noisy version of the gene expression data with the global unperturbed PPI network.

### Network proximity between predicted and ground truth perturbagens.

Let 𝒫 be the set of predicted therapeutic targets, ℛ be the set of ground truth therapeutic targets, and spd(p,r) be the shortest-path distance between nodes in P and R. We measure the closest distance between P and R as:

(9) $$d(P,R)=\frac{1}{\left|R||P\right|}\sum_{r\in R}\sum_{p\in P}spd(p,r).$$

As part of our performance analyses, we measure network proximity of PDGrapher and competing methods. We compared the distributions of network proximity values using a Mann-Whitney U test, along with rank-biserial correlation to measure effect size. To assess the uncertainty of effect sizes, we performed bootstrapping with 1,000 resamples to estimate 95% confidence intervals.

## Supplementary Material

Supplement 1

Supplement 2

Supplement 3

Supplement 4

## Figures and Tables

**Table 1:** Table shows several healthy, diseased, and treated samples for lung cancer (A549), breast cancer (MCF7), and prostate cancer (PC3), and only diseased and treated samples for skin cancer (A375), colon cancer (HT29), ovarian cancer (ES2), head and neck cancer (BICR6), pancreatic cancer (YAPC), stomach cancer (AGS), and brain cancer (U251MG) with genetic perturbations.

Cancer type	Cell line	Sample type	N samples	Category	N perturbagens

Lung cancer	A549	healthy	50	vehicle	-
		diseased	4,327	vector	-
		treated	24,255	CRISPR	3,711

Breast cancer	MCF7	healthy	113	untreated	-
		diseased	4,852	vector	-
		treated	18,774	CRISPR	3,090

Prostate cancer	PC3	healthy	185	vector	-
		diseased	6,890	vector	-
		treated	21,229	CRISPR	3,710

Skin cancer	A375	diseased	4,777	vector	-
		treated	21,794	CRISPR	3,709

Colon cancer	HT29	diseased	4,235	vector	-
		treated	20,525	CRISPR	3,706

Ovary cancer	ES2	diseased	1,277	vector	-
		treated	23,708	CRISPR	3,654

Head and Neck	BICR6	diseased	1,362	vector	-
		treated	21,183	CRISPR	3,711

Pancreas cancer	YAPC	diseased	1,275	vector	-
		treated	20,135	CRISPR	3,711

Stomach cancer	AGS	diseased	1,352	vector	-
		treated	21,284	CRISPR	3,712

Brain cancer	U251MG	diseased	1,449	vector	-
		treated	26,323	CRISPR	3,712

**Table 2:** Table shows several healthy, diseased, and treated samples for lung cancer (A549), breast cancer (MCF7, MDAMB231, and BT20), and prostate cancer (PC3 and VCAP), and only diseased and treated samples for cervical cancer (HELA), colon cancer (HT29), and skin cancer (A375) with chemical perturbations.

Cancer type	Cell line	Sample type	N samples	Category	N perturbagens

Lung cancer	A549	healthy	50	vehicle	-
		diseased	5,261	vehicle	-
		treated	23,100	compound	1,041

Breast cancer	MCF7	healthy	2,675	untreated	-
		diseased	7,336	vehicle	-
		treated	35,421	compound	1,154

Breast cancer	MDAMB231	healthy	2,675	untreated	-
		diseased	1,591	vehicle	-
		treated	10,004	compound	526

Breast cancer	BT20	healthy	2,675	untreated	-
		diseased	409	vehicle	-
		treated	1,403	compound	39

Prostate cancer	PC3	healthy	185	vector	-
		diseased	7,202	vehicle	-
		treated	32,555	compound	1,182

Prostate cancer	VCAP	healthy	185	untreated	-
		diseased	3,904	vehicle	-
		treated	7,364	compound	738

Colon cancer	HT29	diseased	4,317	vehicle	-
		treated	19,386	compound	1,053

Skin cancer	A375	diseased	5,165	vehicle	-
		treated	25347	compound	1,093

Cervix cancer	HELA	diseased	2,905	vehicle	-
		treated	20,308	compound	683

**Table 3:** Our problem formulation is similar to conventional node and graph classification tasks, albeit some major differences exist.

Task	Number of graphs	Number of node attribute sets	Label dimensions

Graph Classification	m	m	m × 1 (one for each graph)
Node Classification	1	1	1 × n (one for each node)
Ours	1	m	m × n (one for each node of each graph)

## Data Availability

Processed data used in this paper, including the cell line gene expression dataset, protein-protein interaction network, drug targets, and disease-associated genes, are available via the project website at https://zitniklab.hms.harvard.edu/projects/PDGrapher or directly at https://figshare.com/articles/dataset/Combinatorial_predictionoftherapeutictargetsusingacausally-inspiredneuralnetwork/24798855. The raw protein-protein interaction network data was obtained from https://downloads.thebiogrid.org/File/BioGRID/Release-Archive/BIOGRID-3.5.186/BIOGRID-MV-Physical-3.5.186.tab3.zip,https://www.science.org/doi/suppl/10.1126/science.1257601/supplile/datasets_s1-s4.zip, and http://www.interactome-atlas.org/data/HuRI.tsv. Raw gene expression datasets were obtained from https://clue.io/releases/data-dashboard. Disease-associated genes were obtained from COSMIC at https://cancer.sanger.ac.uk/cell_lines/archive-download#:~:text=Complete%20mutation%20data and https://cancer.sanger.ac.uk/cosmic/curation. Drug targets were extracted from DrugBank at https://go.drugbank.com/releases/5-1-9, and a list of cancer drugs was obtained from NCI at https://www.cancer.gov/about-cancer/treatment/types/targeted-therapies/approved-drug-list.
