# Supplementary material for: Combinatorial prediction of therapeutic perturbations using causally-inspired neural networks
Source: bioRxiv. 2025 Jan 28:2024.01.03.573985. Preprint. [Version 4] doi: 10.1101/2024.01.03.573985 (PMC10802439; doi:10.1101/2024.01.03.573985)
Supplement: Supplementary file 1 [file media-1.pdf]

Supplementary Information for

Combinatorial prediction of therapeutic perturbations using causally-inspired neural networks

Guadalupe Gonzalez[1,2,3,*], Xiang Lin[7,*], Isuru Herath[4,5], Kirill Veselkov[1],
Michael Bronstein[6] and Marinka Zitnik[7,8,9,10]

[1]Imperial College London, London, UK
[2]Prescient Design, Genentech, South San Francisco, CA, USA
[3]F. Hoffmann-La Roche Ltd, Basel, Switzerland
[4]Merck & Co., South San Francisco, CA, USA
[5]Cornell University, Ithaca, NY, USA
[6]University of Oxford, Oxford, UK
[7]Harvard Medical School, Boston, MA, USA
[8]Kempner Institute for the Study of Natural and Artificial Intelligence, Harvard University, Cambridge, MA, USA
[9]Broad Institute of MIT and Harvard, Cambridge, MA, USA
[10]Harvard Data Science Initiative, Cambridge, MA, USA

[*]Co-first authors
[‡]Corresponding author. Email: marinka@hms.harvard.edu

This PDF file includes:

- Supplementary Notes 1 to 2
- Supplementary Tables 1 to 12
- Supplementary Figures 1 to 17

Supplementary Notes

Note 1. PDGrapher illuminates mode of action of chemical perturbagens: a case study for Vorinostat and Sorafenib predicted by PDGrapher for lung cancer

To demonstrate PDGrapher's ability to illuminate the mechanism of action of therapeutic perturbagens, we analyze PDGrapher's predictions for Vorinostat and Sorafenib in Chemical-PPI-Lung in the random splitting setting. We visualize ground truth and predicted combinations of therapeutic targets together with their one-hop neighbors in the protein interaction network using Gephi [1] and the ForceAtlas graphical layout (Figure S5). The network structure reveals distinct community clusters, identified by Gephi's modularity algorithm, with clear visual delineation based on the predicted and known targets. This analysis demonstrates the model's ability to correctly identify several key therapeutic targets while also proposing novel candidates, potentially elucidating alternative pathways for drug action.

Note 2A. Scoring system of different evidence sources

Open Targets utilizes 23 sources of evidence across six categories: Drug (1), Genetic Association (9), Pathology and Systems Biology (7), Text Mining (1), Somatic Mutation (3), Gene Expression (1), and Animal Model (1). However, the Animal Model database was excluded due to a lack of relevant evidence. Most evidence sources employ different scoring systems based on distinct criteria and statistical methods. We have summarized the database names, evidence types, weights, scoring systems, and background information for each evidence source in Table S8.

Note 2B. Association score for one or multiple types of evidence

Open Targets calculates a association score for each target (such as a gene or protein) by integrating the scores of evidence in multiple types (see the seven evidence types mentioned above). Each type of evidence contributes to the global score based on a weighted system. For example, evidence from drug and genetic association databases is assigned higher weights (1) due to its experimental nature, while evidence from text mining and computational models receives lower weights (0.2) as it is considered indirect evidence. Here we listed the weights for each database: Europe PubMed Central (0.2), Expression Atlas (0.2), IMPC (0.2); PROGENy (0.5), SLAPenrich (0.5), Cancer Biomarkers (0.5), SysBio/gene signature (0.5) and all other database (1). To calculate the Association score ($\hat{S}_i$ for target i, the scores from all evidence are first sorted as

$$R_{ij} = Rank(S_{ij}) \tag{1}$$

Where E_{ij} and R_{ij} indicate the score and rank of the j th evidence for target i, respectively. Function $Rank()$ returns the rank of each data point in the input vector (greater value gets lower rank). Then a harmonic sum is calculated as

$$\bar{S}_i = \sum_j W_j / R_{ij} \tag{2}$$

Where $\bar{S}_i$ is the raw global score of target i and W_j is the predefined weight for evidence j. The final global score ($\hat{S}_i$) is calculated by scaling $\bar{S}$ into the range of 0-1 by dividing $\bar{S}_i$ by the maximum theoretical harmonic sum. By using this approach, stronger and more reliable evidence types have a greater impact on the final score: 0 indicates no evidence and 1 indicates very strong evidence. We also use this approach to calculate a score for each type of evidence (Drug, genetic association, pathology and systemic biology, text mining, somatic mutation and gene expression) which is not provided by OpenTarget.

Supplementary Tables

Table S1: Number of seen and unseen perturbagens per cell line and across dataset splits, considering chemical perturbations data.

Cell line	Split	Total	Seen	Unseen
Chemical-PPI/GRN-Lung-A549	1	858	841	17
	2	850	828	22
	3	834	821	13
	4	853	837	16
	5	839	825	14
Chemical-PPI/GRN-Breast-MCF7	1	1043	1039	4
	2	1041	1037	4
	3	1038	1032	6
	4	1048	1044	4
	5	1048	1046	2
Chemical-PPI/GRN-Prostate-PC3	1	1088	1086	2
	2	1075	1073	2
	3	1090	1086	4
	4	1088	1085	3
	5	1075	1071	4
Chemical-PPI/GRN-Prostate-VCAP	1	574	568	6
	2	575	565	10
	3	585	579	6
	4	567	555	12
	5	557	547	10
Chemical-PPI/GRN-Breast-MDAMB231	1	458	454	4
	2	469	458	11
	3	464	459	5
	4	459	459	0
	5	453	448	5
Chemical-PPI/GRN-Breast-BT20	1	37	37	0
	2	38	38	0
	3	39	37	2
	4	37	37	0
	5	38	38	0
Chemical-PPI/GRN-Colon-HT29	1	884	879	5
	2	916	901	15
	3	877	860	17
	4	887	883	4
	5	877	867	10
Chemical-PPI/GRN-Skin-A375	1	924	910	14
	2	933	917	16
	3	911	901	10
	4	948	927	21
	5	939	919	20
Chemical-PPI/GRN-Cervix-HELA	1	659	658	1
	2	657	657	0
	3	669	668	1
	4	649	649	0
	5	664	664	0

Table S2: Number of unseen perturbagens in each dataset split across cell lines and considering chemical perturbation data.

Cell line	Split	A375	A549	BT20	HA1E	HELA	HT29	MCF7	MDAMB231	PC3	VCAP
Chemical-PPI/GRN-Skin-A375	1	14	44	0	32	3	26	118	3	113	22
	2	16	45	0	33	4	26	118	4	114	21
	3	10	41	0	28	2	22	114	2	109	20
	4	21	51	0	39	6	32	124	6	119	27
	5	20	48	0	36	5	29	121	5	116	24
Chemical-PPI/GRN-Lung-A549	1	95	17	0	76	37	64	146	1	173	36
	2	100	22	0	81	36	69	151	0	178	41
	3	94	13	0	74	35	63	145	0	172	36
	4	93	16	0	74	38	62	144	0	171	34
	5	92	14	0	73	34	61	143	0	170	35
Chemical-PPI/GRN-Breast-BT20	1	1055	1003	0	1030	653	1015	1116	488	1144	712
	2	1054	1002	0	1029	652	1014	1115	487	1143	711
	3	1056	1004	2	1031	654	1016	1117	489	1145	713
	4	1055	1003	0	1030	653	1015	1116	488	1144	712
	5	1054	1002	0	1029	652	1014	1115	487	1143	711
Chemical-PPI/GRN-Cervix-HELA	1	413	392	8	386	1	385	486	10	500	357
	2	412	391	8	385	0	384	485	9	499	357
	3	413	392	8	386	1	385	486	10	500	358
	4	413	392	8	386	0	385	486	10	500	358
	5	412	391	8	385	0	384	485	9	499	357
Chemical-PPI/GRN-Colon-HT29	1	53	41	0	32	14	5	117	0	139	9
	2	59	46	0	38	15	15	123	1	145	15
	3	64	52	0	43	16	17	128	0	150	21
	4	52	40	0	30	14	4	116	0	138	9
	5	58	46	0	37	14	10	122	0	144	15
Chemical-PPI/GRN-Breast-MCF7	1	36	14	0	15	14	8	4	0	40	2
	2	38	16	0	17	14	10	4	0	40	4
	3	39	17	0	18	15	11	6	0	42	4
	4	38	16	0	17	14	10	4	0	40	4
	5	37	14	0	16	15	8	2	0	40	2
Chemical-PPI/GRN-Breast-MDAMB231	1	575	521	0	548	172	533	634	4	662	459
	2	583	529	0	556	180	541	642	11	670	463
	3	574	520	0	547	171	532	633	5	661	456
	4	571	517	0	544	168	529	630	0	658	456
	5	577	523	0	550	174	535	636	5	664	460
Chemical-PPI/GRN-Prostate-PC3	1	3	13	0	3	0	2	11	0	2	2
	2	3	13	0	3	1	2	11	1	2	1
	3	3	13	0	3	1	2	14	1	4	1
	4	3	13	0	3	1	2	14	1	3	1
	5	3	13	0	3	1	2	13	1	4	1
Chemical-PPI/GRN-Prostate-VCAP	1	363	327	13	341	305	322	423	245	451	6
	2	369	333	13	347	306	328	429	246	457	10
	3	365	329	12	343	305	324	425	244	453	6
	4	370	334	15	348	307	329	430	247	458	12
	5	371	335	13	349	307	330	431	246	459	10

Table S3: Number of seen and unseen perturbagens per cell line per dataset split with genetic perturbation data.

Cell line	Split	Total	Seen	Unseen
Genetic-PPI-Lung-A549	1	2697	2666	31
	2	2672	2648	24
	3	2681	2656	25
	4	2654	2626	28
	5	2704	2676	28
Genetic-PPI-Breast-MCF7	1	2230	2216	14
	2	2266	2252	14
	3	2255	2247	8
	4	2253	2246	7
	5	2261	2250	11
Genetic-PPI-Prostate-PC3	1	2588	2559	29
	2	2597	2570	27
	3	2635	2609	26
	4	2603	2579	24
	5	2605	2584	21
Genetic-PPI-Skin-A375	1	2552	2507	45
	2	2524	2489	35
	3	2518	2472	46
	4	2565	2504	61
	5	2538	2497	41
Genetic-PPI-Colon-HT29	1	2539	2495	44
	2	2524	2477	47
	3	2544	2506	38
	4	2564	2529	35
	5	2563	2523	40
Genetic-PPI-Ovary-ES2	1	2579	2522	57
	2	2557	2505	52
	3	2591	2526	65
	4	2617	2553	64
	5	2573	2520	53
Genetic-PPI-Head-BICR6	1	2597	2568	29
	2	2601	2573	28
	3	2592	2565	27
	4	2602	2564	38
	5	2573	2549	24
Genetic-PPI-Pancreas-YAPC	1	2502	2458	44
	2	2524	2480	44
	3	2504	2461	43
	4	2491	2453	38
	5	2539	2504	35
Genetic-PPI-Stomach-AGS	1	2574	2544	30
	2	2637	2606	31
	3	2615	2586	29
	4	2573	2542	31
	5	2590	2565	25
Genetic-PPI-Brain-U251MG	1	2784	2772	12
	2	2782	2774	8
	3	2834	2827	7
	4	2810	2796	14
	5	2792	2778	14

Table S4: Number of unseen perturbagens in each dataset split across cell lines and considering genetic perturbation data.

Cell Line	Split	A375	A549	AGS	BICR6	ES2	HT29	MCF7	PC3	U251MG	YAPC
Genetic-PPI-Skin-A375	1	45	59	59	59	64	58	53	59	59	59
	2	35	54	54	54	59	54	53	54	54	54
	3	46	66	66	65	71	66	59	66	66	66
	4	61	80	81	81	85	81	76	81	81	81
	5	41	62	62	62	66	62	56	62	62	62
Genetic-PPI-Lung-A549	1	40	31	35	35	43	35	31	35	35	35
	2	33	24	29	29	37	29	23	29	29	29
	3	35	25	30	30	40	30	26	30	30	30
	4	39	28	34	34	43	34	31	34	34	34
	5	42	28	37	37	45	37	33	36	37	37
Genetic-PPI-Stomach-AGS	1	37	32	30	32	40	32	21	32	32	32
	2	38	32	31	33	42	33	21	33	33	33
	3	36	31	29	31	41	31	25	31	31	31
	4	38	33	31	33	41	33	26	33	33	33
	5	30	26	25	25	34	26	20	26	26	26
Genetic-PPI-Head-BICR6	1	41	36	36	29	43	36	27	36	36	36
	2	39	34	34	28	43	33	31	34	34	34
	3	36	31	31	27	40	31	28	31	31	31
	4	48	44	44	38	52	44	39	44	44	44
	5	39	35	35	24	43	35	30	34	35	34
Genetic-PPI-Ovary-ES2	1	153	151	151	150	57	149	149	150	151	151
	2	152	146	147	147	52	147	143	146	147	147
	3	151	151	151	151	65	151	150	150	151	151
	4	144	141	141	141	64	141	140	140	141	141
	5	148	146	146	146	53	146	144	145	146	146
Genetic-PPI-Colon-HT29	1	60	55	55	55	62	44	46	55	55	55
	2	66	61	61	61	64	47	53	61	61	61
	3	58	53	53	53	62	38	48	53	53	53
	4	52	48	48	48	53	35	43	48	48	48
	5	64	60	60	60	65	40	49	60	60	60
Genetic-PPI-Breast-MCF7	1	642	637	637	637	647	637	14	637	637	637
	2	641	636	636	636	646	636	14	636	636	636
	3	634	630	630	630	640	630	8	630	630	630
	4	634	629	629	629	639	629	7	629	629	629
	5	638	633	633	633	643	633	11	633	633	633
Genetic-PPI-Prostate-PC3	1	37	32	32	32	37	32	30	29	32	32
	2	36	30	31	31	37	31	26	27	31	31
	3	37	32	32	32	37	32	30	26	32	32
	4	32	27	27	27	35	26	22	24	27	27
	5	31	26	26	26	34	26	25	21	26	26
Genetic-PPI-Brain-U251MG	1	17	12	12	12	22	12	11	12	12	12
	2	13	8	8	8	18	8	8	8	8	7
	3	12	7	7	7	17	7	7	7	7	7
	4	19	14	14	14	24	14	13	14	14	14
	5	19	14	14	14	24	14	13	14	14	14
Genetic-PPI-Pancreas-YAPC	1	56	51	51	51	58	51	45	51	51	44
	2	53	48	48	48	56	48	38	48	48	44
	3	56	51	51	51	58	51	44	51	51	43
	4	50	45	45	45	54	45	37	45	45	38
	5	52	47	47	47	54	47	38	47	47	35

Table S5: Table containing number of edges remaining, number of edges removed, fraction of edges removed (fraction of the total number of edges in the PPI), and number of connected components ordered by increasing fraction of bridge edges removed in synthetic experiments (Figure S13).

Fraction	Remaining edges	Removed edges	Removed edges (fraction)	Connected components
0.1	303,500	178	0.0005	90
0.2	303,322	356	0.0011	179
0.3	303,144	534	0.0017	268
0.4	302,964	714	0.0023	358
0.5	302,786	892	0.0029	447
0.6	302,608	1,070	0.0035	536
0.7	302,428	1,250	0.0041	626
0.8	302,250	1,428	0.0047	715
0.9	302,072	1,606	0.0052	804
1.0	301,892	1,786	0.0058	894

Table S6: PDGrapher predicts targets that are closer in the network to ground-truth targets than what would be expected by change in Chemical PPI datasets in splits with new samples. Table with median distance between ground-truth targets and targets predicted by PDGrapher and a Random baseline, together with U-statistic and p-value of one-sided Mann-Whitney U test, and effect size (rank-biserial correlation) with 95% confidence interval.

**Dataset**	**PDGrapher**	**Random**	**Effect size ± CI**	**U**	**p-value**
Colon-HT29	3.0	3.0	$0.1645 \pm [0.1626, 0.1664]$	1.3032e11	< 0.001
Prostate-PC3	3.0	3.0	$0.1142 \pm [0.1127, 0.1156]$	4.1938e11	< 0.001
Cervix-HELA	3.0	3.0	$0.2113 \pm [0.2094, 0.2130]$	1.5735e11	< 0.001
Breast-MCF7	3.0	3.0	$0.2160 \pm [0.2146, 0.2174]$	3.9197e11	< 0.001
Lung-A549	3.0	3.0	$0.3531 \pm [0.3515, 0.3549]$	1.2900e11	< 0.001
Skin-A375	3.0	3.0	$0.2648 \pm [0.2633, 0.2662]$	2.2462e11	< 0.001
Prostate-VCAP	3.0	3.0	$0.2083 \pm [0.2053, 0.2111]$	2.1493e10	< 0.001
Breast-MDAMB231	3.0	3.0	$0.3038 \pm [0.3012, 0.3062]$	2.6933e10	< 0.001
Breast-BT20	2.0	3.0	$0.5748 \pm [0.5699, 0.5799]$	5.7782e8	< 0.001

Table S7: PDGrapher predicts targets that are closer in the network to ground-truth targets than what would be expected by change in Chemical PPI datasets in splits with new samples and cell lines. Table with median distance between ground-truth targets and targets predicted by PDGrapher and a Random baseline, together with U-statistic and p-value of one-sided Mann-Whitney U test, and effect size (rank-biserial correlation) with 95% confidence interval.

**Dataset**	**PDGrapher**	**Random**	**Effect size $\pm$ CI**	**U**	**p-value**
Breast-BT20	2.0	3.0	0.2296 $\pm[0.2264, 0.2326]$	1.5779e10	$<$ 0.001
Breast-MDAMB231	3.0	3.0	0.2423 $\pm[0.2409, 0.2436]$	4.6308e11	$<$ 0.001
Lung-A549	3.0	3.0	0.2191 $\pm[0.2182, 0.2200]$	2.4676e12	$<$ 0.001
Cervix-HELA	3.0	3.0	0.2490 $\pm[0.2481, 0.2500]$	2.3807e12	$<$ 0.001
Breast-MCF7	3.0	3.0	0.2457 $\pm[0.2451, 0.2464]$	6.0754e12	$<$ 0.001
Skin-A375	3.0	3.0	0.2384 $\pm[0.2377, 0.2392]$	3.6503e12	$<$ 0.001
Colon-HT29	3.0	3.0	0.2571 $\pm[0.2562, 0.2581]$	1.8346e12	$<$ 0.001
Prostate-PC3	3.0	3.0	0.2569 $\pm[0.2562, 0.2576]$	5.6374e12	$<$ 0.001
Prostate-VCAP	3.0	3.0	0.2486 $\pm[0.2472, 0.2501]$	3.1982e11	$<$ 0.001

Table S8: Scoring system of evidence for targets (Due to extended size of this table, we provide it as a separate XLSX file.)

Table S9: P-values for all the perturbagen discovery experiments (Due to extended size of this table, we provide it as a separate XLSX file.)

Table S10: P-values for all the response prediction experiments (Due to extended size of this table, we provide it as a separate XLSX file.)

Table S11: Cell line selection in chemical LINCS dataset. For chemical perturbagens, we first extracted cell lines with `trt_cp` perturbation types and no recorded `failure_mode`, and then filtered out any cell lines with fewer than 1,000 treated samples. Next, we applied the following selection criteria: (1) we selected those that had healthy counterparts available, yielding six cell lines, (2) from the remaining cell lines lacking healthy counterparts, we selected those with more than 15,000 treated samples, (3) of all remaining cell lines, we excluded those which were not found in COSMIC Cell Line Project. This table contains all cell lines that have more or equal than 1,000 treated samples and their corresponding reason for exclusion or inclusion.

**Cell line**	**Number of treated samples**	**Healthy counterpart in data**	**Inclusion label**	**Reason for inclusion/exclusion**
MCF7	35935	MCF10A	Included	Meets selection criteria (1)
PC3	33173	RWPE1	Included	Meets selection criteria (1)
A375	25946	-	Included	Meets selection criteria (2)
A549	23423	NL20	Included	Meets selection criteria (1)
HELA	20894	-	Included	Meets selection criteria (2)
HT29	19724	-	Included	Meets selection criteria (2)
YAPC	12598	-	Excluded	Does not meet selection criteria (2)
MDAMB231	10167	MCF10A	Included	Meets selection criteria (1)
THP1	8642	-	Excluded	Does not meet selection criteria (2)
U2OS	7688	-	Excluded	Does not meet selection criteria (2)
VCAP	7469	RWPE1	Included	Meets selection criteria (1)
NPC	7337	-	Excluded	Does not meet selection criteria (2)
HCC515	7176	-	Excluded	Does not meet selection criteria (2)
JURKAT	6334	-	Excluded	Does not meet selection criteria (2)
HEPG2	6138	-	Excluded	Does not meet selection criteria (2)
XC.L10	3993	-	Excluded	Does not meet selection criteria (2)
HAP1	1791	-	Excluded	Does not meet selection criteria (2)
SKBR3	1714	MCF10A	Excluded	Does not meet selection criteria (3)
BT20	1403	MCF10A	Included	Meets selection criteria (1)
SKL	1351	-	Excluded	Does not meet selection criteria (2)
XC.L100	1218	-	Excluded	Does not meet selection criteria (2)
TMDB	1184	-	Excluded	Does not meet selection criteria (2)
XC.R10	1166	-	Excluded	Does not meet selection criteria (2)
HCT116	1143	-	Excluded	Does not meet selection criteria (2)
HS578T	1049	-	Excluded	Does not meet selection criteria (2)
OCILY19	1047	-	Excluded	Does not meet selection criteria (2)

Table S12: Cell line selection in genetic LINCS dataset. For genetic perturbagens, we first extracted cell lines with `trt_xpr` perturbation types and no recorded `failure_mode`, and then filtered out any cell lines with fewer than 5,000 treated samples. Next, we applied the following selection criteria: (1) we selected those that had healthy counterparts available, yielding three cell lines, (2) from the remaining cell lines lacking healthy counterparts, we selected those with more than 15,000 treated samples. This table contains all cell lines that have more or equal than 5,000 treated samples and their corresponding reason for exclusion or inclusion.

**Cell line**	**Number of treated samples**	**Healthy counterpart in data**	**Inclusion label**	**Reason for inclusion/exclusion**
U251MG	28337	-	Included	Meets selection criteria (2)
A549	26094	NL20	Included	Meets selection criteria (1)
ES2	25540	-	Included	Meets selection criteria (2)
A375	23523	-	Included	Meets selection criteria (2)
AGS	23071	-	Included	Meets selection criteria (2)
PC3	23040	RWPE1	Included	Meets selection criteria (1)
BICR6	22981	-	Included	Meets selection criteria (2)
HT29	22220	-	Included	Meets selection criteria (2)
YAPC	21809	-	Included	Meets selection criteria (2)
MCF7	20328	MCF10A	Included	Meets selection criteria (1)

Supplementary Figures

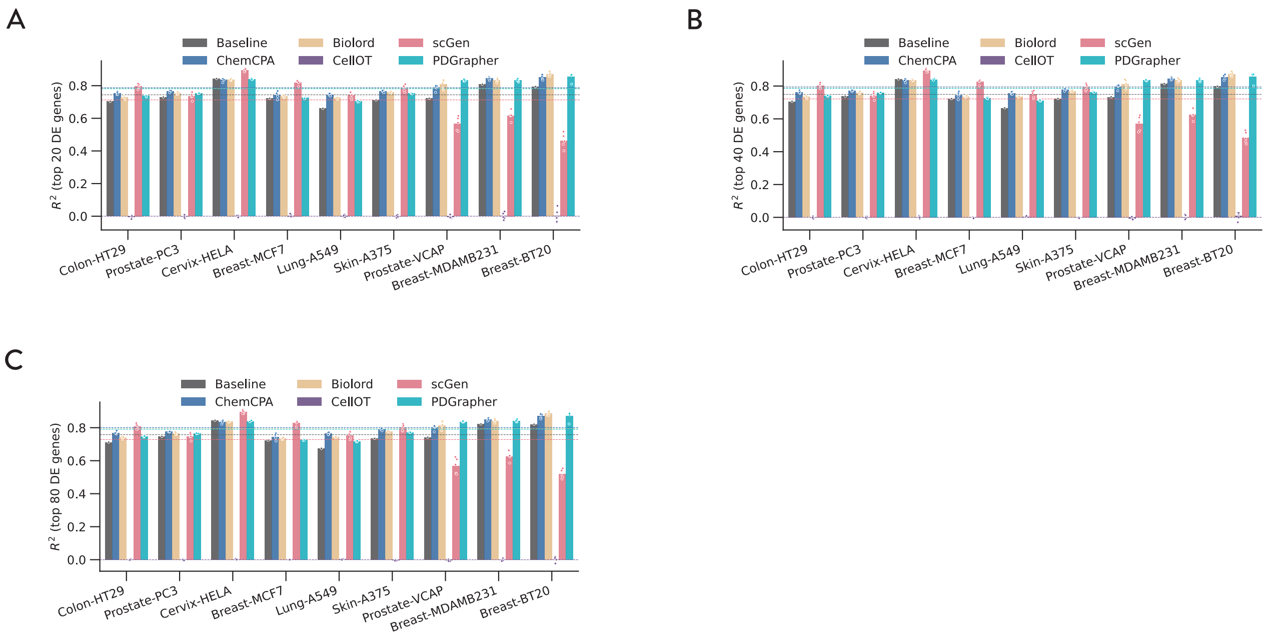

Figure S1: The performance of response prediction within nine cell lines under chemical perturbation. The R^2 values are calculated between the predicted and actual gene expression for the top 20 (**A**), 40 (**B**), and 80 (**C**) differentially expressed genes per cell line. Dotted lines represent the average performance across cell lines, dots indicate individual data points, and bars represent the average R^2 across five data splits

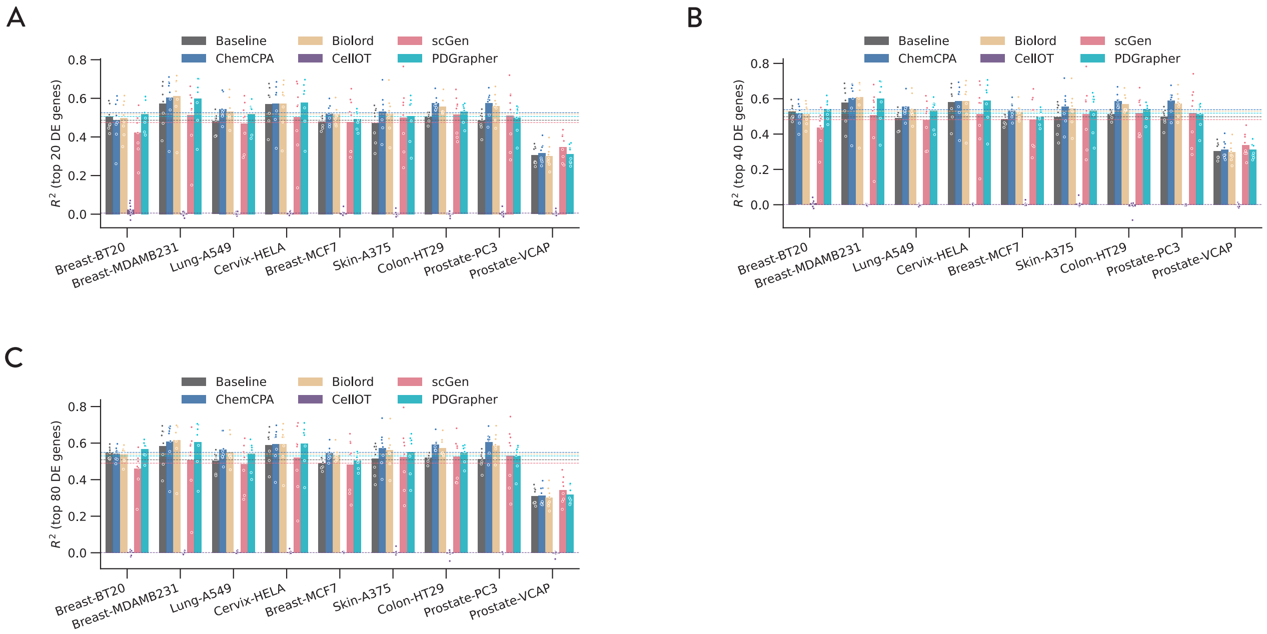

Figure S2: The performance of response prediction across nine cell lines under chemical perturbation. We trained each model on a single cell line and made predictions on the remaining eight cell lines. The R^2 values are calculated between the predicted and actual gene expression for the top 20 (**A**), 40 (**B**), and 80 (**C**) differentially expressed genes per cell line. Dotted lines represent the average performance across cell lines, dots indicate individual data points, and bars represent the average R^2 across eight cell lines

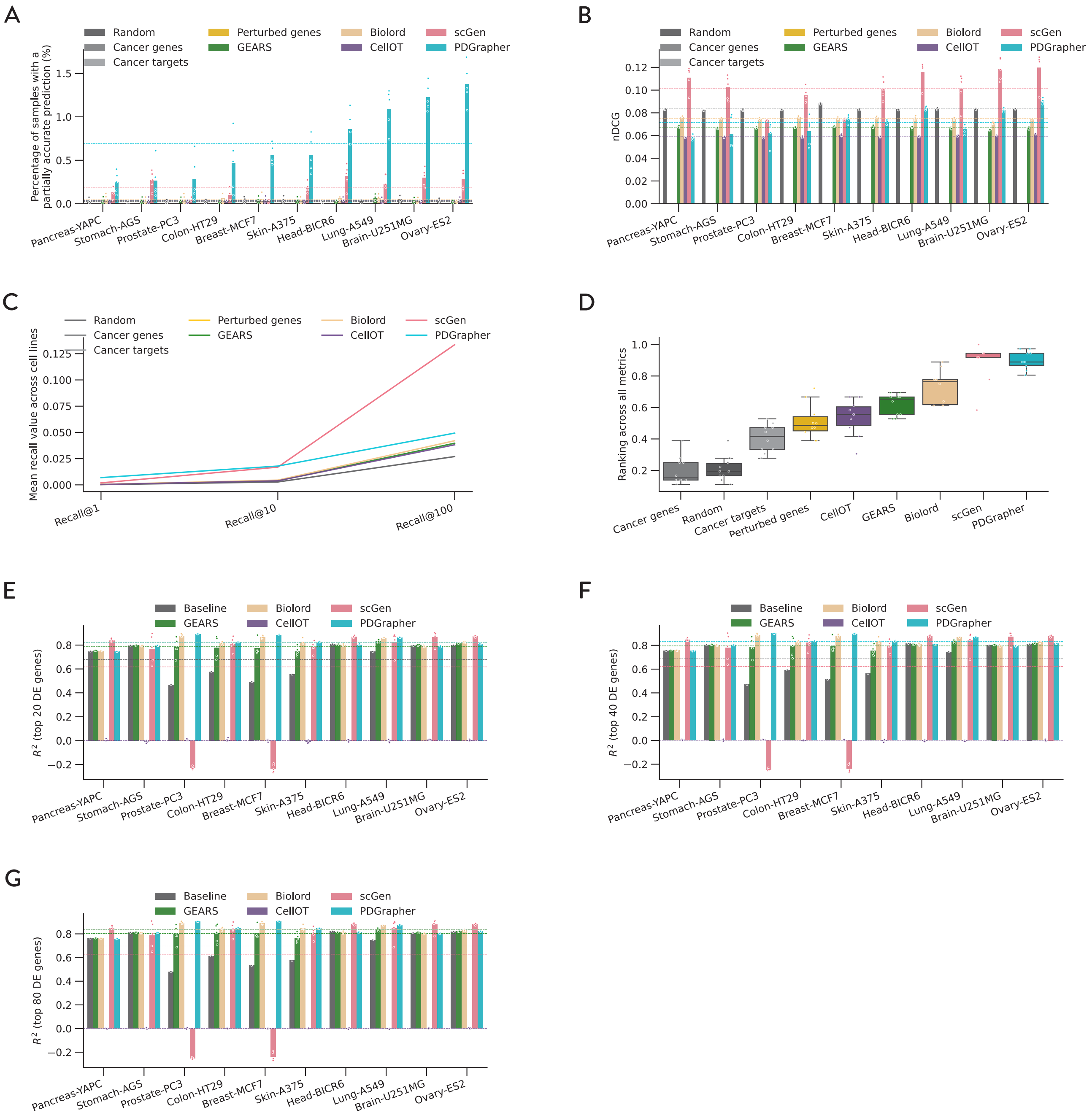

Figure S3: PDGrapher efficiently predicts genetic perturbagens to shift cells from diseased to treated states in a random splitting setting within ten cell lines. **(A)** PDGrapher provides accurate predictions for up to 1.09% (Genetic-PPI-Ovary-ES2: 1.37% vs 0.28%) more samples in the test set compared to the second-best baseline across Genetic-PPI datasets **(B)** scGen takes the leading position in nDCG across genetic Genetic-PPI datasets. **(C)** PDGrapher recovers ground-truth therapeutic targets at comparable rates compared to competing methods for Genetic-PPI datasets. **(D)** PDGrapher has the best overall performance in perturbagen prediction within each cell line evaluated by the averaged rank over multiple cell lines and metrics. **(E-G)** Shown is the R^2 of the response prediction module of PDGrapher compared to competing baselines for the top 20 (E), 40 (F), and 80 (G) differentially expressed (DE) genes.

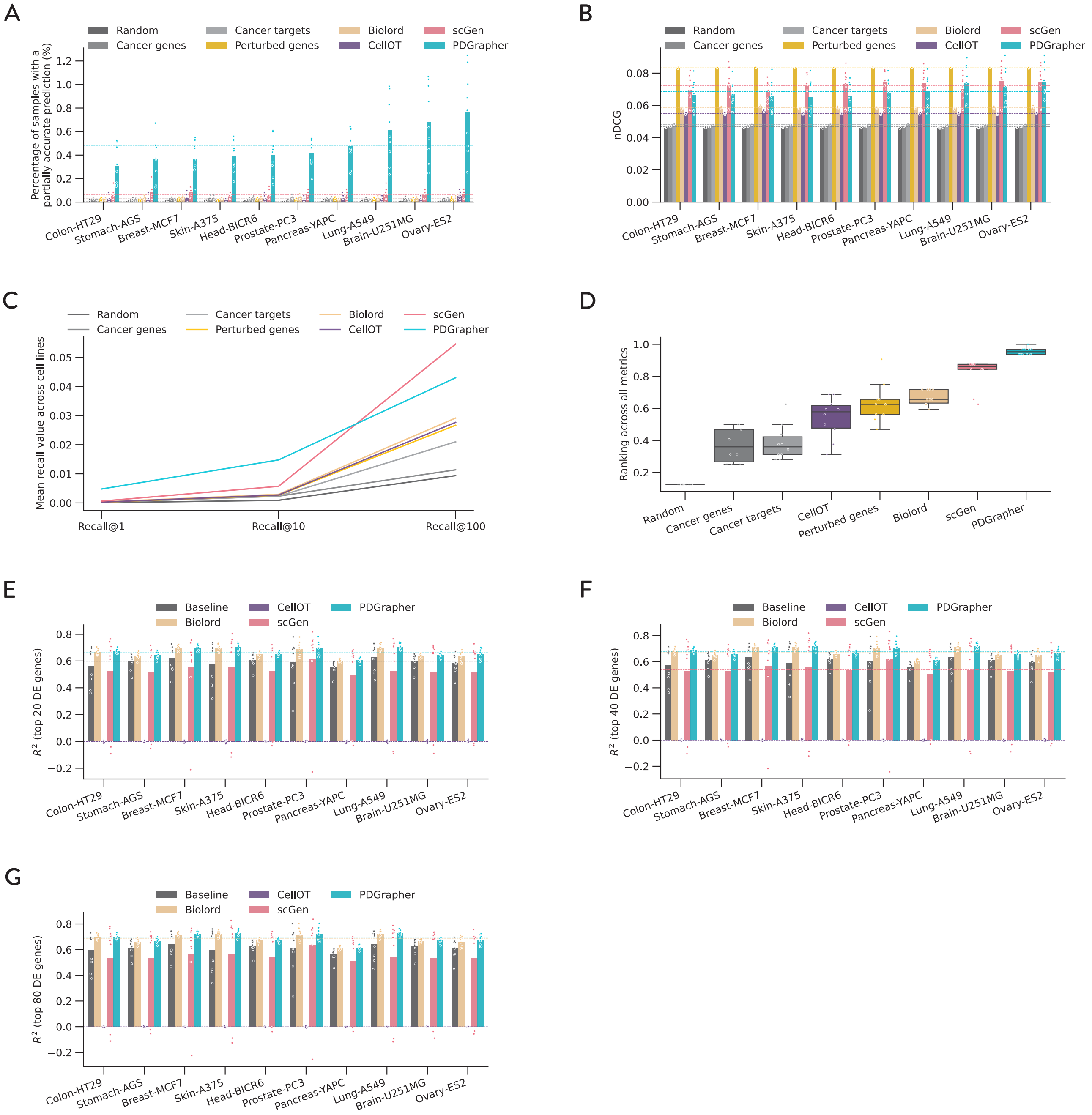

Figure S4: PDGrapher predicts genetic perturbagens to shift cells from diseased to treated states in unseen cell lines. **(A)** PDGrapher provides accurate predictions for up to 0.68% (Genetic-PPI-Ovary-ES2: 0.75% vs 0.07%) more samples in the test set compared to the second-best baseline across Genetic-PPI datasets **(B)** The Perturbed genes baseline takes the leading position in nDCG across genetic Genetic-PPI datasets. **(C)** PDGrapher recovers ground-truth therapeutic targets at comparable rates compared to competing methods for Genetic-PPI datasets. **(D)** PDGrapher has the second-best overall performance in perturbagen prediction in unseen cell lines evaluated by the averaged rank over multiple cell lines and metrics. **(E-G)** Shown is the R^2 of the response prediction module of PDGrapher compared to competing baselines for the top 20 (E), 40 (F), and 80 (G) differentially expressed (DE) genes.

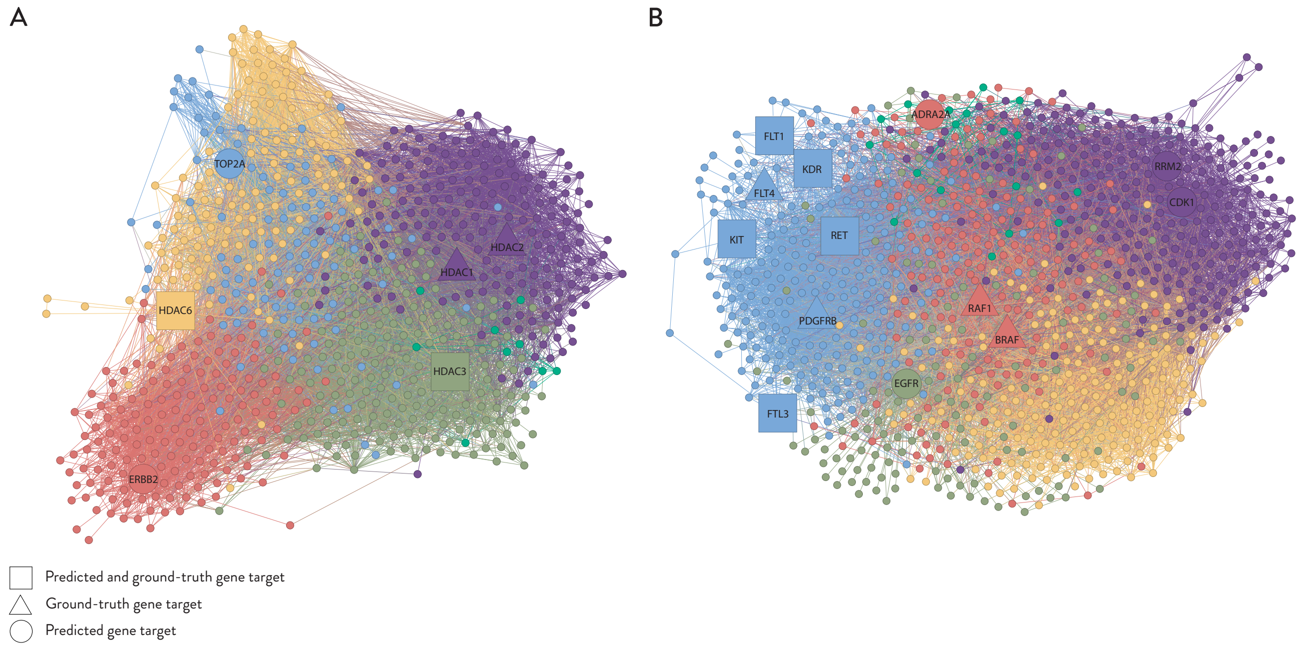

Figure S5: Network visualization of predicted and ground-truth targets can aid in ellucidation of mechanism of action of drugs. (A,B) We visualize ground-truth, and predicted therapeutic targets for Vorinostat (A) and Sorafenib (B) in Chemical-PPI-Lung-A549 using Gephi with ForceAtlas embedding. We highlight in different colors distinct communities identified by Gephi's modularity algorithm.

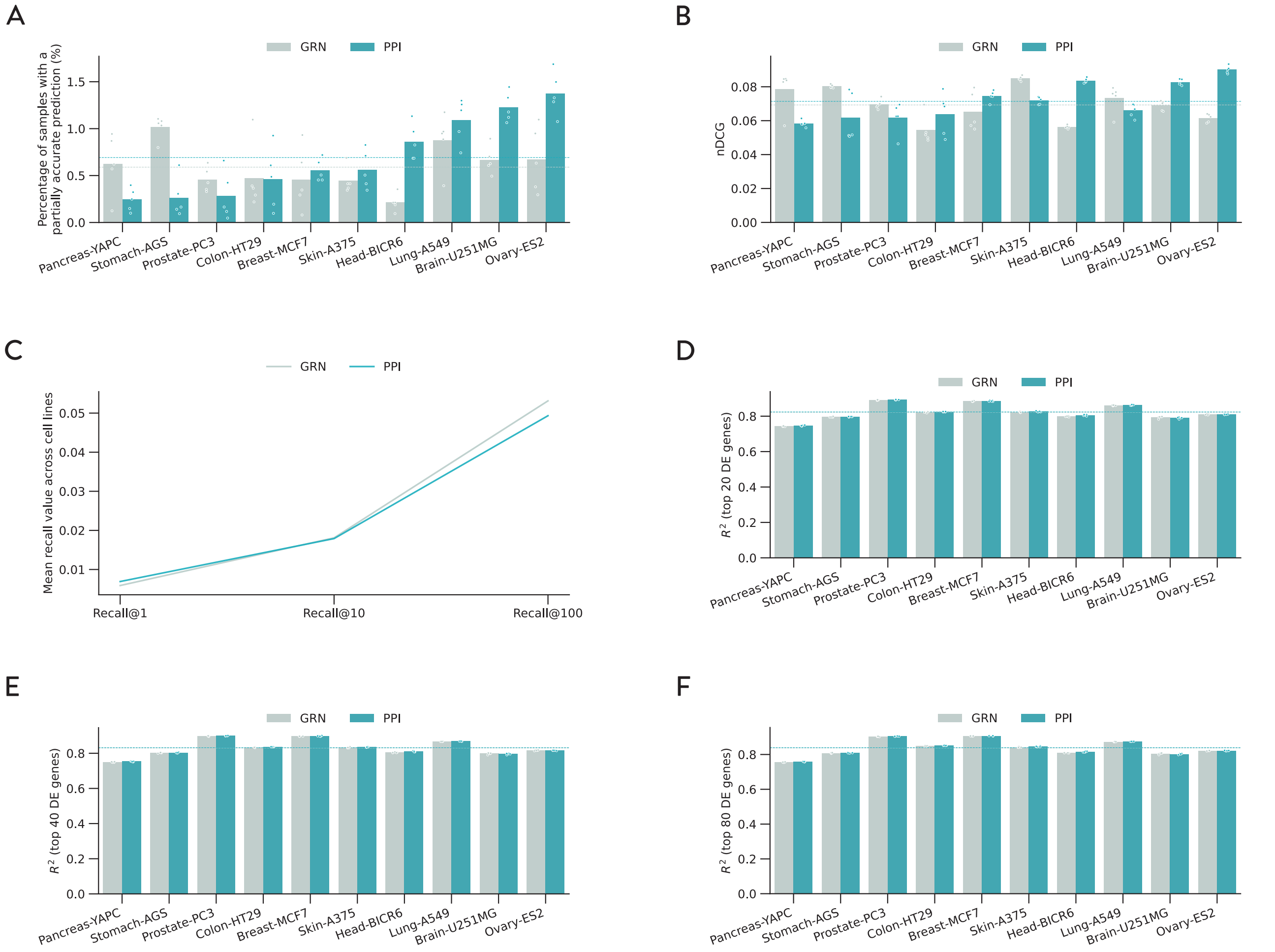

Figure S6: PDGrapher predicts genetic perturbagens to shift cells from diseased to treated states and predicts response gene expression to genetic perturbagens using cell type specific GRNs. **(A)** PDGrapher using cell type specific GRN (PDGrapher-GRN) provides accurate predictions comparable to PDGrapher using cell type agnostic PPI (PDGrapher-PPI) in cell lines with genetic perturbation. **(B)** PDGrapher-GRN ranks shows nDCG comparable to PDGrapher-PPI. **(C)** Both PDGrapher-PPI and PDGrapher-GRN recover ground-truth therapeutic targets at comparable rates compared to competing methods for genetic datasets. **(D-F)** Shown is the R^2 of the response prediction module of PDGrapher-GRN and PDGrapher-PPI for the top 20 (D), 40 (E), and 80 (F) differentially expressed (DE) genes.

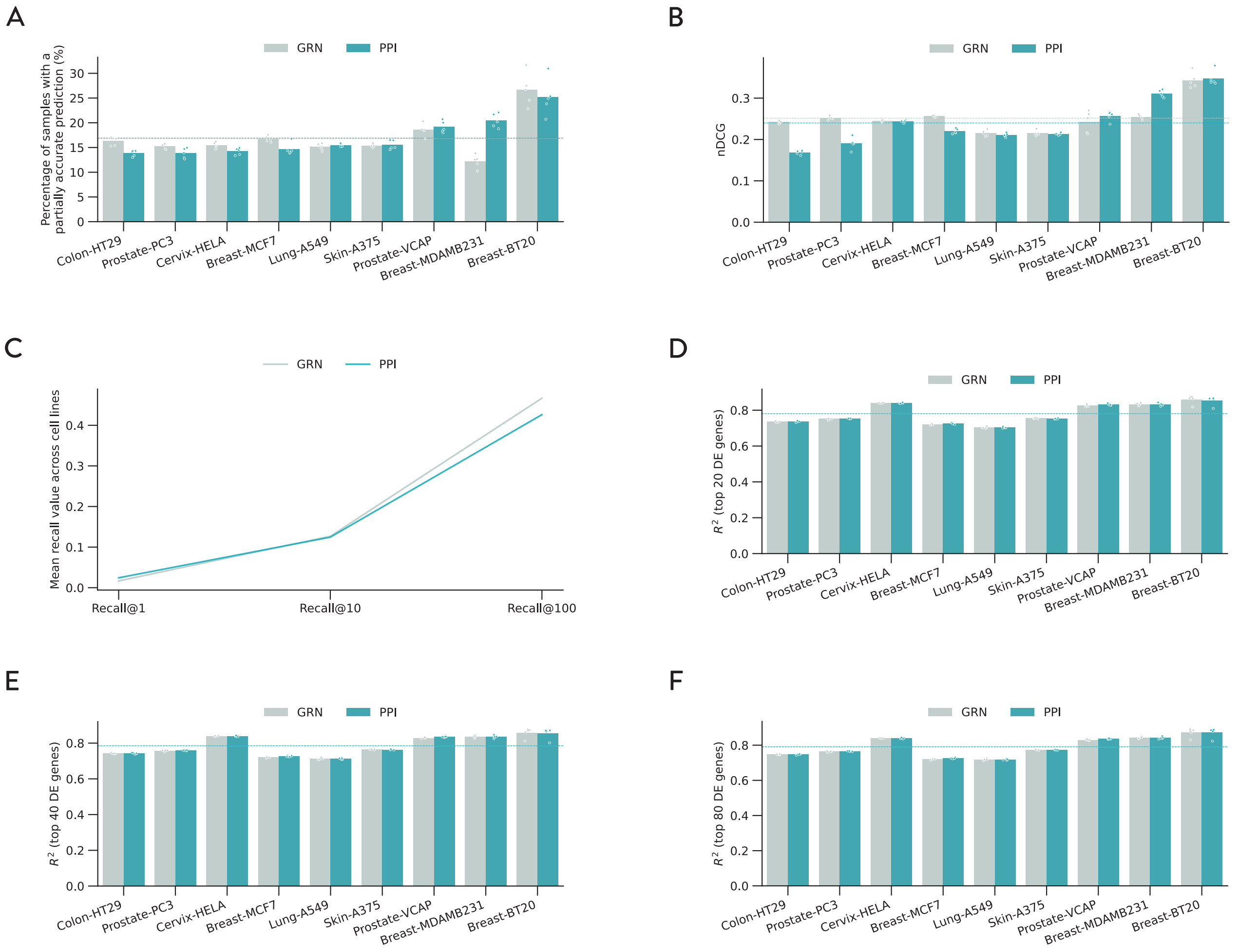

Figure S7: PDGrapher predicts chemical perturbagens to shift cells from diseased to treated states and predicts response gene expression to chemical perturbagens using cell type specific GRNs. **(A)** PDGrapher using cell type specific GRN (PDGrapher-GRN) provides accurate predictions comparable to PDGrapher using cell type agnostic PPI (PDGrapher-PPI) in cell lines with chemical perturbation. **(B)** PDGrapher-GRN shows nDCG comparable to PDGrapher-PPI. **(C)** Both PDGrapher-PPI and PDGrapher-GRN recover ground-truth therapeutic targets at comparable rates for chemical datasets. **(D-F)** Shown is the R^2 of the response prediction module of PDGrapher-GRN and PDGrapher-PPI for the top 20 (D), 40 (E), and 80 (F) differentially expressed (DE) genes.

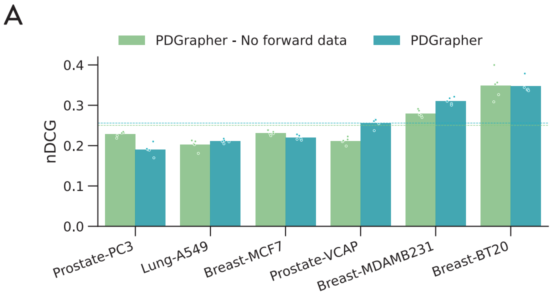

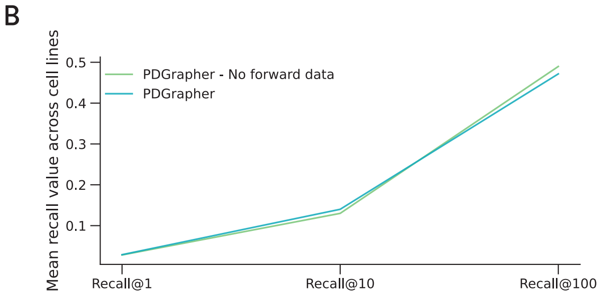

Figure S8: PDGrapher predicts chemical perturbagens to shift cells from diseased to treated states without using disease intervention data. **(A)** PDGrapher (no forward data) shows nDCG comparable to PDGrapher. **(B)** Both PDGrapher and PDGrapher (no forward data) recover ground-truth therapeutic targets at comparable rates for chemical datasets. The results of partial accuracy are shown in Figure 5D.

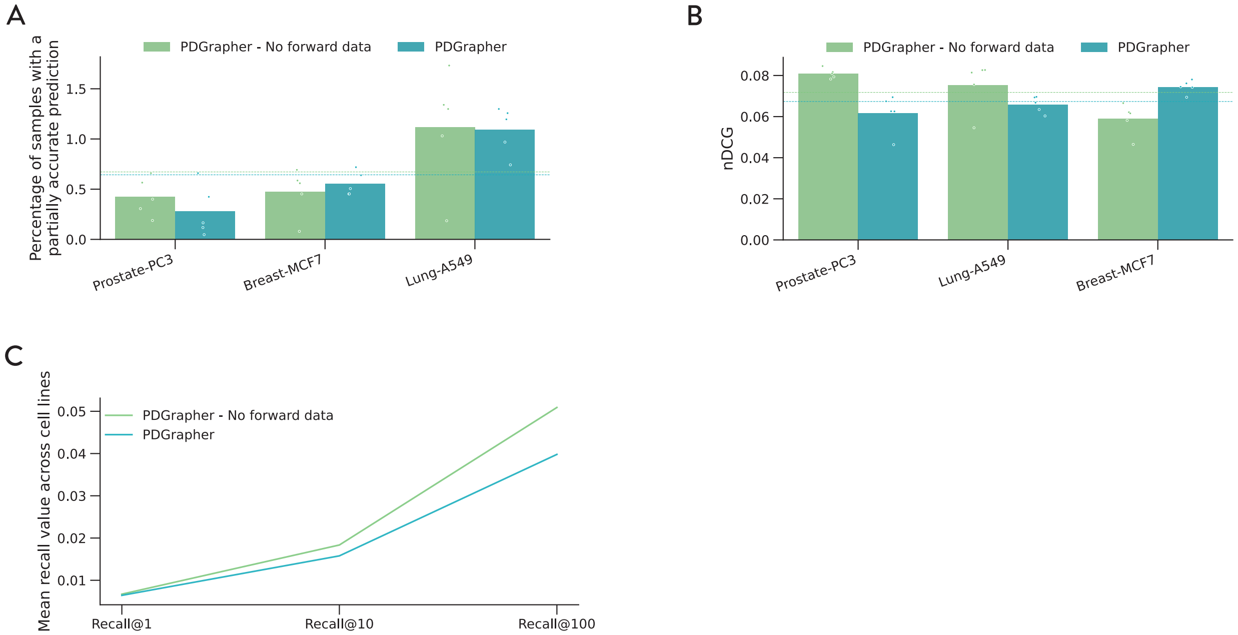

Figure S9: PDGrapher predicts genetic perturbagens to shift cells from diseased to treated states without using disease intervention data. Performance metrics of the second ablation study on PDGrapher's input data: PDGrapher- No forward data using only backward data and PDGrapher using both forward and backward data. The forward and backward data are organized as (healthy, mutation, disease) and (diseased, drug, treated), respectively. **(A) (B)** PDGrapher (no forward data) shows nDCG comparable to PDGrapher. **(C)** Both PDGrapher and PDGrapher (no forward data) recover ground-truth therapeutic targets at comparable rates for genetic datasets.

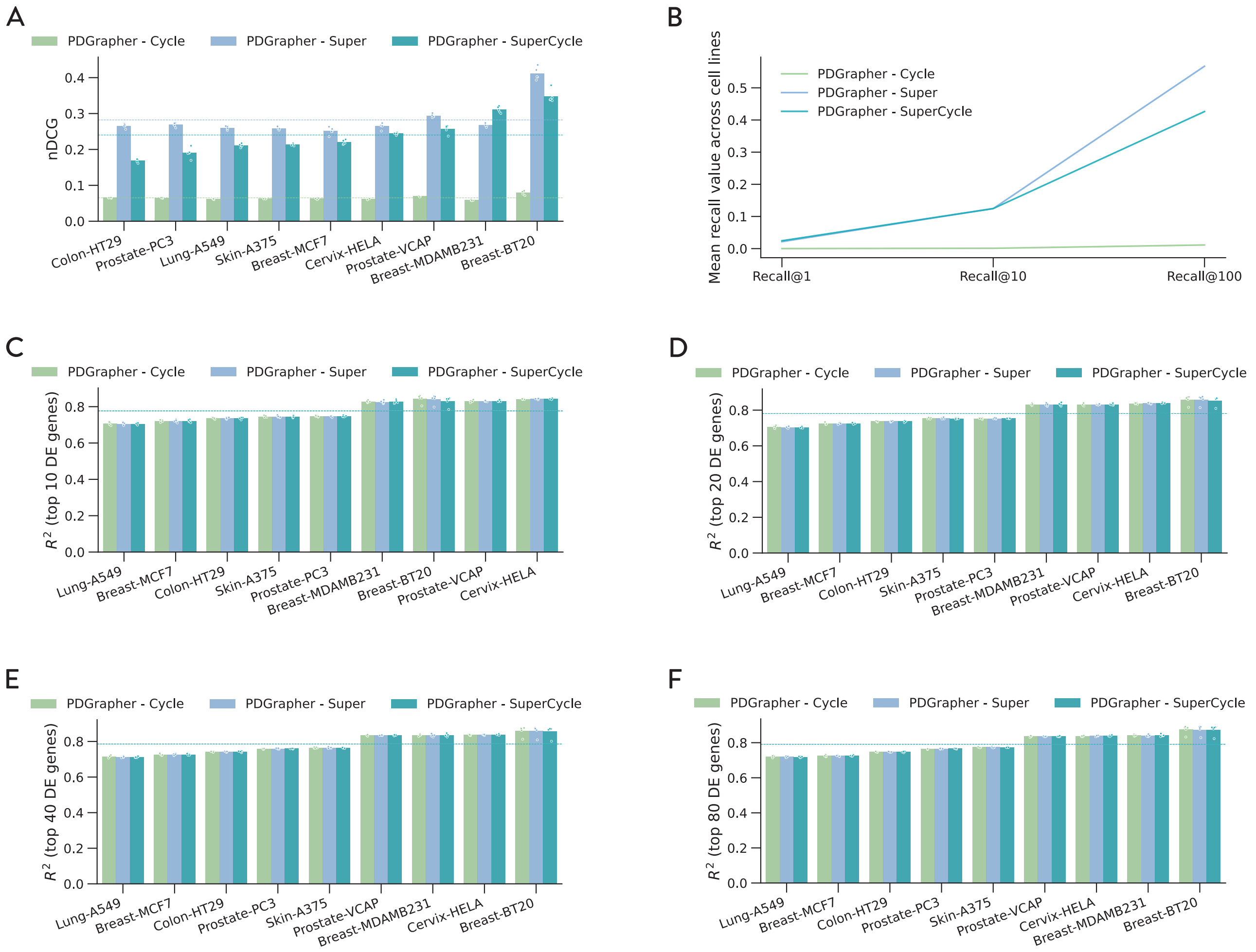

Figure S10: Ablation studies for loss functions of PDGrapher. PDGrapher-Cycle trained using only the cycle loss, PDGrapher-Super trained using only the supervision loss, and PDGrapher-SuperCycle trained using both supervision and cycle loss are compared in this ablation study evaluated by nDCG (**A**), recalls (**B**), and R^2 of response prediction (C-F). The results of partial accuracy are shown in Figure 5C.

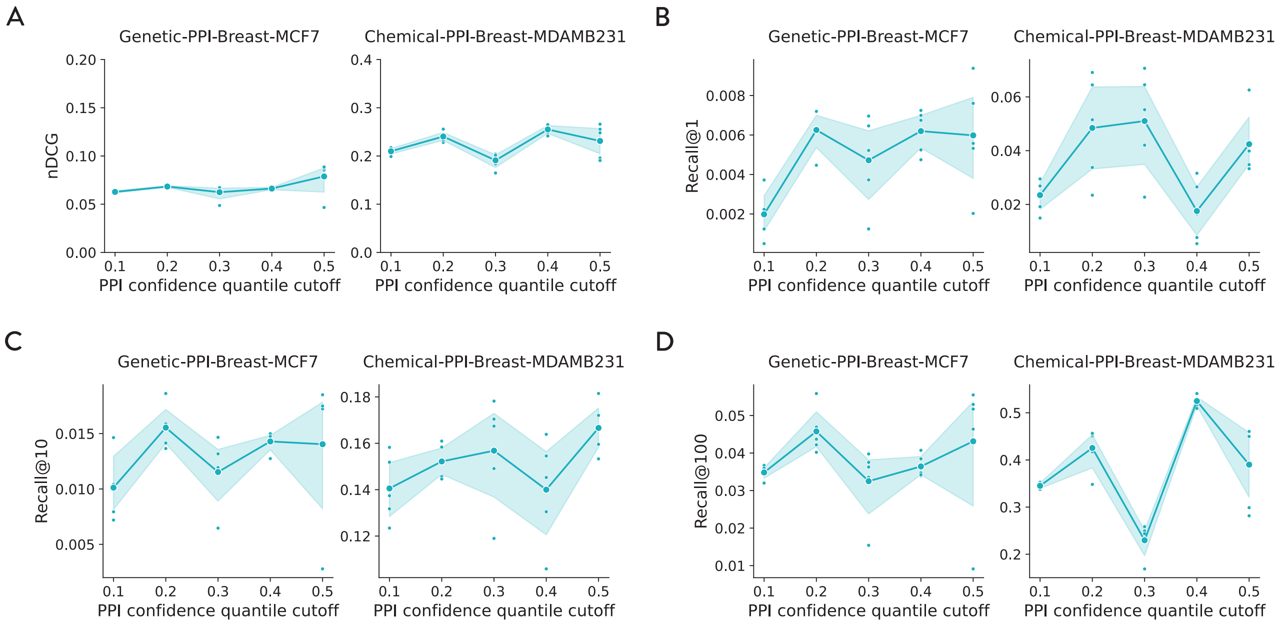

Figure S11: Sensitivity analysis of the PPI used for training PDGrapher. Performance of sensitivity analyses evaluated by nDCG (**A**) and recalls (**B-D**) for datasets Genetic-PPI-Breast-MCF7 (left) and Chemical-PPI-Breast-MDAMB231 (right). The PPI used here is from STRING (string-db.org), which includes a confidence score for each edge. The edges are filtered by the 0.1, 0.2, 0.3, 0.4, and 0.5 quantiles of the confidence scores as cutoffs, resulting in five PPI networks with 625,818, 582,305, 516,683, 443,051, and 296,451 edges, respectively. The results of partial accuracy are shown in Figure 5B.

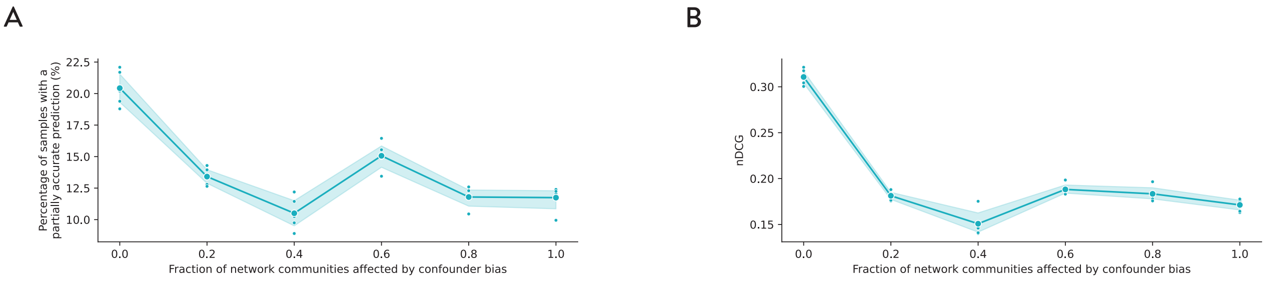

Figure S12: PDGrapher has stable performance on the synthetic datasets with various intensities of confounders added on the gene expression Performance of simulation analyses evaluated by percentage of samples with a partially accurate prediction **(A)** and nDCG **(B)** for the synthetic datasets with with varying levels (0 to 1) of confounding bias introduced into the gene expression data. Gaussian noise, with distinct means and variances, was added progressively to random subsets of genes, simulating latent confounder effects in the treated gene expression data. The intensity of the confounding bias increases as more gene groups (representing network communities) are affected. This approach creates global, controlled variability in the gene expression data, paired with an unperturbed PPI network, allowing for the evaluation of algorithmic performance across different degrees of confounder noise. See Online Methods for more details on data generation.

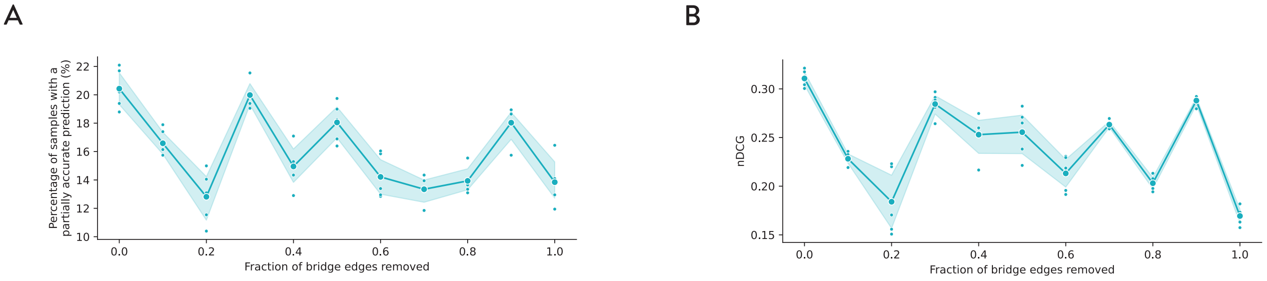

Figure S13: PDGrapher has stable performance on the synthetic datasets with various fractions of bridge edges removed. Performance of simulation analyses evaluated by percentage of samples with a partially accurate prediction **(A)** and nDCG **(B)** for the synthetic datasets with a [0, 0.1, ..., 1] fraction of bridge edges removed in the simulated PPI. Bridge edges are those with high connectivity in the network which, if removed, increase the number of disconnected communities. The number of connected components in the network upon bridge edge removal are [90, 179, 268, 358, 447, 536, 626, 715, 804, 894]. See more information in Table S5.

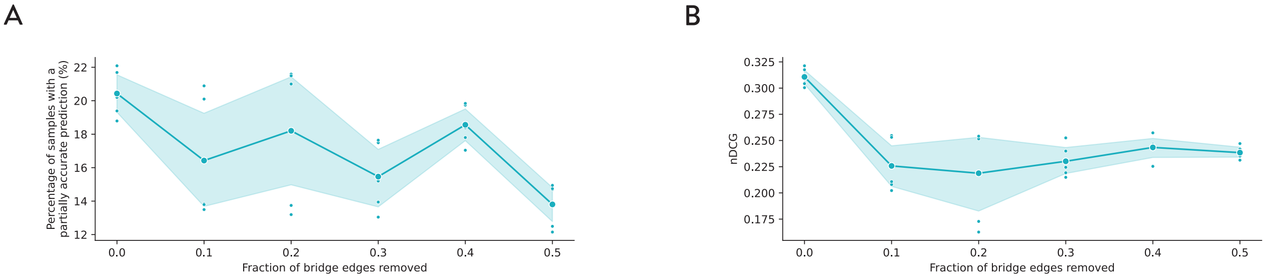

Figure S14: PDGrapher's performance is influenced by network incompleteness. Performance in ablation studies evaluated by percentage of samples with a partially accurate prediction **(A)** and nDCG **(B)** for the synthetic datasets with a [0, 0.1, … 0.6] fraction of random edges removed in the PPI. The number of remaining edges in the network upon random edge removal are [273,319; 242,912; 212,525; 182,177; 151,811; 121,472]. See the Methods section for more details.

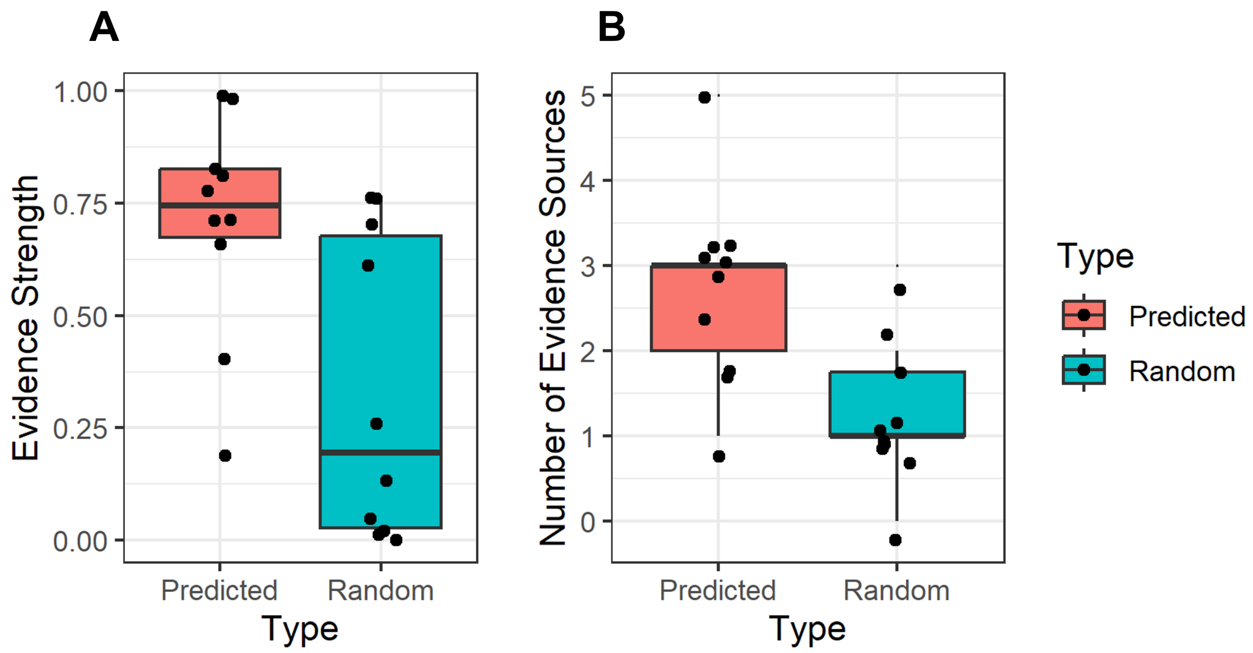

Figure S15: Comparison of predicted targets from PDGrapher and a random model for lung cancer. Boxplots display the evidence strength (A) and the number of evidence sources (B) for the top 10 predicted targets from PDGrapher versus 10 randomly selected genes. The central line inside the box represents the median, while the top and bottom edges correspond to the first (Q1) and third (Q3) quartiles. The whiskers extend to the smallest and largest values within 1.5 times the interquartile range (IQR) from the quartiles.

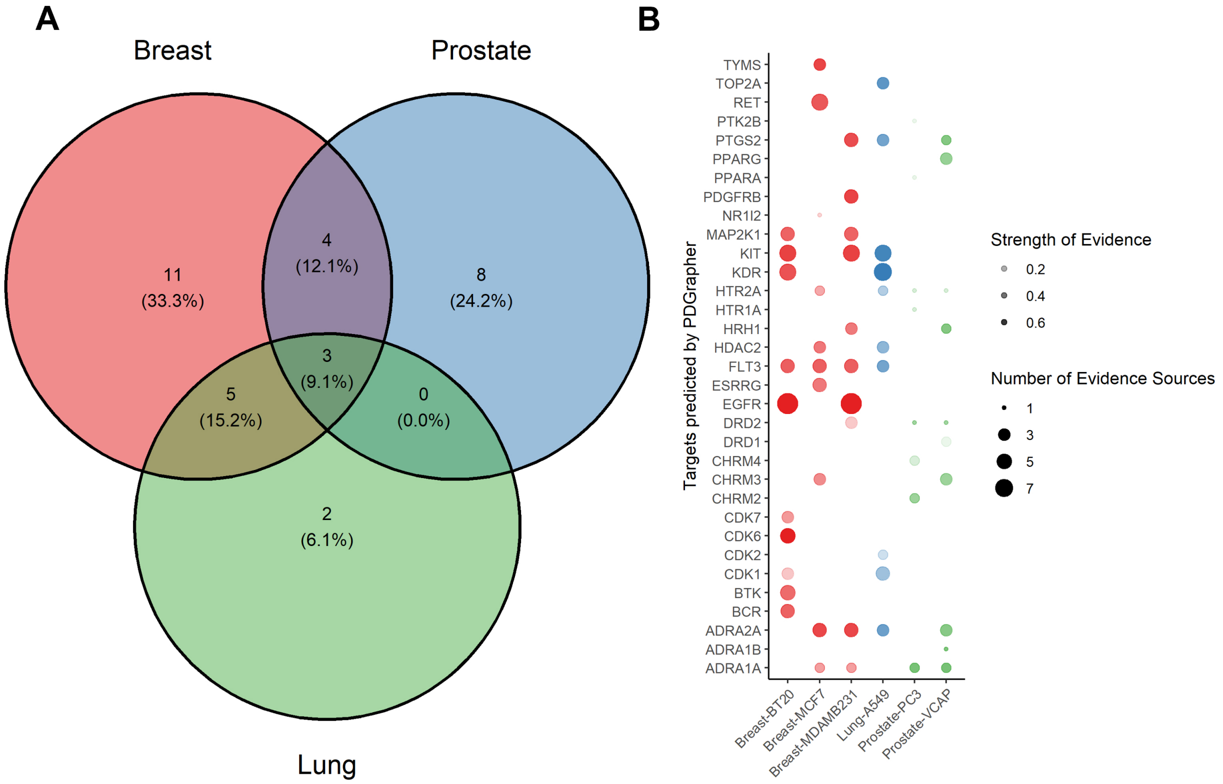

Figure S16: Unique and common targets predicted by PDGrapher among three cancer types. The Venn diagram (A) shows the number and ratio of unique and common predicted targets, while the bubble plot (B) shows the strength of evidence and the number of evidence sources for each cell line. The evidence strengths are the global association scores and overall evidence sources provided by OpenTargets. Details of the scoring system are in Supplementary Note 2.

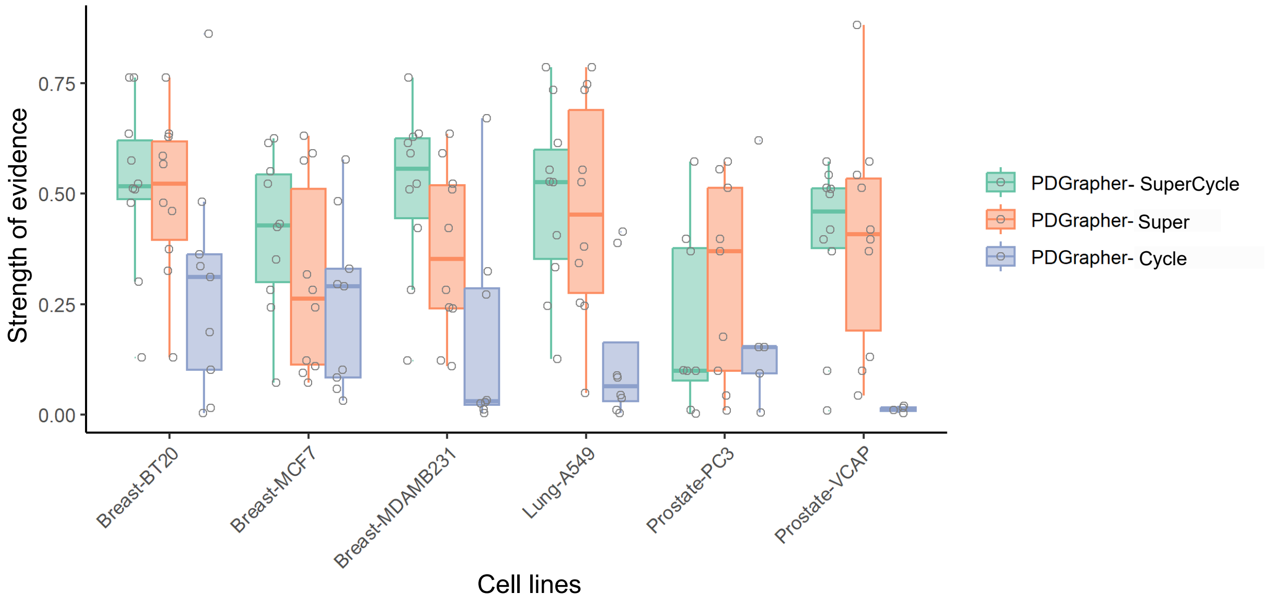

Figure S17: Ablation studies for loss functions of PDGrapher evaluated by the evidence from OpenTargets. The strength of evidence in the cell lines with healthy control data is shown in the box plots. The strength of the evidence is the global association scores, which are based on all evidence sources provided by OpenTargets. See details of the scoring system in Supplementary Note 2. The central line inside the box represents the median, while the top and bottom edges correspond to the first (Q1) and third (Q3) quartiles. The whiskers extend to the smallest and largest values within 1.5 times the interquartile range (IQR) from the quartiles.

Supplementary references

1. Bastian, M., Heymann, S. & Jacomy, M. Gephi: An open source software for exploring and manipulating networks. BT - International AAAI Conference on Weblogs and Social. *International AAAI Conference on Weblogs and Social Media* 361–362 (2009).
