# Supplementary material for: Combinatorial prediction of therapeutic perturbations using causally-inspired neural networks
Source: bioRxiv. 2024 Jan 8:2024.01.03.573985. Preprint. [Version 2] doi: 10.1101/2024.01.03.573985 (PMC10802439; doi:10.1101/2024.01.03.573985)
Supplement: Supplementary file 1 [file media-1.pdf]

Supplementary Information for

Combinatorial prediction of therapeutic perturbations using causally-inspired neural networks

Guadalupe Gonzalez[1,2,3], Isuru Herath[4,5], Kirill Veselkov[1], Michael Bronstein[6] and Marinka Zitnik[7,8,9,10]

[1]Imperial College London, London, UK

[2]Prescient Design, Genentech, South San Francisco, CA, USA

[3]F. Hoffmann-La Roche Ltd, Basel, Switzerland

[4]Merck & Co., South San Francisco, CA, USA

[5]Cornell University, Ithaca, NY, USA

[6]University of Oxford, Oxford, UK

[7]Harvard Medical School, Boston, MA, USA

[8]Kempner Institute for the Study of Natural and Artificial Intelligence, Harvard University, Cambridge, MA, USA

[9]Broad Institute of MIT and Harvard, Cambridge, MA, USA

[10]Harvard Data Science Initiative, Cambridge, MA, USA

‡Corresponding author. Email: marinka@hms.harvard.edu

This PDF file includes:

Supplementary Figures 1 to 4

Supplementary Figures

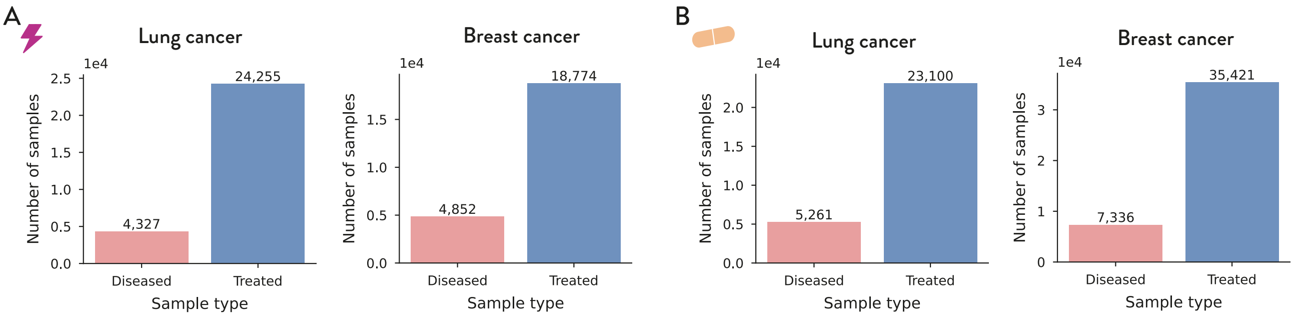

Figure S1: Overview of interventional datasets. (A,B) We train PDGRAPHER across 2 genetic (A) and 2 chemical (B) intervention datasets, where each dataset is comprised of healthy, diseased, and treated gene expression samples for one cell line (lung cancer cell line A549; breast cancer cell line MCF7), and one proxy causal graph (PPI or GRN). This leads to 8 datasets: Genetic-PPI-Lung, Genetic-PPI-Breast, Chemical-PPI-Lung, Chemical-PPI-Breast, Genetic-GRN-Lung, Genetic-GRN-Breast, Chemical-GRN-Lung, and Chemical-GRN-Breast.

A

Model	Relative position of ground-truth genes in the predicted ranking	Proportion of samples with a partially accurate prediction	Training time (mins)
Random	0.50 ± 0.00	0.00 ± 0.00	0
Cancer genes	0.50 ± 0.00	0.00 ± 0.00	0
Cancer targets	0.51 ± 0.00	0.00 ± 0.00	0
scGen	-	0.00 ± 0.00	3,644.2
PDGrapher	**0.65 ± 0.06**	**0.02 ± 0.00**	119.09

B

Model	Relative position of ground-truth genes in the predicted ranking	Proportion of samples with a partially accurate prediction	Training time (mins)
Random	0.50 ± 0.00	0.00 ± 0.00	0
Cancer genes	0.50 ± 0.00	0.00 ± 0.00	0
Cancer targets	0.51 ± 0.01	0.00 ± 0.00	0
scGen	-	0.00 ± 0.00	1,287.89
PDGrapher	**0.67 ± 0.11**	**0.01 ± 0.00**	135.07

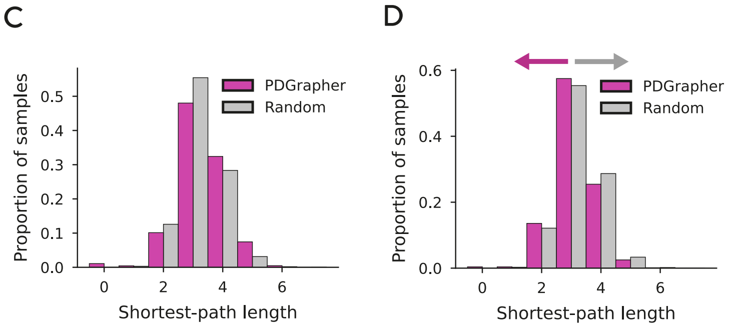

Figure S2: PDGRAPHER efficiently predicts genetic perturbagens to shift cells from diseased to treated states in a random splitting setting. (A,B) We benchmark PDGRAPHER's performance in Genetic-PPI-Lung (A) and Genetic-PPI-Breast (B) in the random-splitting setting. PDGRAPHER achieves the highest performance in the combinatorial prediction of therapeutic targets and is trained up to 30 times faster than the well-established baseline scGen [1] for indirect prediction of perturbagens. **(C,D)** Target genes predicted by PDGRAPHER are closer in the network to ground-truth therapeutic genes compared to what would be expected by chance for Genetic-PPI-Breast (D), which is not observed for Genetic-PPI-Lung (E).

A

Model	Relative position of ground-truth genes in the predicted ranking	Proportion of samples with a partially accurate prediction
Random	0.50 ± 0.00	0.00 ± 0.00
Cancer genes	0.50 ± 0.00	0.00 ± 0.00
Cancer targets	0.50 ± 0.00	0.00 ± 0.00
scGen	-	0.00 ± 0.00
PDGrapher	**0.64 ± 0.09**	**0.01 ± 0.00**

B

Model	Relative position of ground-truth genes in the predicted ranking	Proportion of samples with a partially accurate prediction
Random	0.50 ± 0.00	0.00 ± 0.00
Cancer genes	0.50 ± 0.00	0.00 ± 0.00
Cancer targets	0.50 ± 0.00	0.00 ± 0.00
scGen	-	0.00 ± 0.00
PDGrapher	**0.65 ± 0.07**	**0.01 ± 0.00**

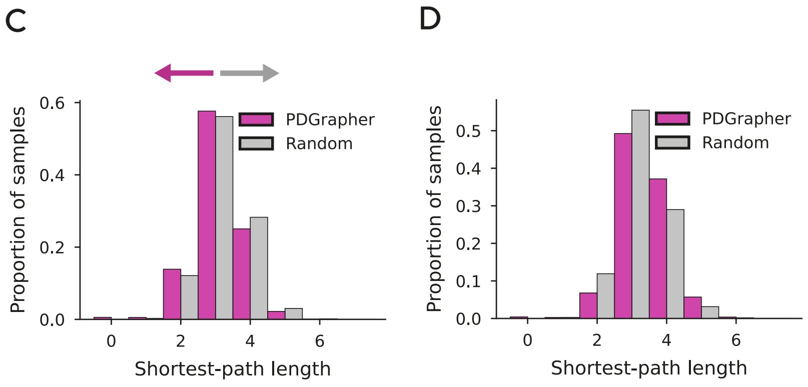

Figure S3: PDGrapher predicts genetic perturbagens to shift cells from diseased to treated states in unseen cell lines. (A,B) We benchmark PDGrapher's performance in Genetic-PPI-Lung (A) and Genetic-PPI-Breast (B) in the leave-cell-out splitting setting. **(C,D)** Therapeutic genes predicted by PDGrapher are closer in the network to ground-truth target genes compared to what would be expected by chance for Genetic-PPI-Lung (C), which is not observed for Genetic-PPI-Breast (D).

A

Split type	Dataset	Relative position of ground-truth genes in the predicted ranking	Proportion of samples with a partially accurate prediction
Random	Genetic-GRN-Lung	0.65 ± 0.05	0.01 ± 0.00
	Genetic-GRN-Breast	0.67 ± 0.07	0.01 ± 0.00
	Chemical-GRN-Lung	0.89 ± 0.03	0.14 ± 0.02
	Chemical-GRN-Breast	0.91 ± 0.05	0.12 ± 0.03
Leave cell out	Genetic-GRN-Lung	0.65 ± 0.06	0.01 ± 0.00
	Genetic-GRN-Breast	0.63 ± 0.05	0.01 ± 0.00
	Chemical-GRN-Lung	0.90 ± 0.06	0.10 ± 0.03
	Chemical-GRN-Breast	0.89 ± 0.03	0.13 ± 0.02

Figure S4: PDGRAPHER predicts genetic and chemical perturbagens to shift cells from diseased to treated states using GRNs. **(A)** Shown are performance metrics of PDGRAPHER on Genetic-GRN-Lung, Genetic-GRN-Breast, Chemical-GRN-Lung, and Chemical-GRN-Breast, where we observe similar performance to their counterpart using PPI as the proxy causal graph.

Supplementary references

1. Lotfollahi, M., Wolf, F. A. & Theis, F. J. scGen predicts single-cell perturbation responses. *Nature Methods 2019 16:8* **16**, 715–721 (2019).
